# Detecting gene–environment interactions from multiple continuous traits

**DOI:** 10.1093/bioinformatics/btae419

**Published:** 2024-06-25

**Authors:** Wan-Yu Lin

**Affiliations:** Institute of Health Data Analytics and Statistics, College of Public Health, National Taiwan University, Taipei 100, Taiwan; Master of Public Health Degree Program, College of Public Health, National Taiwan University, Taipei 100, Taiwan

## Abstract

**Motivation:**

Genetic variants present differential effects on humans according to various environmental exposures, the so-called “gene–environment interactions” (GxE). Many diseases can be diagnosed with multiple traits, such as obesity, diabetes, and dyslipidemia. I developed a multivariate scale test (MST) for detecting the GxE of a disease with several continuous traits. Given a significant MST result, I continued to search for which trait and which E enriched the GxE signals. Simulation studies were performed to compare MST with the univariate scale test (UST).

**Results:**

MST can gain more power than UST because of (1) integrating more traits with GxE information and (2) the less harsh penalty on multiple testing. However, if only few traits account for GxE, MST may lose power due to aggregating non-informative traits into the test statistic. As an example, MST was applied to a discovery set of 93 708 Taiwan Biobank (TWB) individuals and a replication set of 25 200 TWB individuals. From among 2 570 487 SNPs with minor allele frequencies ≥5%, MST identified 18 independent variance quantitative trait loci (*P* < 2.4E−9 in the discovery cohort and *P* < 2.8E−5 in the replication cohort) and 41 GxE signals (*P* < .00027) based on eight trait domains (including 29 traits).

**Availability and implementation:**

https://github.com/WanYuLin/Multivariate-scale-test-MST-

## 1 Introduction

Through the era of genome-wide association studies (GWAS), “gene–environment interaction” (GxE) has become an important topic. Genetic materials such as DNA are inherited from parents. However, the effects of genes can be modified by various environmental factors (Es). Discovering GxE is crucial to dissect complex disorders. It will be essential to identify whether environmental factors can attenuate or exacerbate the adverse effects of disease-associated alleles.

Without specifying any E, GxE can still be evaluated through a scale test (Paré *et al.* 2010, Soave *et al.* 2015, Soave and Sun 2017, Staley *et al.* 2022). Heteroscedasticity (non-constant variance) of a phenotype at three genotypes of a single-nucleotide polymorphism (SNP) is a clue of GxE (Wang *et al.* 2019). Testing variance quantitative trait locus (vQTL) provides a systematic and convenient way to search for GxE even when Es are unknown (Paré *et al.* 2010, Struchalin *et al.* 2012, Wang *et al.* 2019, Staley *et al.* 2022, Westerman *et al.* 2022). While Levene’s statistic (Levene 1960) was used to test for equal variance (Paré *et al.* 2010, Wang *et al.* 2019, Westerman *et al.* 2022), a conceptually identical regression framework was developed to allow for continuous exposures (Struchalin *et al.* 2012, Soave *et al.* 2015, Soave and Sun 2017, Staley *et al.* 2022).

A disease can be diagnosed with multiple phenotypes or traits. For example, dyslipidemia can be evaluated through various phenotypes such as triglyceride (TG), total cholesterol (TCHO), low-density lipoprotein cholesterol (LDL), and high-density lipoprotein cholesterol (HDL). Through an extensive vQTL search for 20 cardiometabolic traits from the UK Biobank (UKB), Westerman *et al.* found “pleiotropy” (regarding phenotypic variance) in many loci (Westerman *et al.* 2022). For example, a vQTL near the *APOC1* gene was associated with the variability of four lipid traits (HDL, LDL, TCHO, and TG). This finding highlights the demand and significance of developing a vQTL approach utilizing multivariate traits. The power of detecting this vQTL can be boosted by aggregating the information from multiple lipid levels.

Some GxE methods can handle several traits in the test statistics. For example, Majumdar *et al.* provided a two-step approach to test for GxE from multiple phenotypes, called “MPGE” (multiple phenotypes GxE) (Majumdar *et al.* 2020). When multiple traits provide GxE effect (i.e. GxE pleiotropy), MPGE offers a substantial gain in power compared to the analogous two-step approach for individual traits. Luo *et al.* developed a multi-trait analysis of GxE (MTAGEI) (Luo *et al.* 2024). To use MTAGEI, a continuous environmental variable must be discretized as several environmental groups. GxE can be detected if the genetic effects are heterogeneous across different environmental groups.

Other GxE methods can account for multiple Es. For example, in 2020, Kerin and Marchini developed a Linear Environment Mixed Model Analysis (LEMMA) method to estimate the interactions between genetic variants and an environmental score (ES), where ES was a linear combination of several Es (Kerin and Marchini 2020). Recently, Moore *et al.* proposed a structured linear mixed model (StructLMM) to identify loci interacting with one or multiple Es (Moore *et al.* 2019). They assumed the per-individual allelic effects were random following a multivariate normal distribution. Therefore, GxE can be detected by testing the variance component of the per-individual allelic effects. A non-zero variance implies heterogeneous genetic effects possibly due to GxE (Moore *et al.* 2019).

LEMMA (Kerin and Marchini 2020) and StructLMM (Moore *et al.* 2019) allow for multiple Es but only one trait, whereas MPGE (Majumdar *et al.* 2020) and MTAGEI (Luo *et al.* 2024) allow for multiple traits but only one E. To implement these four GxE methods, E(s) must be prespecified in advance. I developed a multivariate vQTL approach (called MST, multivariate scale test) to allow for multiple traits and unspecified Es. Even if no information on Es is collected, MST can still be performed to explore the possibility of GxE. MST requires less data to test for GxE’s plausibility than other methods.

The four GxE methods (Moore *et al.* 2019, Kerin and Marchini 2020, Majumdar *et al.* 2020, Luo *et al.* 2024) explicitly incorporate GxE information in the model, whereas MST does not require prior knowledge of E. Because MST is a multivariate approach of UST (univariate scale test, the conventional “implicit” test for GxE), the comparison was conducted between MST and UST. Real data applications and simulations demonstrated the utility and performance of the MST approach.

## 2 Methods

### 2.1 Multivariate scale test

Suppose each individual provides *K* continuous traits related to a disease, $Y=\left[\begin{array}{ccc} Y_{1} & \cdots & Y_{K} \end{array}\right]$. I adjusted each trait with covariates such as sex, age, body mass index (BMI), ancestry principal components (PCs), etc. Let ***X*** be the vector of covariates, and $G_{1}$ and $G_{2}$ be two dummy variables coding the three genotypes of an SNP. That is, $\left(G_{1},G_{2}\right)$ would be coded as $\left(0,\;0\right),\;\left(1,\;0\right),\;\mathrm{and}\;\left(0,\;1\right)$ for 0, 1, and 2 minor alleles, respectively. Then, I consider the regression model,

(1) $$E\left(Y_{k}\right)=\alpha_{0,k}+\alpha_{G_{1},k}G_{1}+\alpha_{G_{2},k}G_{2}+\alpha_{X,k}^{T}X,\;\mathrm{where}\;k=1,\ldots ,K.$$

The residuals ($e_{k},\;k=1,\ldots ,K$) from model (1) are the traits adjusted for genotypes and covariates. Suppose there are *n* individuals, the dispersion of residuals is calculated by ${D_{k,i}=\left(e_{k,i}-\tilde{e_{k}}\right)}^{2}$, where $e_{k,i}$ is the residual of the *k*th trait from the *i*th individual (k=1,…,K; i=1,…,n), and $\tilde{e_{k}}$ is the sample median of $e_{k,i}$s across all *n* individuals (the sample median is more robust than the sample mean).

I then tested whether the dispersion measure ($D_{k,i}$) varies with different genotypes, by considering the hypotheses:

$$\left\{\begin{array}{c} H_{0}:D=1\gamma_{0}^{T}+\varepsilon_{0}\;\;(\mathrm{reduced}\;\mathrm{model})\;(2)\\ H_{1}:D=1\gamma_{0}^{T}+G_{1}\gamma_{G_{1}}^{T}+G_{2}\gamma_{G_{2}}^{T}+\varepsilon_{1}\;\;(\mathrm{larger}\;\mathrm{model})\;(3) \end{array}\right.$$

where $D={\left[\begin{array}{ccc} D_{1,1} & \cdots & D_{K,1}\\ \vdots & \ddots & \vdots \\ D_{1,n} & \cdots & D_{K,n} \end{array}\right]}_{n\times K}$, 1 is an n×1 vector with all elements of 1, $\gamma_{0}$ is a K×1 vector of intercept terms, $G_{1}$ and $G_{2}$ are two n×1 dummy-variable vectors coding the three genotypes of an SNP, and $\gamma_{G_{1}}$ and $\gamma_{G_{2}}$ are two K×1 vectors of effect sizes.

In model (2), $\varepsilon_{0}$ is an n×K matrix of error terms under the reduced model, following a multivariate normal distribution with a K×1 mean vector of **0** and a K×K variance–covariance matrix of $\Sigma_{0}$. In model (3), $\varepsilon_{1}$ is an n×K matrix of error terms under the larger model, following a multivariate normal distribution with a K×1 mean vector of **0** and a K×K variance–covariance matrix of $\Sigma_{1}$. In real data analysis, $\Sigma_{0}$ can be estimated by the sum of squared residual vectors under the reduced model, i.e.

(4) $$\hat{\Sigma_{0}}=\sum_{i=1}^{n}R_{i(0)}R_{i(0)}^{T}=\sum_{i=1}^{n}\left(D_{i}-\hat{D_{i(0)}}\right){\left(D_{i}-\hat{D_{i(0)}}\right)}^{T},$$

and $\Sigma_{1}$ can be estimated by the sum of squared residual vectors under the larger model, i.e.

(5) $$\hat{\Sigma_{1}}=\sum_{i=1}^{n}R_{i(1)}R_{i(1)}^{T}=\sum_{i=1}^{n}\left(D_{i}-\hat{D_{i(1)}}\right){\left(D_{i}-\hat{D_{i(1)}}\right)}^{T},$$

where $D_{i}$ is a K×1 vector of dispersion measure of the *i*th individual (with *K* elements $D_{k,i},\;k=1,\ldots ,K$); $\hat{D_{i(0)}}$ and $\hat{D_{i(1)}}$ are K×1 vectors of predicted (or fitted) dispersion measure of the *i*th individual under the reduced and larger models, respectively.

The Pillai’s trace was recommended as the most robust choice among various tests in multivariate analysis of variance (MANOVA) (Olson 1974). I therefore considered the Pillai’s trace statistic (Pillai 1955)

(6) $$V=\mathrm{trace}\left(\left(\hat{\Sigma_{0}}-\hat{\Sigma_{1}}\right){\hat{\Sigma_{0}}}^{-1}\right),$$

where trace indicates the summation of all the diagonal elements of a matrix. The null hypothesis (reduced model) will be rejected if the Pillai’s trace *V* test statistic is large, representing the overall squared residuals under the larger model ($\hat{\Sigma_{1}}$) are smaller than that under the reduced model ($\hat{\Sigma_{0}}$). The Pillai’s trace *V* test statistic (6) can be transformed into an *F* test statistic (Rencher and Christensen 2012), as follows:

(7) $$F=\left(\frac{V}{s-V}\right)\left(\frac{{df}_{2}}{{df}_{1}}\right).$$

Under $H_{0}$, statistic (7) follows the *F* distribution with degrees of freedom of ${df}_{1}=s\times (2t+s+1)$ and ${df}_{2}=s\times (2u+s+1)$, where $s=\min\left(K,\;L-1\right)$, $t=0.5\left(\left|K-L+1\right|-1\right)$, $u=0.5\left(n-L-K-1\right)$ (Rencher and Christensen 2012), and *L* is the number of genotype groups at the SNP (usually *L *=* *3, representing 0, 1, 2 minor alleles).

According to the Bonferroni correction, $H_{0}$ will be rejected if the *P*-value of the *F* test (7) is less than the significance level of .05/M, where *M* is the total number of SNPs analysed by MST. Rejecting $H_{0}$ indicates that the dispersion measure ($D_{k,i},\;\mathrm{where}\;k=1,\ldots ,K\;\mathrm{and}\;i=1,\ldots ,n$) varies with different genotypes, which is a clue of GxE.

### 2.2 Univariate scale test

Given a significant MST test (7), the UST is used to test whether the *k*th trait (*k *=* *1, …, *K*) accounts for the vQTL signal. *H*_0_: The *k*th trait does not provide vQTL signal. *H*_1_: The *k*th trait provides vQTL signal

(8) $$F=\left(\frac{\hat{\Sigma_{0}}[k,k]-\hat{\Sigma_{1}}[k,k]}{\hat{\Sigma_{1}}[k,k]}\right)\left(\frac{{df}_{4}}{{df}_{3}}\right),$$

where $\hat{\Sigma_{0}}[k,k]$ and $\hat{\Sigma_{1}}[k,k]$ represent the *k*th diagonal elements of $\hat{\Sigma_{0}}$ and $\hat{\Sigma_{1}}$, respectively. Under $H_{0}$, statistic (8) follows the *F* distribution with degrees of freedom of ${df}_{3}=L-1$ and ${df}_{4}=n-L$, in which *L* is the number of genotype groups at the SNP (usually *L *=* *3, representing 0, 1, 2 minor alleles). The *k*th trait is considered to provide vQTL signal if the *P*-value of the *F* test (8) is smaller than $.05/M_{1}$, where $M_{1}$ is the number of tests under the *ad hoc* analysis.

### 2.3 Data from the Taiwan Biobank

As of February 2024, 27 675 and 120 161 adults (aged 30–70 years) have been whole-genome genotyped by the TWB 1.0 and TWB 2.0 genotyping arrays, respectively (Wei *et al.* 2021). Running on the Axiom Genome-Wide Array Plate System (Affymetrix, Santa Clara, CA), the TWB 1.0 genotyping array was designed for Taiwan’s Han Chinese and was released in April 2013. The TWB 2.0 genotyping array was later released in August 2018, according to the next-generation sequencing of ∼1000 Taiwan Biobank (TWB) individuals and the experience of developing TWB 1.0.

These 27 675 and 120 161 participants formed the so-called “TWB1” and “TWB2” cohorts. The TWB2 (*n *=* *120,161) and TWB1 (*n *=* *27,675) were regarded as the discovery and replication cohorts, respectively. The TWB excluded samples that were missing more than 2% of their genotype calls. Subsequently, the TWB used the KING (Kinship-based INference for GWAS) software (Manichaikul *et al.* 2010) to estimate the cryptic relatedness among individuals. I removed the individual with a higher missing genotype rate from each relative pair and ensured no pair of subjects was more closely related than the second‐degree relatives. After this step, 25 200 and 93 708 individuals remained in the TWB1 and TWB2 cohorts, respectively.

TWB 1.0 and TWB 2.0 arrays separately comprised 632 172 and 648 542 autosomal SNPs. There were 99 931 SNPs overlapped across the two genotyping arrays. The TWB removed SNPs with genotyping rates <98% or Hardy–Weinberg test *P-*values <1E−10. The PLINK (Purcell *et al.* 2007) command “—indep-pairwise 500 50 0.2” was used to prune SNPs in high linkage disequilibrium (LD). This command excluded SNPs with an *r^2^* > 0.2 within a sliding window of size 500. The TWB shifted the sliding window at each step of 50 SNPs. The remaining nearly independent SNPs (*r^2^* ≤ 0.2 within a sliding window of size 500) were then used to construct ancestry PCs with the PLINK (Purcell *et al.* 2007) command “—pca”.

The TWB used IMPUTE2 (v2.3.1) (Howie *et al.* 2009, Delaneau *et al.* 2013) for genotype imputation. The reference panel was the combination of 1451 TWB individuals with whole-genome sequence data and 504 East Asians (EAS) from the 1000 Genomes Phase 3 v5 (a total of 1955 genomes). As shown by Wei *et al.* (2021), this TWB + EAS panel (*n *=* *1955) provided an improvement in imputation accuracy over the TWB panel (*n *=* *1451) or the EAS panel (*n *=* *504). After imputation, the TWB removed variants with missing rates >5% or minor allele frequencies (MAFs) <0.01%. Variants with an information score <0.3 were also excluded, in which 0.3 was commonly chosen as a threshold for imputation (Kosugi *et al.* 2023). Through these quality control steps, 9 814 944 autosomal variants remained on both the TWB 1.0 and TWB 2.0 arrays.

I analysed 2 570 487 SNPs with MAFs ≥5% in both the TWB2 and TWB1 cohorts. Because 29 continuous traits in eight domains were investigated herein, MST *P*-value <.05/(2 570 487 × 8) = 2.4E−9 or UST *P*-value <.05/(2 570 487 × 29) = 6.7E−10 was considered significant. SNPs with MAFs <5% were skipped from the analysis due to the inferior genotyping (or imputation) accuracy for low-frequency variants (Mitt *et al.* 2017). GxE studies usually focus on common SNPs because of their better reproducibility (Wang *et al.* 2019). If the sample size in any G-by-E combination is small, the evidence of GxE will hardly be replicated by another cohort.

### 2.4 Simulation studies

**Figure 1.**
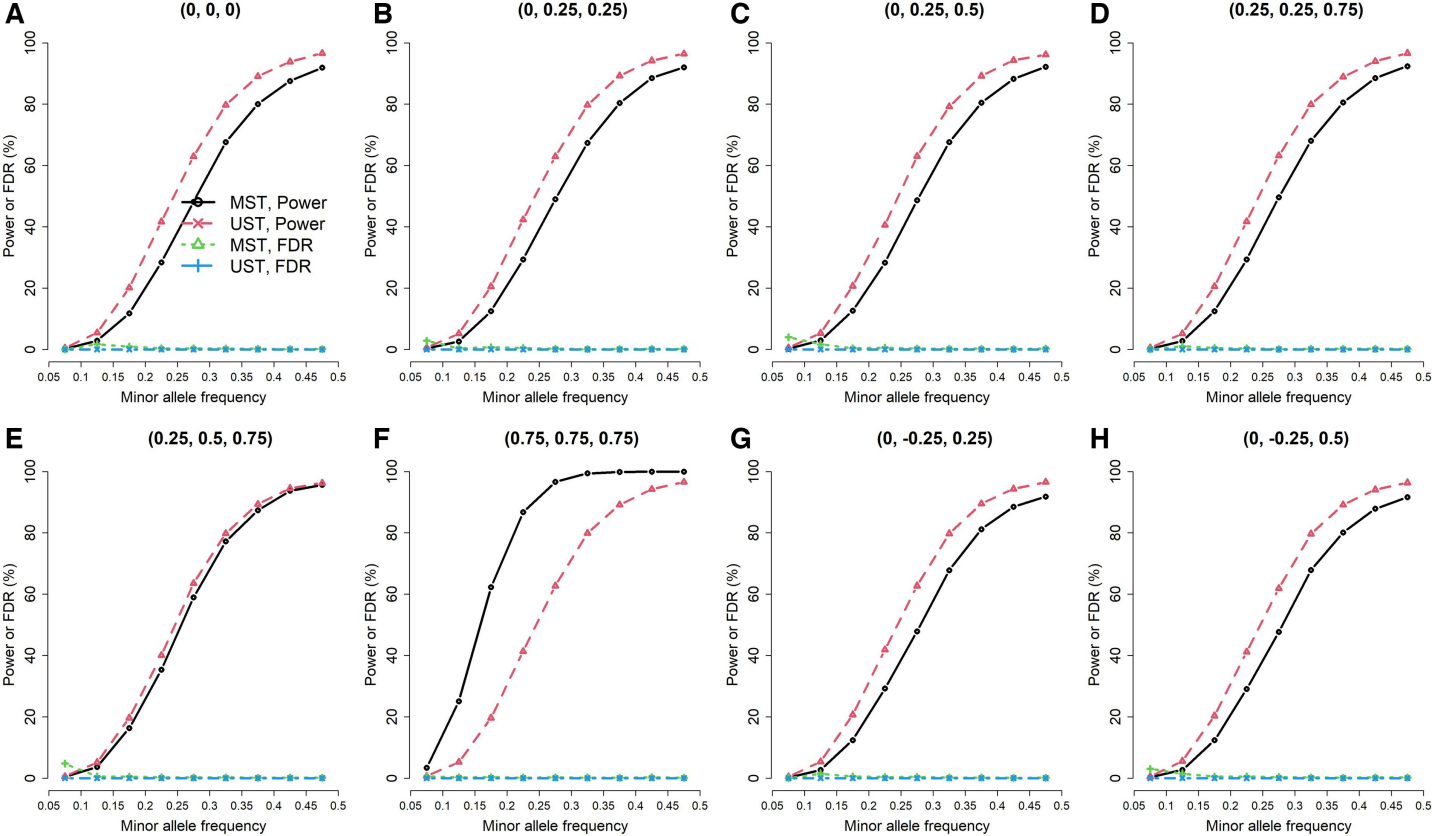
Power and false discovery rate (FDR) of testing GxE for individual traits, when GxE influenced the first trait (three traits from a multivariate normal distribution, exposure prevalence = 0.2, and *n *=* *93 708). Each point was calculated based on 10 000 replications. The title of each plot represents $\left(\rho_{12},\rho_{13},\rho_{23}\right)$

**Figure 2.**
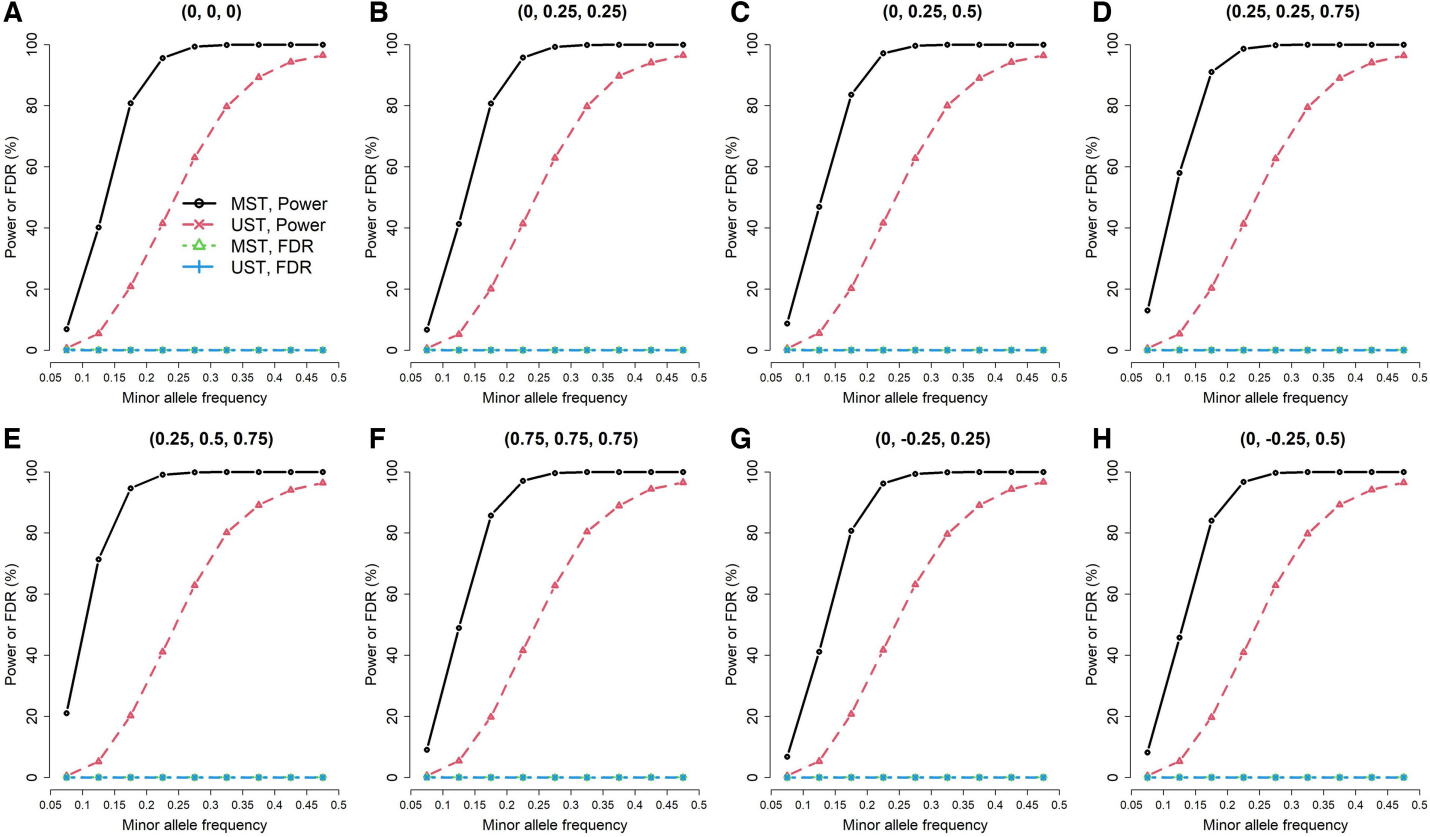
Power and false discovery rate (FDR) of testing GxE for individual traits, when GxE influenced the first and the second traits (three traits from a multivariate normal distribution, exposure prevalence = 0.2, and *n *=* *93 708)

**Figure 3.**
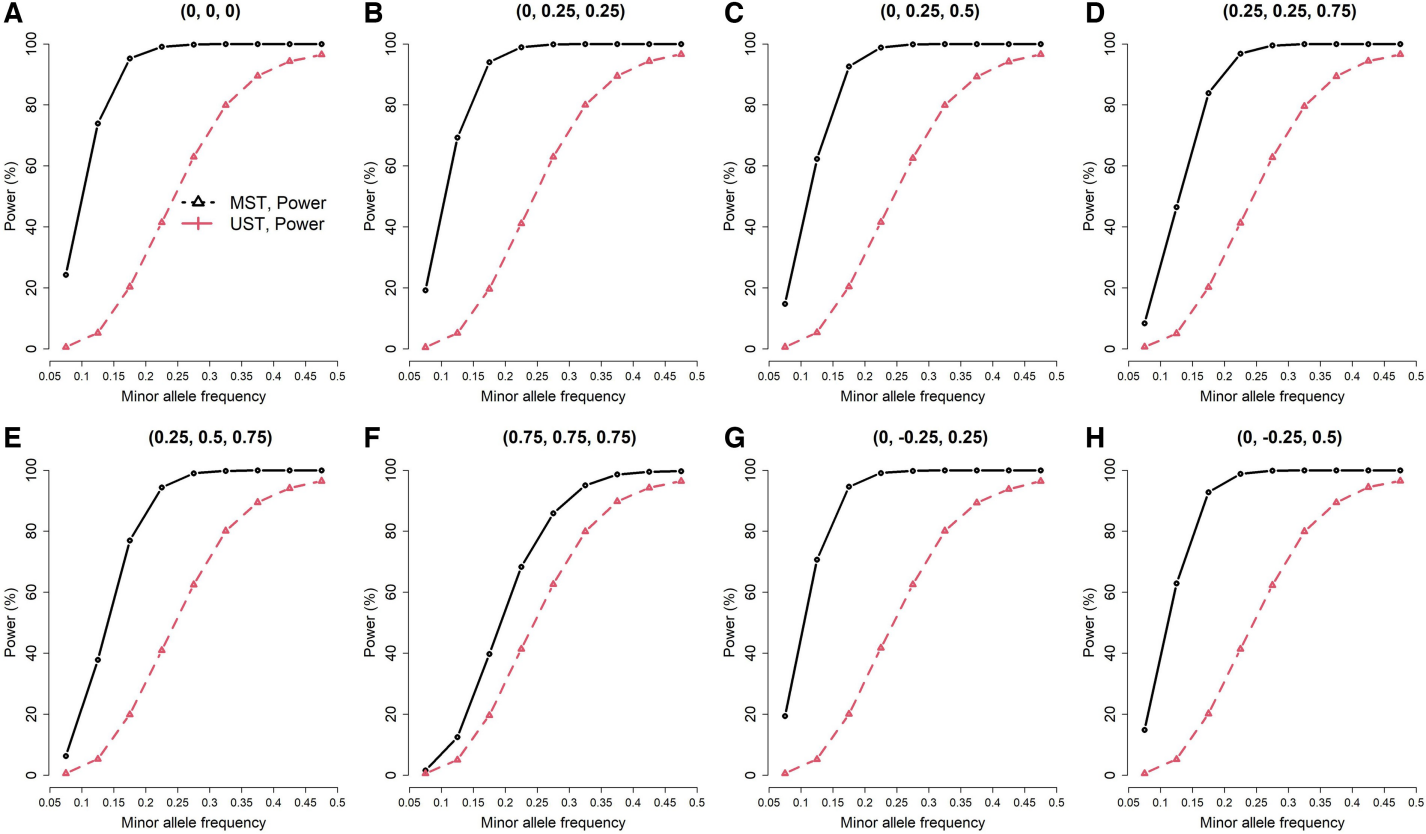
Power of testing GxE for individual traits, when GxE influenced all three traits (three traits from a multivariate normal distribution, exposure prevalence = 0.2, and *n *=* *93 708)

To reflect the performance under real genotype data, I first used TWB2 (the discovery cohort) SNPs to generate traits as follows:

(9) $$Y_{i}=\beta_{G}G_{i}+\beta_{E}E_{i}+\beta_{\mathrm{INT}}G_{i}\times E_{i}+\varepsilon_{i},\;i=1,\ldots ,\;93\;708,$$

where $G_{i}=0$, 1, 2 representing the number of minor alleles. The environmental factor $E_{i}$ was randomly sampled from 1 (exposed) or 0 (non-exposed), with the “exposure prevalence” *P*($E_{i}$ = 1) = 0.2 or 0.5, for i=1,…, 93 708. The random error term $\varepsilon_{i}$ was generated from a multivariate normal distribution with a 3×1 mean vector of **0** and a 3×3 variance–covariance matrix as follows:

(10) $$\mathrm{var}\left(\varepsilon_{i}\right)=\left[\begin{array}{ccc} 1 & \rho_{12} & \rho_{13}\\ \rho_{12} & 1 & \rho_{23}\\ \rho_{13} & \rho_{23} & 1 \end{array}\right],\;i=1,\ldots ,\;93\;708,$$

where $\rho_{12}$ denoted the correlation between the 1st and the 2nd traits, $\rho_{13}$ indicated the correlation between the 1st and the 3rd traits, and $\rho_{23}$ represented the correlation between the 2nd and the 3rd traits, respectively. Eight correlation scenarios listed in the titles of Figs 1–3$\left(\rho_{12},\rho_{13},\rho_{23}\right)$ were evaluated. If I use $\left(\rho_{12}+\rho_{13}+\rho_{23}\right)$ as an overall measure of the correlations among the three traits, the correlation strength can be ordered as (F) > (E) > (D) > (C) > (B) > (A). The other two scenarios, (G) and (H), were used to mimic some traits to be inversely correlated with others. For example, HDL is usually negatively associated with LDL and TG (Jeppesen *et al.* 1997).

#### 2.4.1 *P*-values under the null hypothesis of no GxE

By fixing $\beta_{\mathrm{INT}}=0$ and $\beta_{G}=\beta_{E}=0.3$, I investigated the *P*-values under the null hypothesis of no GxE. Titles of Figs 1–3 present the eight scenarios of the correlations among traits. SNPs were categorized as nine MAF ranges: [0.05, 0.10) (indicating 0.05 ≤ MAF < 0.10), [0.10, 0.15), [0.15, 0.20), [0.20, 0.25), [0.25, 0.30), [0.30, 0.35), [0.35, 0.40), [0.40, 0.45), and [0.45, 0.50] (indicating 0.45 ≤ MAF ≤ 0.50). Eight correlation scenarios and nine MAF ranges constructed 72 combinations. Each combination was evaluated with 10 000 replications. For example, for the MAF range [0.05, 0.10), the leading 10 000 SNPs on chromosome 1 (i.e. the 10 000 SNPs with the smallest base pairs) satisfying 0.05 ≤ MAF < 0.10 were used for simulation.

#### 2.4.2 Power of testing GxE for individual traits

By fixing $\beta_{\mathrm{INT}}=0.3$ and $\beta_{G}=\beta_{E}=0.3$, I presented the statistical power given the significance level of 2.4E−9 (for MST) or 6.7E−10 (for UST). Many vQTL researches have been conducted on multiple traits and on a genome-wide scale (Wang *et al.* 2019, Shi 2022, Westerman *et al.* 2022, Lin 2024). To evaluate the performance of the two methods in this situation, I adopted the significance threshold from the real genome-wide vQTL search. The same 72 combinations were evaluated under $\beta_{\mathrm{INT}}=0.3$ for power comparison. Similarly, each combination was simulated with 10 000 replications.

MST is a two-stage procedure. If the MST test statistic [Equation (7)] is significant (*P *<* *2.4E−9) and the *ad hoc* analysis for the *k*th trait [Equation (8)] is significant (*P* < $.05/M_{1}$, where $M_{1}$ is the number of tests under the *ad hoc* analysis), the *k*th trait is considered to provide vQTL signal. By contrast, UST is a one-stage procedure. If the UST statistic [Equation (8)] is significant (*P *<* *6.7E−10) for the *k*th trait (*k *=* *1, …, *K*), the *k*th trait is claimed to provide vQTL signal.

#### 2.4.3 Simulation under a smaller sample size

In addition to the TWB2 (the discovery cohort) SNPs, I also used the TWB1 (the replication cohort) SNPs as the simulation materials. With a sample size of 25 200, this simulation compared MST with UST in a smaller GWAS. Except for a smaller sample size (*n *=* *25 200), all simulation settings were identical to those for TWB2 (*n *=* *93 708).

#### 2.4.4 Simulation given four traits

Moreover, I also considered the situation given more traits. The random error term $\varepsilon_{i}$ was generated from a multivariate normal distribution with a 4×1 mean vector of **0** and a 4×4 variance-covariance matrix as follows:

(11) $$\mathrm{var}\left(\varepsilon_{i}\right)=\left[\begin{array}{ccc} 1 & \cdots & \rho_{14}\\ \vdots & \ddots & \vdots \\ \rho_{14} & \cdots & 1 \end{array}\right],\;i=1,\ldots ,\;93\;708\;\mathrm{or}\;25\;200.$$

Eight correlation scenarios listed in the title of Supplementary Fig. S30 were evaluated. Six correlations were required in Equation (11), $\left(\rho_{12},\rho_{13},\rho_{14},\rho_{23},\rho_{24},\rho_{34}\right)$, where $\rho_{uv}$ was the correlation between the *u*th and the *v*th traits, and *u, v* $\in \left\{1,\;2,\;3,\;4\right\}$.

#### 2.4.5 Simulation given right-skewed traits

By squaring $\varepsilon_{i}$, I considered the error term coming from a chi-squared distribution with the degree of freedom 1, as follows:

(12) $$Y_{i}=\beta_{G}G_{i}+\beta_{E}E_{i}+\beta_{\mathrm{INT}}G_{i}\times E_{i}+\nu \varepsilon_{i}^{2},\;i=1,\ldots ,\;93\;708\;\mathrm{or}\;25\;200.$$

This simulation tested the robustness of MST when analysing right-skewed traits such as TG and fasting glucose (FG). The constant ν was used to adjust the power. The power of MST and UST decreased with an increasing ν or an increasing error term. Nonetheless, the relative performance of the two methods remained the same. Without loss of generality, I used ν=0.5 throughout the simulation.

When I simulated three traits, the random error term $\varepsilon_{i}$ was generated from a multivariate normal distribution with a 3×1 mean vector of **0** and a 3×3 variance–covariance matrix as follows:

(13) $$\mathrm{var}\left(\varepsilon_{i}\right)=\left[\begin{array}{ccc} 1 & \sqrt{\rho_{12}} & \sqrt{\rho_{13}}\\ \sqrt{\rho_{12}} & 1 & \sqrt{\rho_{23}}\\ \sqrt{\rho_{13}} & \sqrt{\rho_{23}} & 1 \end{array}\right],\;i=1,\ldots ,\;93\;708\;\mathrm{or}\;25\;200.$$

After squaring $\varepsilon_{i}$ as shown in Equation (12), the correlation between the *u*th and the *v*th traits became $\rho_{uv}$ (*u*, *v *=* *1, 2, 3). Similarly, when I simulated four traits, the random error term $\varepsilon_{i}$ was generated from a multivariate normal distribution with a 4×1 mean vector of **0** and a 4×4 variance-covariance matrix as follows:

(14) $$\mathrm{var}\left(\varepsilon_{i}\right)=\left[\begin{array}{ccc} 1 & \cdots & \sqrt{\rho_{14}}\\ \vdots & \ddots & \vdots \\ \sqrt{\rho_{14}} & \cdots & 1 \end{array}\right],\;i=1,\ldots ,\;93\;708\;\mathrm{or}\;25\;200.$$

After squaring $\varepsilon_{i}$ as shown in Equation (12), the correlation between the *u*th and the *v*th traits was maintained at $\rho_{uv}$ (*u*, *v *=* *1, 2, 3, 4).

## 3 Results

### 3.1 Simulation results

#### 3.1.1 *P*-values under the null hypothesis of no GxE

I used simulations to evaluate the performance of MST and UST, given the genotypes of 90 000 SNPs on chromosome 1 (10 000 SNPs for each MAF range). Supplementary Figs S1–S8 in the Supplementary Data demonstrate the quantile–quantile (QQ) plots stratified by the nine ranges of MAFs, where the exposure prevalence is 0.2, *n *=* *93 708 (using the TWB2 SNPs) or 25 200 (using the TWB1 SNPs), number of traits = 3 or 4, and traits followed a multivariate normal distribution or the chi-squared distributions with the degree of freedom 1. The QQ plots under different correlation scenarios were similar, so I combined the results from various correlation scenarios into a plot. When the traits were normally distributed (Supplementary Figs S1, S3, S5, and S7), the observed *P*-values matched the expected *P*-values under the null hypothesis (without GxE in any of the three/four traits). When the traits followed the chi-squared distribution with the degree of freedom 1, deflation of *P*-values was observed at SNPs with MAFs <0.10 in larger data sets (*n *=* *93 708) [Supplementary Figs S2(1) and S6(1)] and at SNPs with MAFs <0.15 in smaller data sets (*n *=* *25 200) [Supplementary Figs S4(1, 2) and S8(1, 2)]. However, fortunately, even for right-skewed traits like chi-squared distributions with the degree of freedom 1 (skewness = 2.8, excess kurtosis = kurtosis − 3 = 12), common variants with MAFs ≥0.10 (for *n* ∼ 93 708) or ≥0.15 (for *n* ∼ 25 200) can still be analysed by MST or UST.

#### 3.1.2 Power and false discovery rate (FDR) of testing GxE for individual traits

To compare MST and UST in testing GxE for individual traits, I calculated the power and FDR under each scenario. To reflect the performance of MST in a genome-wide vQTL search, I used 0.05/65= 0.00077 as the significance level of MST’s *ad hoc* analysis, where 65 was the number of tests under MST’s *ad hoc* analysis in the real data analysis of this work (Table 1). That is, after a genome-wide vQTL search using MST, I subsequently performed 65 USTs [Equation (8)] to figure out which individual traits accounted for the vQTL signals. As I was evaluating the performance of MST on a genome-wide scale, the only reference to the number of tests under MST’s *ad hoc* analysis was “65”, which came from this study. FDR is the proportion of falsely detecting GxE among all discoveries. Given three traits simulated from the multivariate normal distribution, exposure prevalence = 0.2, and *n *=* *93 708, Figs 1–3 present the power and FDR when GxE influenced one, two, and all three traits. Each figure contained 72 combinations of correlation settings (A, B, …, H plots) and MAF scenarios (*x*-axis). Each of the 72 scenarios was evaluated with 10 000 replications. As expected, the power of both methods increased with a larger MAF (Figs 1–3).

**Table 1.** The 18 independent variance quantitative trait loci detected by MST.

Four lipids traits (24 tests under MST’s *ad hoc* analysis)									
Chr.	BP	SNP	MAF (TWB2/TWB1)^a^	Gene	MST *p* (TWB2/TWB1)^b^	HDL UST *P*^c^	LDL UST *P*^c^	TCHO UST *P*^c^	TG UST *P*^c^
2	21024193	rs57825321	0.148/0.146	*APOB*	**3.3E−22/1.0E−5**	6.8E−3/.89	**6.7E−25/1.8E−7** ^d^	**9.3E−16/3.8E−6** ^d^	.03/.03^d^
11	116792991	rs662799	0.274/0.275	*APOA5*	**7.8E−215/1.9E−70**	.01/.09^d^	**5.5E−12/1.9E−3** ^d^	**1.7E−12/2.3E−3** ^d^	**9.9E−213/4.0E−73** ^d^
15	58400418	rs60900172	0.356/0.357	*ALDH1A2*	**1.2E−11/2.3E−5**	**2.6E−6/5.2E−5** ^d^	.16/.84	**1.3E−4/.09** ^d^	.06/.03^d^
16	56956804	rs247617	0.161/0.160	*CETP*	**6.3E−44/1.6E−7**	**2.7E−48/1.6E−8** ^d^ ^,^ ^e^	.32/.20	.08/.18^d^	.15/.05^d^
19	44888997	rs6857	0.083/0.084	*NECTIN2*	**1.1E−10/2.4E−7**	.03/.10^d^	**6.3E−5/4.6E−5** ^d^	.03/9.4E−3^d^	**1.8E−10/2.7E−4** ^d^
19	44923535	rs141622900	0.075/0.068	*APOC1*	**5.4E−126/1.9E−26**	**1.1E−9/2.7E−4** ^d^ ^,^ ^e^	**3.5E−38/1.5E−9** ^d^ ^,^ ^e^	**1.7E−13/.01** ^d^ ^,^ ^e^	**1.2E−15/.10** ^d^ ^,^ ^e^

Five blood traits (25 tests under MST’s *ad hoc* analysis)										
Chr.	BP	SNP	MAF^a^	Gene	MST *P*^2^	RBC UST *P*^c^	WBC UST *P*^c^	Platelet UST *P*^c^	HB UST *P*^c^	HCT UST *P*^c^
16	250642	rs9940149	0.446/0.449	*FAM234A*	**3.4E−137/3.7E−39**	**3.3E−136/3.8E−44** ^d^	.99/.24	.21/.28	3.8E−3/6.4E−3^d^	.35/.02
16	319562	rs28461430	0.127/0.129	*AXIN1*	**5.0E−12/2.8E−5**	**1.8E−13/2.8E−7** ^d^	.13/.67	.92/.15	.64/.96	.12/.65
16	418055	rs62030830	0.488/0.488	*DECR2*	**1.2E−57/9.9E−16**	**9.7E−62/1.6E−17** ^d^	.57/.39	.80/.47	.10/.31^d^	.67/.21
16	489088	rs3830847	0.439/0.442	*RAB11FIP3*	**2.1E−38/2.0E−7**	**1.2E−39/9.0E−11** ^d^	.08/.64	.74/.69	.02/.61^d^	.71/.57
16	595968	rs4144003	0.395/0.397	*RAB40C*	**3.5E−41/1.5E−10**	**6.0E−46/3.8E−12** ^d^	.82/.51	.19/.40	.36/.54^d^	.80/.10^d^

Three kidney traits (six tests under MST’s *ad hoc* analysis)								
Chr.	BP	SNP	MAF^a^	Gene	MST *P*^b^	Creatinine UST *P*^c^	UA UST *P*^c^	BUN UST *P*^c^
4	9982917	rs9994216	0.406/0.410	*SLC2A9*	**1.5E−22/1.3E−5**	.86/.47	**1.2E−23/5.7E−6** ^d^	.56/.35
4	88122482	rs45499402	0.314/0.319	*ABCG2*	**3.9E−62/3.4E−24**	.05/.12^d^	**2.3E−62/3.9E−27** ^d^	.08/.68^d^

Two liver traits (10 tests under MST’s *ad hoc* analysis)							
Chr.	BP	SNP	MAF^a^	Gene	MST *P*^b^	TB UST *P*^c^	Albumin UST *P*^c^
2	233347393	rs13012213	0.177/0.179	*SAG*	**7.9E−23/1.7E−5**	**8.7E−25/1.0E−6** ^d^	.63/.94
2	233679061	rs10202865	0.189/0.188	*UGT1A10*	**4.6E−156/2.8E−52**	**1.3E−158/5.5E−54** ^d^ ^,^ ^e^	.48/.48
2	233715640	rs6749496	0.114/0.112	*UGT1A10*	**0/7.9E−128**	**0/1.1E−130** ^d^ ^,^ ^e^	.05/.80
2	233842243	rs3806588	0.181/0.190	*HJURP*	**2.7E−16/5.7E−6**	**3.6E−17/4.5E−7** ^d^	.16/.75
12	20844459	rs4341591	0.157/0.157	*SLCO1B3*	**3.9E−21/1.6E−6**	**1.0E−22/2.0E−7** ^d^	.95/.48

a MAF = minor allele frequency.

b MST *P*-values (TWB2/TWB1) were highlighted in bold type if TWB2 MST *P *<* *2.4E−9 and TWB1 MST *P *<* *.05/1767 = 2.8E−5.

c Twenty-six trait-vQTL combinations’ *P*-values (TWB2/TWB1) were highlighted in bold type because of UST *P*-value < .05/65 = .00077 in TWB2 or TWB1, where 65 is the number of tests under the *ad hoc* analysis. Further analysis for these 26 trait-vQTL combinations is shown in the left column of Fig. 5.

d QTL (quantitative trait locus): if the trait value was associated with the two dummy variables coding the three genotypes of the SNP (*P*-value < .05/65 = .00077 in TWB2), the SNP was denoted as a QTL. From this table, all vQTLs were QTLs, but not all QTLs were vQTLs.

e vQTLs that were also identified by Westerman *et al.* (2022) (European population data: 350 016 unrelated participants in the UK Biobank).

When GxE influenced the first trait (Fig. 1), except in the high correlations among traits (F), UST had a larger power and a smaller FDR than MST. Scenario (F) indicates that the pairwise correlations between any two traits are high ($\rho_{12}=\rho_{13}=\rho_{23}=$ 0.75); the second and the third traits can borrow the strength from the first trait. This increased the power of the MST statistic. The FDRs of UST were all 0%, whereas the FDRs of MST were controlled under 4.8% (detailed FDRs are listed in Table S1 of the Supplementary Data). Because the actual situation is that GxE influenced the first trait, FDR is the proportion of falsely detecting GxE from the second or third trait among all discoveries.

When GxE influenced the first and the second traits (Fig. 2), MST consistently outperformed UST across all correlation and MAF scenarios. MST had a higher power in detecting GxE than UST while controlling the FDR at a similar level. The FDRs of UST were all 0%, whereas the FDRs of MST were controlled under 0.3% (detailed FDRs are listed in Table S2 of the Supplementary Data). Because the actual situation is that GxE influenced the first and the second traits, FDR is the proportion of falsely detecting GxE from the third trait among all discoveries.

When GxE influenced all three traits (Fig. 3), MST was still more powerful than UST. No FDR can be shown in this situation because all three traits were specified to enrich the GxE signals. Three highly correlated traits with GxE may interfere with each other, and therefore, the power gain of MST over UST is marginal in Fig. 3 (F). This reasonable result can be explained from the MANOVA tests. Recall that the high correlation between dependent variables (here, phenotypes) will reduce the power of the MANOVA tests (Foster *et al.* 2006). Ideally, dependent variables suitable for MANOVA analysis are low to moderately correlated with each other (Foster *et al.* 2006). It makes no sense to put dependent variables measuring the same aspect of the outcome into the model. If the phenotypes are highly correlated, there are better ways to integrate their information, such as simple summation or principal component analysis.

Supplementary Figs S9–S11 show the power and FDR when the three traits were simulated from chi-squared distribution with the degree of freedom 1 (exposure prevalence = 0.2 and *n *=* *93708). The comparison between MST and UST was similar to that for the multivariate normal distributed traits. Supplementary Figs S12–S17 demonstrate the results for exposure prevalence = 0.5. As expected, the power of both methods was increased with a larger exposure prevalence. Nonetheless, the comparison between MST and UST was similar to that for the exposure prevalence of 0.2.

Supplementary Figs S18–S29 show the simulation results given a smaller sample size (*n *=* *25 200). When traits were from chi-squared distribution with the degree of freedom 1, both MST and UST suffered from larger FDR at SNPs’ MAFs <0.15. This corresponded to the deflation of *P*-values at SNPs with MAFs <0.15 in smaller data sets (*n *=* *25 200) [Supplementary Figs S4(1, 2) & S8(1, 2)].

When *n *=* *25 200 and the exposure prevalence was 0.2, both methods had almost no power (Supplementary Figs S18–S23). This is a reasonable result because the scale test (including UST and MST) is an implicit test for GxE identification. No Es are specified or used in the test statistics. Therefore, a larger sample size is required to boost the power. This is why many previous applications of UST focused on the UKB with a larger sample size [e.g. *n *=* *348 501 (Wang *et al.* 2019), *n *=* *350 016 (Westerman *et al.* 2022), and *n *=* *396 077 (Shi 2022)].

Supplementary Figs S30–S61 present the simulation results of the given four traits. If only one trait was influenced by GxE, UST outperformed MST except for the scenario (F) of highly correlated traits (Supplementary Figs S30, S31, S38, S39, S46, S54, and S55). When two or more traits accounted for GxE, MST was more powerful than UST. Similarly, when *n *=* *25 200 and the exposure prevalence was 0.2, both methods had almost no power (Supplementary Figs S46–S53). To sum up, the simulation results for four traits were similar to those for three traits.

To conclude, MST can gain more power than UST because of (1) integrating more traits with GxE information and (2) the less harsh penalty on multiple testing. However, if only few traits account for GxE, MST may lose power and suffer from a larger FDR due to aggregating more non-informative traits into the test statistic (Fig. 1).

Supplementary Table S3 shows the average memory usage and time consumption of MST and UST, in which simulation data of the two levels of exposure prevalence (0.2 and 0.5) were combined. On average, MST occupied less memory and spent a shorter time than UST, where the memory usage was measured by the “summary prof” R function, and the execution time was measured in R (version 4.2.3) on a Windows system running at 3.40 GHz and 64 GB of RAM.

### 3.2 Genome-wide vQTL search for 29 TWB continuous traits

I performed a genome-wide vQTL search for 29 TWB continuous traits using MST and UST. A discovery cohort of 93 708 (called “TWB2”) and a replication cohort of 25 200 individuals (called “TWB1”) were analysed, respectively. MST and UST were applied to 2 570 487 SNPs with MAFs ≥5% in both TWB2 and TWB1. GxE is to explore the impacts of joint distribution between genetic variants and E on phenotypes. If the sample size of any G-by-E combination is too small, this GxE signal is unreliable and can hardly be replicated in another cohort. Therefore, GxE studies usually investigate common SNPs. For example, the systematic vQTL and GxE search of 13 UKB continuous traits also focused on common variants (MAFs ≥5%) (Wang *et al.* 2019). The sample size of TWB2 (*n *=* *93 708) was smaller than that of the UKB study [*n *=* *348 501 (Wang *et al.* 2019)]. Therefore, I also adopted 5% as the MAF cutoff. A total of 29 TWB traits in eight domains were investigated herein, including (A) six lung function traits: vital capacity, tidal volume, inspiratory reserve volume, expiratory reserve volume, forced vital capacity (FVC), and forced expiratory volume in 1 s (FEV1); (B) four lipid traits: HDL, LDL, TCHO, and TG; (C) five obesity traits: BMI, body fat percentage (BFP), waist circumference (WC), hip circumference (HC), and waist–hip ratio (WHR); (D) five blood traits: red blood cells (RBC), white blood cells (WBC), platelets, hemoglobin (HB), and hematocrit (HCT); (E) three kidney traits: creatinine, uric acid (UA), blood urea nitrogen; (F) two liver traits: total bilirubin (TB) and albumin; (G) two hypertension traits: diastolic and systolic blood pressure levels; (H) two diabetes traits: FG and glycated hemoglobin (HbA1c).

In all analyses, covariates adjusted included sex (male versus female), age (in years), BMI (in kg/m^2^), current smoking status (yes versus no), current drinking status (yes versus no), performing physical exercise (yes versus no), educational attainment (integer ranging from 1 to 7), and the first 10 ancestry PCs. Current smoking indicated “having smoked cigarettes for at least 6 months and having not quit smoking when joining the TWB”. Drinking was defined as “having a weekly intake of more than 150 mL of alcoholic beverages for at least 6 months and having not stopped drinking when joining the TWB”. Regular exercise was defined as “performing exercise lasting for 30 min thrice a week”. Educational attainment was an integer ranging from 1 to 7: 1 (illiterate), 2 (no formal education but literate), 3 (primary school graduate), 4 (junior high school graduate), 5 (senior high school graduate), 6 (college graduate), and 7 (Master’s or higher degree). When analysing the five obesity traits (BMI, BFP, WC, HC, and WHR), BMI was excluded from the covariates.

The residuals ($e_{k},\;k=1,\ldots ,K$) from model (1) are the traits adjusted for genotypes and covariates. To remove outliers, I excluded individuals with the residuals ($e_{k},\;k=1,\ldots ,K$) more than 5 standard deviations from the mean. Supplementary Table S4 lists the skewness and “excess kurtosis” (kurtosis – 3) of the 29 TWB traits. Data can be considered normally distributed if the skewness ranges from −2 to 2 and the excess kurtosis ranges from −7 to 7 (Byrne 2010, Hair *et al.* 2010). Most TWB traits met this criterion. Simulation results reminded concerns about false positives at MAFs <0.10 [Supplementary Figs S2(1) and S6(1)] when *n *=* *93 708 and traits followed chi-squared distribution with the degree of freedom 1 (skewness = $\sqrt{8}$ = 2.8; excess kurtosis = 12). Only the two diabetes traits (FG and HbA1c) presented such extreme measures of moments as a chi-squared distribution. However, no discoveries were found from the two diabetes traits.

**Figure 4.**
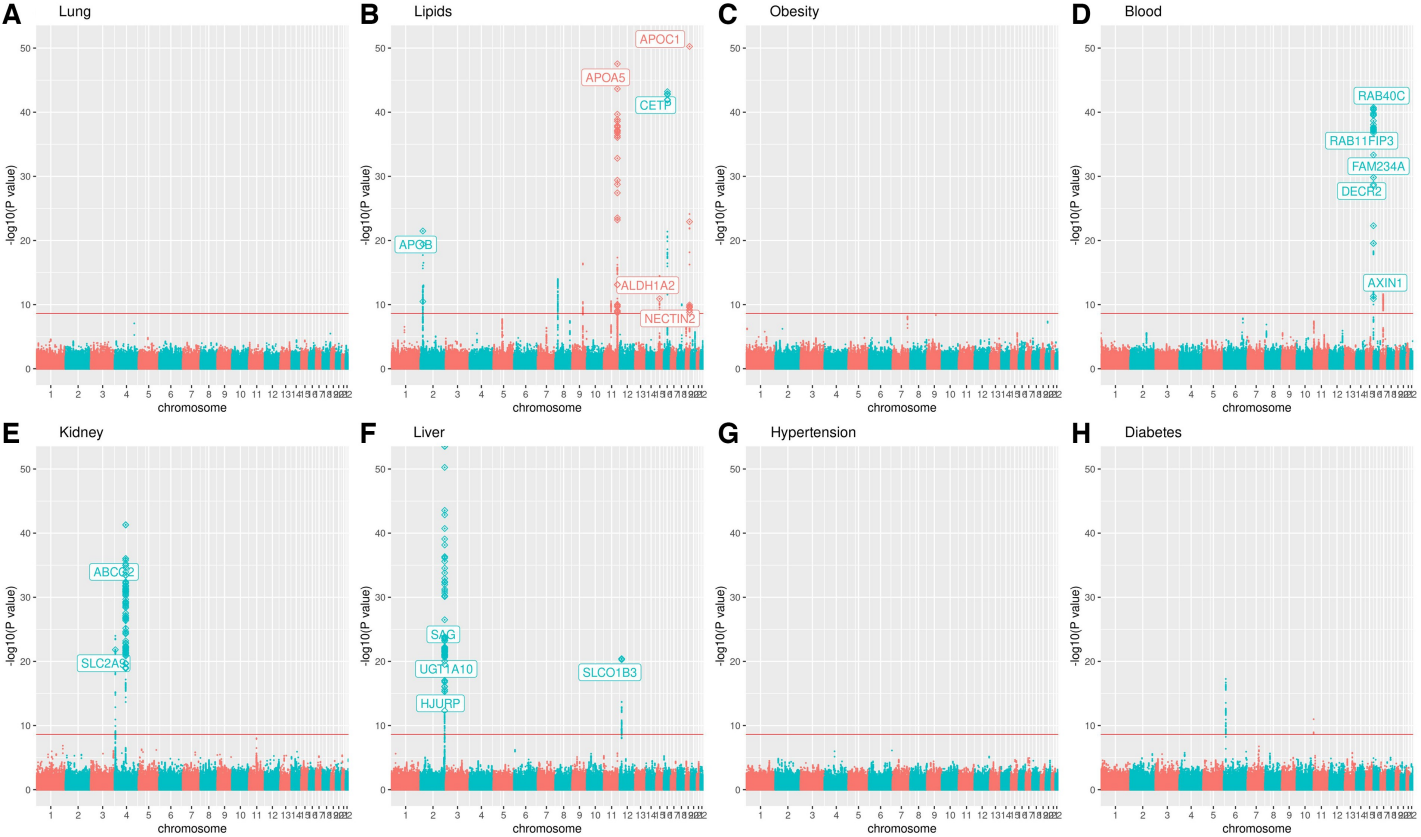
The Manhattan plots of the MST analysis on eight domains of TWB2 continuous traits. The horizontal line denotes the experiment-wise significance level of .05/(2 570 487 × 8) = 2.4E−9, where 2 570 487 is the number of autosomal SNPs with MAF ≥0.05. I identified 1767 vQTLs from TWB2 (MST *P* < 2.4E−9) and sought replication from TWB1 if MST *P* < 0.05/1767 = 2.8E−5. Gene names on this figure mark the 18 independent vQTLs (LD measure *r*^2^ < 0.01) that were identified from TWB2 (MST *P* < 2.4E−9) and further replicated in TWB1 (MST *P* < .05/1767 = 2.8E−5)

Figure 4 shows the Manhattan plots of the MST analysis on the eight domains of traits using the discovery cohort (TWB2) data. The red horizontal line denotes the experiment-wise significance level of .05/(2 570 487 × 8) = 2.4E−9, where 2 570 487 is the number of autosomal SNPs with MAFs ≥0.05 in both TWB2 and TWB1. This experiment-wise significance level is based on the Bonferroni correction, which may be conservative due to the correlations among the 2 570 487 SNPs (Conneely and Boehnke 2007). Nonetheless, I still adopted this significance level to reduce costs derived from false positives. I identified 1767 vQTLs from TWB2 (MST *P *<* *2.4E−9) and sought replication from TWB1 if MST *P *<* *.05/1767 = 2.8E−5. Many of these vQTLs were highly correlated. Through the PLINK clumping procedure (Purcell *et al.* 2007), I found 18 independent vQTLs with LD measure *r^2^* < 0.01. Figure 4 marks the gene names of these 18 independent vQTLs, and Table 1 lists their detailed information.

Supplementary Figs S62–S64 present the Manhattan plots of the UST analysis on the 29 individual traits using the discovery cohort (TWB2) data. The red horizontal line denotes the experiment-wise significance level of .05/(2 570 487 × 29) = 6.7E−10, where 2 570 487 is the number of autosomal SNPs with MAF ≥ 0.05. I identified 1904 vQTLs from TWB2 (UST *P *<* *6.7E−10) and sought replication from TWB1 if UST *P *<* *.05/1904 = 2.6E−5. Many of these vQTLs were highly correlated. Through the PLINK clumping procedure (Purcell *et al.* 2007), I found 18 vQTLs. Supplementary Figs S62–S64 mark the gene names of these vQTLs, and Supplementary Table S5 lists their detailed information.

Although UST (18 SNPs) seemed to explore the same number of vQTL SNPs as MST (18 SNPs), it did not mean that UST was as powerful as MST. The UST vQTL SNPs identified from each trait were independent of each other (*r*^2^ < 0.01). However, UST vQTLs found from a trait may be in high LD with vQTLs detected from another trait in the same domain. For example, rs483082 (in *APOC1*, vQTL of TG) and rs438811 (in *APOC1*, vQTL of LDL) were in high LD with *r*^2^ = 0.99. By excluding the correlated SNPs (*r*^2^ > 0.01), MST found one more vQTL than UST, i.e. the *NECTIN2* gene (also known as the *PVRL2* gene) for lipid traits (Fig. 4B). *NECTIN2* has been reported to play essential roles in lipid metabolism and dyslipidemia (Miao *et al.* 2018, Li *et al.* 2021).

Except for *NECTIN2*, vQTLs identified by MST (Table 1) and UST (Supplementary Table S5) were overlapped or highly correlated. These vQTLs are all located in well-known genes (Hubacek *et al.* 2008, Woodward 2015). The *NECTIN2* gene as a vQTL of lipid traits was found solely by MST. As demonstrated by the simulation study, MST is more powerful than UST, given two out of four traits (2/4) influenced by GxE (Supplementary Figs S32–S33, S40–S41, S48, and S56–S57). The *NECTIN2* gene was likely a vQTL for both LDL and TG, although the UST *P*-values were not smaller than the two (UST) significance levels simultaneously (TWB2 α = 6.7E−10; TWB1 α = 2.6E−5, as shown in note 2 under Supplementary Table S5). Aggregating the information on these lipid traits boosted the statistical power of MST.

### 3.3 vQTLs enriched with GxE

**Figure 5.**
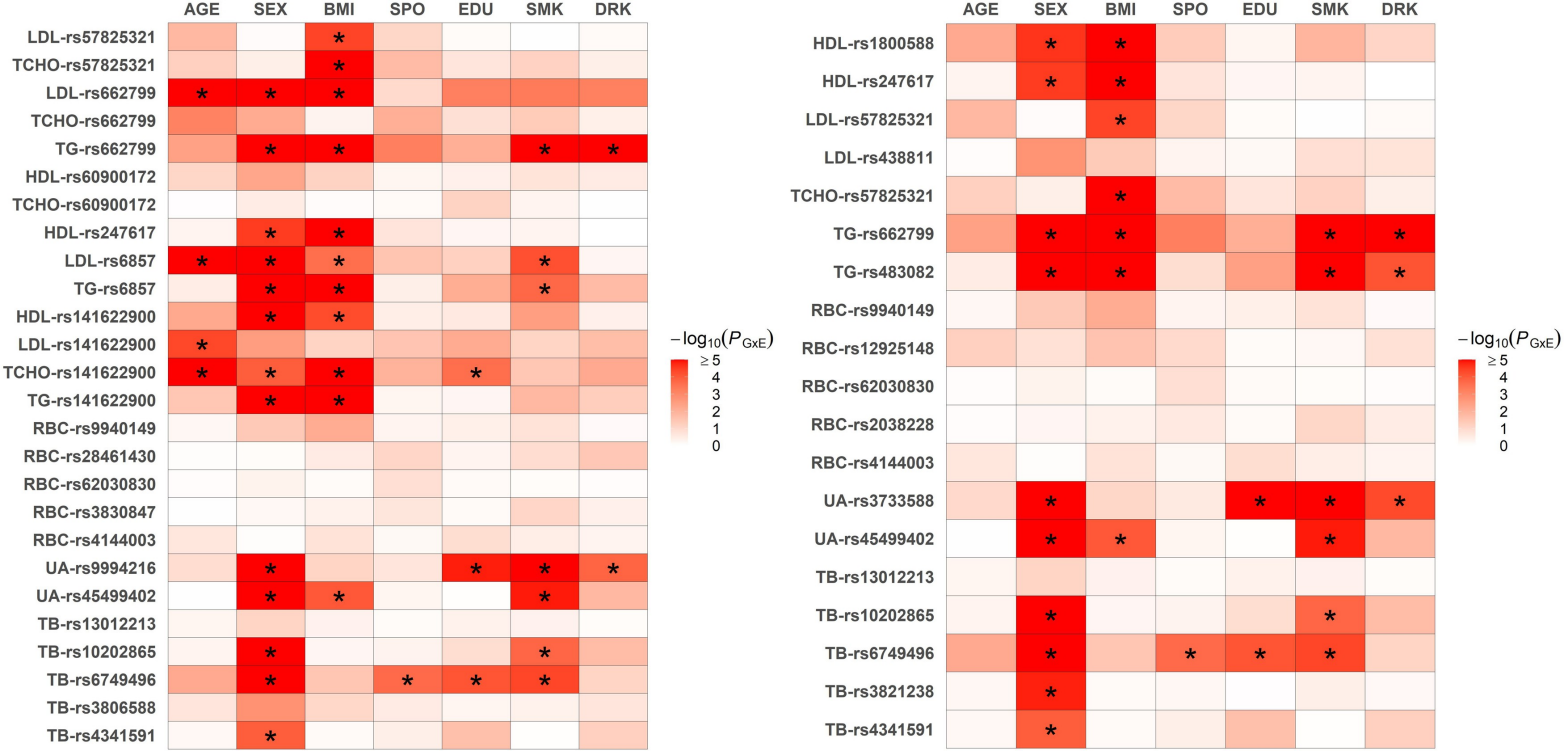
Heatmap plots of the GxE test *P*-values of the 26 (left figure, MST) and 19 (right figure, UST) trait-vQTL combinations. The left vertical axis labels the trait-vQTL combinations, whereas the top horizontal axis lists the environmental factors (SPO, regular exercise; EDU, educational attainment; SMK, smoking status; DRK, drinking status). The color in each cell marks the strength of −log_10_(GxE test *P*-value). In the left column (MST), “*” denotes that the GxE effect is significant at *P* < .05⁄(26 × 7) = .00027, where 26 is the number of trait-vQTL combinations in MST, and 7 is the number of environmental factors. In the right column (UST), “*” denotes that the GxE effect is significant at *P* < .05⁄(19 × 7) = .00038, where 19 is the number of trait-vQTL combinations in UST, and 7 is the number of environmental factors. A total of 41 “*” from the left figure (MST) and 29 “*” from the right figure (UST)

From Table 1, I picked 26 trait-vQTL combinations identified by MST and the *ad hoc* analysis (with UST *P*-value <.05/65 = .00077 in TWB2 or TWB1, where 65 is the number of tests under MST’s *ad hoc* analysis). Subsequently, I merged the TWB1 and TWB2 cohorts to investigate which E enriched the GxE signal. I performed a direct GxE test based on an additive genetic model with an interaction term between the vQTL SNP and one of seven Es (the top horizontal axis of Fig. 5). The left column of Fig. 5 presents the heatmap plot of the GxE test *P*-values of these 26 trait-vQTL combinations. A total of 41 significant GxE effects (41 *) were identified with *P *<* *.05⁄(26 × 7) = .00027. All Es enriched the GxE signals with some vQTLs (SEX 13 *, BMI 11 *, smoking [SMK] 7 *, AGE 4 *, education [EDU] 3 *, drinking [DRK] 2 *, and regular exercise [SPO] 1 *).

To compare, the right column of Fig. 5 shows the parallel analysis for the 19 trait-vQTL combinations identified by UST (19 yellow cells in Supplementary Table S5). Because rs57825321 in the *APOB* gene was vQTL of both LDL and TCHO, it generated two trait-vQTL combinations: LDL–rs57825321 and TCHO–rs57825321 (right column of Fig. 5). A total of 29 significant GxE effects (29 *) were identified with *P *<* *.05⁄(19 × 7) = .00038. As a result, MST explored more GxE effects than UST (41 > 29), although MST’s GxE significance level was even more stringent (.00027 < .00038).

I also investigated whether combining the TWB1 and TWB2 cohorts in a GxE analysis was reasonable. Supplementary Fig. S65 presents the scatter plots of the GxE effect sizes from the discovery (TWB2) and replication (TWB1) cohorts, for the 41 and 29 significant GxE effects (Fig. 5, 41 * from MST and 29 * from UST), respectively. The GxE effect sizes from the two cohorts were very similar, with Pearson’s correlation coefficients = 0.977 (MST’s 41 pairs of GxE effect sizes) and 0.986 (UST’s 29 pairs of GxE effect sizes), respectively. Therefore, combining TWB1 and TWB2 in one GxE analysis is justifiable.

## 4 Discussion

GxE has received much attention because this topic is related to how lifestyle factors modify the effects of hereditary materials (Ottman 1996). The concept of MST is similar to the strategy of analysis of variance (ANOVA). I first tested whether GxE exists in a group of phenotypes. If yes, I continued searching for the phenotype(s) that account(s) for GxE. Like the comparison between ANOVA and the two-sample *t*-test, MST can gain more power than UST because of (1) integrating more traits with GxE information and (2) the less harsh penalty on multiple testing. However, if only few traits account for GxE, MST can lose power due to aggregating more non-informative traits into the test statistic (Fig. 1).

Using the median-based Levene’s test, Westerman *et al.* recently searched genome-wide vQTLs for 20 cardiometabolic traits and identified 136 vQTLs from the UKB (Westerman *et al.* 2022). Although investigating vQTLs for individual traits separately, Westerman *et al.* observed “pleiotropy” (regarding phenotypic variance) in many loci. For example, in line with Table 1, *APOC1* was detected as a vQTL of all four lipid traits (HDL, LDL, TCHO, and TG). Westerman *et al.*’s findings justified the approach to gathering multiple continuous traits in vQTL identification.

The significant GxE effects identified in Fig. 5 are consistent with several previous studies (Yin *et al.* 2011, Lin *et al.* 2022, Wang *et al.* 2022). For example, data from 393 healthy US adults showed that the genetic impact of *APOA5* on LDL levels was sex-dependent (Wang *et al.* 2022), which was in line with the sex-*APOA5 rs662799* interaction on LDL (solely detected by MST, Fig. 5). Data from 1030 unrelated Chinese (Yin *et al.* 2011) demonstrated drinking–*APOA5* interaction on TG, consistent with the finding of MST and UST (Fig. 5). A Chinese community-based cohort including 4329 adults showed that BMI significantly modulated the *APOA5 rs662799* genetic effects on dyslipidemia risk (Lin *et al.* 2022), in line with the BMI–*APOA5 rs662799* interaction on TG (Fig. 5).

No matter whether MST or UST was applied, I detected no vQTLs from the TWB data for the six lung function traits (MST: Fig. 4A; UST: Supplementary Fig. S62A–F), five obesity traits (MST: Fig. 4C; UST: Supplementary Fig. S63A–E), two blood pressure traits (MST: Fig. 4G; UST: Supplementary Fig. S64F–G), or two diabetes traits (MST: Fig. 4H; UST: Supplementary Fig. S64H–I). Wang *et al.* (2019) also found no vQTLs from UKB’s lung function traits such as FVC and FEV1. Further investigation can be performed on the FEV1/FVC ratio (FFR). Wang *et al.* (2019) identified three vQTLs for FFR and GxE between the *CHRNA5-A3-B4* locus and smoking in their Supplementary Table S3(a).

In line with the results of this study, a previous genome-wide vQTL analysis from the UKB data suggested minor GxE effects in diastolic and systolic blood pressure levels (Shi 2022). Although UKB data demonstrated some vQTLs from obesity traits such as BMI, WC, and HC (Wang *et al.* 2019, Miao *et al.* 2022), TWB data showed no obesity vQTLs either based on MST (Fig. 4C) or UST (Supplementary Fig. S63A–E). This inconsistency may result from the difference in ethnicity [European ancestry (Wang *et al.* 2019, Miao *et al.* 2022) versus Taiwan’s Han Chinese (this study)] or sample size [∼350 000 (Wang *et al.* 2019, Miao *et al.* 2022,) versus ∼118 900 (this study)]. To sum up, this study provided an MST approach to detect vQTLs from multiple continuous traits. Moreover, 18 independent vQTLs for Taiwan’s Han Chinese continuous traits were identified and replicated (Table 1), and 41 GxE effects were subsequently explored (Fig. 5, left column). MST identified 12 additional GxE signals than UST (41 > 29) (Fig. 5).

## Supplementary Material

btae419_Supplementary_Data

## Data Availability

The data underlying this article were provided by the Taiwan Biobank. Data will be shared on request to the corresponding author with permission from the Taiwan Biobank.
