# Supplementary material for: Detecting gene–environment interactions from multiple continuous traits
Source: Bioinformatics. 2024 Jun 25;40(7):btae419. doi: 10.1093/bioinformatics/btae419 (PMC11254352; doi:10.1093/bioinformatics/btae419)
Supplement: btae419_Supplementary_Data [file btae419_supplementary_data.pdf]

Supplementary data of

Detecting gene-environment interactions from multiple continuous traits

Short title: Gene-environment interactions

Author

Wan-Yu Lin,[1, 2] *

Affiliations

[1] Institute of Health Data Analytics and Statistics, College of Public Health, National Taiwan University, Taipei, Taiwan

[2] Master of Public Health Degree Program, College of Public Health, National Taiwan University, Taipei, Taiwan

* Corresponding author: Wan-Yu Lin, Ph.D.

Wan-Yu Lin, Ph.D.

Room 501, No. 17, Xu-Zhou Road, Taipei 100, Taiwan

Phone/Fax: +886-2-33668106;   E-mail: linwy@ntu.edu.tw

https://orcid.org/0000-0002-3385-4702

Contents

Table S1. MST's false discovery rates (FDR) of testing GxE for individual traits, when GxE influenced the first trait (*n* = 93,708, exposure prevalence = 0.2) 9

Table S2. MST's false discovery rates (FDR) of testing GxE for individual traits, when GxE influenced the first and the second traits (*n* = 93,708, exposure prevalence = 0.2) 10

Table S3. Average memory usage and time consumption of MST and UST (simulation data of exposure prevalence = 0.2 & 0.5 were combined together) 11

Table S4. Skewness and excess kurtosis of the 29 Taiwan Biobank continuous traits (traits have been adjusted for genotypes and covariates) 13

Table S5. The 18 variance quantitative trait loci detected by UST (only 17 independent loci; *APOC1* rs483082 [vQTL of TG] and *APOC1* rs438811 [vQTL of LDL] were highly correlated with r^2 = 0.99). 15

Figure S1. The quantile-quantile (QQ) plots stratified by the nine ranges of MAFs (three traits from a multivariate normal distribution, exposure prevalence = 0.2, and *n* = 93,708). 16

Figure S2. The quantile-quantile (QQ) plots stratified by the nine ranges of MAFs (three traits, each from a Chi-squared distribution with the degree of freedom 1, exposure prevalence = 0.2, and *n* = 93,708). 17

Figure S3. The quantile-quantile (QQ) plots stratified by the nine ranges of MAFs (three traits from a multivariate normal distribution, exposure prevalence = 0.2, and *n* = 25,200). 18

Figure S4. The quantile-quantile (QQ) plots stratified by the nine ranges of MAFs (three traits, each from a Chi-squared distribution with the degree of freedom 1, exposure prevalence = 0.2, and *n* = 25,200). 19

Figure S5. The quantile-quantile (QQ) plots stratified by the nine ranges of MAFs (four traits from a multivariate normal distribution, exposure prevalence = 0.2, and *n* = 93,708). 20

Figure S6. The quantile-quantile (QQ) plots stratified by the nine ranges of MAFs (four traits, each from a Chi-squared distribution with the degree of freedom 1, exposure prevalence = 0.2, and *n* = 93,708). 21

Figure S7. The quantile-quantile (QQ) plots stratified by the nine ranges of MAFs (four traits from a multivariate normal distribution, exposure prevalence = 0.2, and *n* = 25,200). 22

Figure S8. The quantile-quantile (QQ) plots stratified by the nine ranges of MAFs (four traits, each from a Chi-squared distribution with the degree of freedom 1, exposure prevalence = 0.2, and *n* = 25,200). 23

Figure S9. Power and false discovery rate (FDR) of testing GxE for individual traits, when GxE influenced the first trait (three traits, each from a Chi-squared distribution with the degree of freedom 1, exposure prevalence = 0.2, and *n* = 93,708). 24

Figure S10. Power and false discovery rate (FDR) of testing GxE for individual traits, when GxE influenced the first and the second traits (three traits, each from a Chi-squared distribution with the degree of freedom 1, exposure prevalence = 0.2, and *n* = 93,708). 25

Figure S11. Power of testing GxE for individual traits, when GxE influenced all three traits (three traits, each from a Chi-squared distribution with the degree of freedom 1, exposure prevalence = 0.2, and *n* = 93,708). 26

Figure S12. Power and false discovery rate (FDR) of testing GxE for individual traits, when GxE influenced the first trait (three traits from a multivariate normal distribution, exposure prevalence = 0.5, and *n* = 93,708). 27

Figure S13. Power and false discovery rate (FDR) of testing GxE for individual traits, when GxE influenced the first trait (three traits, each from a Chi-squared distribution with the degree of freedom 1, exposure prevalence = 0.5, and *n* = 93,708). 27

Figure S14. Power and false discovery rate (FDR) of testing GxE for individual traits, when GxE influenced the first and the second traits (three traits from a multivariate normal distribution, exposure prevalence = 0.5, and *n* = 93,708). 28

Figure S15. Power and false discovery rate (FDR) of testing GxE for individual traits, when GxE influenced the first and the second traits (three traits, each from a Chi-squared distribution with the degree of freedom 1, exposure prevalence = 0.5, and *n* = 93,708). 28

Figure S16. Power of testing GxE for individual traits, when GxE influenced all three traits (three traits from a multivariate normal distribution, exposure prevalence = 0.5, and *n* = 93,708). 29

Figure S17. Power of testing GxE for individual traits, when GxE influenced all three traits (three traits, each from a Chi-squared distribution with the degree of freedom 1, exposure prevalence = 0.5, and *n* = 93,708). 29

Figure S18. Power and false discovery rate (FDR) of testing GxE for individual traits, when GxE influenced the first trait (three traits from a multivariate normal distribution, exposure prevalence = 0.2, and *n* = 25,200). Some FDR levels were missing values due to no discoveries. 30

Figure S19. Power and false discovery rate (FDR) of testing GxE for individual traits, when GxE influenced the first trait (three traits, each from a Chi-squared distribution with the degree of freedom 1, exposure prevalence = 0.2, and *n* = 25,200). Some FDR levels were missing values due to no discoveries. 30

Figure S20. Power and false discovery rate (FDR) of testing GxE for individual traits, when GxE influenced the first and the second traits (three traits from a multivariate normal distribution, exposure prevalence = 0.2, and *n* = 25,200). Some FDR levels were missing values due to no discoveries. 31

Figure S21. Power and false discovery rate (FDR) of testing GxE for individual traits, when GxE influenced the first and the second traits (three traits, each from a Chi-squared distribution with the degree of freedom 1, exposure prevalence = 0.2, and *n* = 25,200). Some FDR levels were missing values due to no discoveries. 31

Figure S22. Power of testing GxE for individual traits, when GxE influenced all three traits (three traits from a multivariate normal distribution, exposure prevalence = 0.2, and *n* = 25,200). 32

Figure S23. Power of testing GxE for individual traits, when GxE influenced all three traits (three traits, each from a Chi-squared distribution with the degree of freedom 1, exposure prevalence = 0.2, and *n* = 25,200). 32

Figure S24. Power and false discovery rate (FDR) of testing GxE for individual traits, when GxE influenced the first trait (three traits from a multivariate normal distribution, exposure prevalence = 0.5, and *n* = 25,200). Some FDR levels were missing values due to no discoveries. 33

Figure S25. Power and false discovery rate (FDR) of testing GxE for individual traits, when GxE influenced the first trait (three traits, each from a Chi-squared distribution with the degree of freedom 1, exposure prevalence = 0.5, and *n* = 25,200). Some FDR levels were missing values due to no discoveries. 33

Figure S26. Power and false discovery rate (FDR) of testing GxE for individual traits, when GxE influenced the first and the second traits (three traits from a multivariate normal distribution, exposure prevalence = 0.5, and *n* = 25,200). 34

Figure S27. Power and false discovery rate (FDR) of testing GxE for individual traits, when GxE influenced the first and the second traits (three traits, each from a Chi-squared distribution with the degree of freedom 1, exposure prevalence = 0.5, and *n* = 25,200). 34

Figure S28. Power of testing GxE for individual traits, when GxE influenced all three traits (three traits from a multivariate normal distribution, exposure prevalence = 0.5, and *n* = 25,200). 35

Figure S29. Power of testing GxE for individual traits, when GxE influenced all three traits (three traits, each from a Chi-squared distribution with the degree of freedom 1, exposure prevalence = 0.5, and *n* = 25,200). 35

Figure S30. Power and false discovery rate (FDR) of testing GxE for individual traits, when GxE influenced the first trait (four traits from a multivariate normal distribution, exposure prevalence = 0.2, and *n* = 93,708). Each point was calculated based on 10,000 replications. The title of each plot represents $\boldsymbol{\rho 12}, \boldsymbol{\rho 13}, \boldsymbol{\rho 14}, \boldsymbol{\rho 23}, \boldsymbol{\rho 24}, \boldsymbol{\rho 34}$, where $\boldsymbol{\rho uv}$ denotes the correlation between the uth and the vth traits, and u, v $\in \mathbf{1}, \mathbf{2}, \mathbf{3}, \mathbf{4}$. 36

Figure S31. Power and false discovery rate (FDR) of testing GxE for individual traits, when GxE influenced the first trait (four traits, each from a Chi-squared distribution with the degree of freedom 1, exposure prevalence = 0.2, and *n* = 93,708). Each point was calculated based on 10,000 replications. The title of each plot represents $\boldsymbol{\rho 12}, \boldsymbol{\rho 13}, \boldsymbol{\rho 14}, \boldsymbol{\rho 23}, \boldsymbol{\rho 24}, \boldsymbol{\rho 34}$, where $\boldsymbol{\rho uv}$ denotes the correlation between the uth and the vth traits, and u, v $\in \mathbf{1}, \mathbf{2}, \mathbf{3}, \mathbf{4}$. 37

Figure S32. Power and false discovery rate (FDR) of testing GxE for individual traits, when GxE influenced the first and the second traits (four traits from a multivariate normal distribution, exposure prevalence = 0.2, and *n* = 93,708). 38

Figure S33. Power and false discovery rate (FDR) of testing GxE for individual traits, when GxE influenced the first and the second traits (four traits, each from a Chi-squared distribution with the degree of freedom 1, exposure prevalence = 0.2, and *n* = 93,708). 38

Figure S34. Power and false discovery rate (FDR) of testing GxE for individual traits, when GxE influenced the first three traits (four traits from a multivariate normal distribution, exposure prevalence = 0.2, and *n* = 93,708). 39

Figure S35. Power and false discovery rate (FDR) of testing GxE for individual traits, when GxE influenced the first three traits (four traits, each from a Chi-squared distribution with the degree of freedom 1, exposure prevalence = 0.2, and *n* = 93,708). 39

Figure S36. Power of testing GxE for individual traits, when GxE influenced all four traits (four traits from a multivariate normal distribution, exposure prevalence = 0.2, and *n* = 93,708). 40

Figure S37. Power of testing GxE for individual traits, when GxE influenced all four traits (four traits, each from a Chi-squared distribution with the degree of freedom 1, exposure prevalence = 0.2, and *n* = 93,708). 40

Figure S38. Power and false discovery rate (FDR) of testing GxE for individual traits, when GxE influenced the first trait (four traits from a multivariate normal distribution, exposure prevalence = 0.5, and *n* = 93,708). 41

Figure S39. Power and false discovery rate (FDR) of testing GxE for individual traits, when GxE influenced the first trait (four traits, each from a Chi-squared distribution with the degree of freedom 1, exposure prevalence = 0.5, and *n* = 93,708). 41

Figure S40. Power and false discovery rate (FDR) of testing GxE for individual traits, when GxE influenced the first and the second traits (four traits from a multivariate normal distribution, exposure prevalence = 0.5, and *n* = 93,708). 42

Figure S41. Power and false discovery rate (FDR) of testing GxE for individual traits, when GxE influenced the first and the second traits (four traits, each from a Chi-squared distribution with the degree of freedom 1, exposure prevalence = 0.5, and *n* = 93,708). 42

Figure S42. Power and false discovery rate (FDR) of testing GxE for individual traits, when GxE influenced the first three traits (four traits from a multivariate normal distribution, exposure prevalence = 0.5, and *n* = 93,708). 43

Figure S43. Power and false discovery rate (FDR) of testing GxE for individual traits, when GxE influenced the first three traits (four traits, each from a Chi-squared distribution with the degree of freedom 1, exposure prevalence = 0.5, and *n* = 93,708). 43

Figure S44. Power of testing GxE for individual traits, when GxE influenced all four traits (four traits from a multivariate normal distribution, exposure prevalence = 0.5, and *n* = 93,708). 44

Figure S45. Power of testing GxE for individual traits, when GxE influenced all four traits (four traits, each from a Chi-squared distribution with the degree of freedom 1, exposure prevalence = 0.5, and *n* = 93,708). 44

Figure S46. Power and false discovery rate (FDR) of testing GxE for individual traits, when GxE influenced the first trait (four traits from a multivariate normal distribution, exposure prevalence = 0.2, and *n* = 25,200). Some FDR levels were missing values due to no discoveries. 45

Figure S47. Power and false discovery rate (FDR) of testing GxE for individual traits, when GxE influenced the first trait (four traits, each from a Chi-squared distribution with the degree of freedom 1, exposure prevalence = 0.2, and *n* = 25,200). Some FDR levels were missing values due to no discoveries. Low power = > very few discoveries => FDR changed dramatically. 45

Figure S48. Power and false discovery rate (FDR) of testing GxE for individual traits, when GxE influenced the first and the second traits (four traits from a multivariate normal distribution, exposure prevalence = 0.2, and *n* = 25,200). 46

Figure S49. Power and false discovery rate (FDR) of testing GxE for individual traits, when GxE influenced the first and the second traits (four traits, each from a Chi-squared distribution with the degree of freedom 1, exposure prevalence = 0.2, and *n* = 25,200). Some FDR levels were missing values due to no discoveries. 46

Figure S50. Power and false discovery rate (FDR) of testing GxE for individual traits, when GxE influenced the first three traits (four traits from a multivariate normal distribution, exposure prevalence = 0.2, and *n* = 25,200). 47

Figure S51. Power and false discovery rate (FDR) of testing GxE for individual traits, when GxE influenced the first three traits (four traits, each from a Chi-squared distribution with the degree of freedom 1, exposure prevalence = 0.2, and *n* = 25,200). Some FDR levels were missing values due to no discoveries. 47

Figure S52. Power of testing GxE for individual traits, when GxE influenced all four traits (four traits from a multivariate normal distribution, exposure prevalence = 0.2, and *n* = 25,200). 48

Figure S53. Power of testing GxE for individual traits, when GxE influenced all four traits (four traits, each from a Chi-squared distribution with the degree of freedom 1, exposure prevalence = 0.2, and *n* = 25,200). 48

Figure S54. Power and false discovery rate (FDR) of testing GxE for individual traits, when GxE influenced the first trait (four traits from a multivariate normal distribution, exposure prevalence = 0.5, and *n* = 25,200). Some FDR levels were missing values due to no discoveries. 49

Figure S55. Power and false discovery rate (FDR) of testing GxE for individual traits, when GxE influenced the first trait (four traits, each from a Chi-squared distribution with the degree of freedom 1, exposure prevalence = 0.5, and *n* = 25,200). Some FDR levels were missing values due to no discoveries. 49

Figure S56. Power and false discovery rate (FDR) of testing GxE for individual traits, when GxE influenced the first and the second traits (four traits from a multivariate normal distribution, exposure prevalence = 0.5, and *n* = 25,200). 50

Figure S57. Power and false discovery rate (FDR) of testing GxE for individual traits, when GxE influenced the first and the second traits (four traits, each from a Chi-squared distribution with the degree of freedom 1, exposure prevalence = 0.5, and *n* = 25,200). 50

Figure S58. Power and false discovery rate (FDR) of testing GxE for individual traits, when GxE influenced the first three traits (four traits from a multivariate normal distribution, exposure prevalence = 0.5, and *n* = 25,200). 51

Figure S59. Power and false discovery rate (FDR) of testing GxE for individual traits, when GxE influenced the first three traits (four traits, each from a Chi-squared distribution with the degree of freedom 1, exposure prevalence = 0.5, and *n* = 25,200). 51

Figure S60. Power of testing GxE for individual traits, when GxE influenced all four traits (four traits from a multivariate normal distribution, exposure prevalence = 0.5, and *n* = 25,200). 52

Figure S61. Power of testing GxE for individual traits, when GxE influenced all four traits (four traits, each from a Chi-squared distribution with the degree of freedom 1, exposure prevalence = 0.5, and *n* = 25,200). 52

Figure S62. The Manhattan plots of the UST analysis on 6 TWB2 lung function traits and 4 TWB2 lipid traits 53

Figure S63. The Manhattan plots of the UST analysis on 5 TWB2 obesity traits and 5 TWB2 blood traits 54

Figure S64. The Manhattan plots of the UST analysis on 3 TWB2 kidney traits, 2 liver traits, 2 hypertension traits, and 2 diabetes traits........ 55

Figure S65. The scatter plots of the GxE effect sizes from the discovery (TWB2) and replication (TWB1) cohorts 56

	CORRELATION SCENARIO							
**MAF RANGE OF SNPS**	(A)	(B)	(C)	(D)	(E)	(F)	(G)	(H)
**[0.05, 0.10)**	0.0000	0.0286	0.0400	0.0000	0.0476	0.0058	0.0000	0.0303
**[0.10, 0.15)**	0.0171	0.0037	0.0167	0.0107	0.0053	0.0028	0.0145	0.0145
**[0.15, 0.20)**	0.0093	0.0072	0.0039	0.0048	0.0049	0.0032	0.0056	0.0048
**[0.20, 0.25)**	0.0035	0.0050	0.0055	0.0033	0.0025	0.0016	0.0037	0.0051
**[0.25, 0.30)**	0.0035	0.0023	0.0029	0.0010	0.0022	0.0024	0.0038	0.0034
**[0.30, 0.35)**	0.0027	0.0009	0.0019	0.0016	0.0021	0.0017	0.0020	0.0022
**[0.35, 0.40)**	0.0020	0.0012	0.0021	0.0023	0.0015	0.0015	0.0018	0.0012
**[0.40, 0.45)**	0.0008	0.0019	0.0014	0.0021	0.0017	0.0025	0.0010	0.0024
**[0.45, 0.50]**	0.0013	0.0016	0.0024	0.0017	0.0015	0.0011	0.0013	0.0020

Table S1. **MST's false discovery rates (FDR) of testing GxE for individual traits, when GxE influenced the first trait (*n* = 93,708, exposure prevalence = 0.2)**

Note: UST's FDR of testing GxE for individual traits were all 0.

	CORRELATION SCENARIO							
**MAF RANGE OF SNPS**	(A)	(B)	(C)	(D)	(E)	(F)	(G)	(H)
**[0.05, 0.10)**	0.0022	0.0015	0.0028	0.0011	0.0007	0.0022	0.0000	0.0006
**[0.10, 0.15)**	0.0012	0.0006	0.0002	0.0007	0.0010	0.0007	0.0004	0.0010
**[0.15, 0.20)**	0.0006	0.0002	0.0005	0.0003	0.0006	0.0005	0.0005	0.0007
**[0.20, 0.25)**	0.0003	0.0002	0.0005	0.0004	0.0003	0.0005	0.0006	0.0005
**[0.25, 0.30)**	0.0004	0.0004	0.0003	0.0005	0.0003	0.0005	0.0006	0.0006
**[0.30, 0.35)**	0.0004	0.0003	0.0002	0.0004	0.0004	0.0001	0.0006	0.0004
**[0.35, 0.40)**	0.0005	0.0004	0.0004	0.0002	0.0004	0.0003	0.0004	0.0004
**[0.40, 0.45)**	0.0004	0.0005	0.0005	0.0004	0.0003	0.0007	0.0004	0.0004
**[0.45, 0.50]**	0.0002	0.0002	0.0002	0.0004	0.0005	0.0004	0.0003	0.0007

Table S2. **MST's false discovery rates (FDR) of testing GxE for individual traits, when GxE influenced the first and the second traits (*n* = 93,708, exposure prevalence = 0.2)**

Note: UST's FDR of testing GxE for individual traits were all 0.

			Memory usage		Time consumption [1]	
**Number of traits**	Sample size	Trait distribution	MST	UST	MST	UST
**3**	93,708	Normal	269.79 Mb	332.11 Mb	0.072 second	0.093 second
		Chi-squared distribution with the degree of freedom 1	406.86 Mb	464.80 Mb	0.067 second	0.080 second
	25,200	Normal	250.85 Mb	257.20 Mb	0.020 second	0.026 second
		Chi-squared distribution with the degree of freedom 1	283.57 Mb	288.61 Mb	0.023 second	0.027 second
**4**	93,708	Normal	320.55 Mb	354.69 Mb	0.081 second	0.125 second
		Chi-squared distribution with the degree of freedom 1	424.79 Mb	479.24 Mb	0.081 second	0.121 second
	25,200	Normal	286.27 Mb	294.77 Mb	0.024 second	0.034 second
		Chi-squared distribution with the degree of freedom 1	287.04 Mb	296.67 Mb	0.024 second	0.036 second

Table S3. **Average memory usage and time consumption of MST and UST (simulation data of exposure prevalence = 0.2 & 0.5 were combined together)**

[1] The average execution times of MST and UST were measured in R (version 4.2.3) on a Windows system running at 3.40 GHz and 64 GB of RAM.

	Skewness (TWB2 / TWB1)	Excess kurtosis = kurtosis - 3 (TWB2 / TWB1)
**Normal distribution**	0	0
**Chi-squared distribution with the degree of freedom 1**	$\sqrt{8}$=2.8	12
**Data considered normally distributed [1, 2]**	Between -2 to +2	Between -7 to +7
	**6 lung function traits**	
**vital capacity**	0.12 / 0.36	0.98 / 1.50
**tidal volume**	0.98 / 1.04	1.56 / 1.46
**inspiratory reserve volume**	0.20 / 0.20	0.47 / 0.39
**expiratory reserve volume**	0.63 / 0.66	0.81 / 0.93
**forced vital capacity (FVC)**	0.03 / 0.23	1.09 / 1.64
**forced expiratory volume in 1 second (FEV1)**	-0.72 / -0.45	1.24 / 0.42
	**4 lipid traits**	
**high-density lipoprotein cholesterol (HDL)**	0.59 / 0.64	0.77 / 1.00
**low-density lipoprotein cholesterol (LDL)**	0.37 / 0.38	0.55 / 0.59
**total cholesterol (TCHO)**	0.43 / 0.43	0.63 / 0.57
**triglyceride (TG)**	2.02 / 1.91	6.93 / 6.20
	**5 obesity traits**	
**body mass index (BMI)**	0.88 / 0.78	1.48 / 1.28
**body fat percentage (BFP)**	0.28 / 0.33	0.73 / 0.89
**waist circumference (WC)**	0.60 / 0.56	0.93 / 0.89
**hip circumference (HC)**	0.69 / 0.60	1.30 / 1.08
**waist-hip ratio (WHR)**	0.21 / 0.21	0.24 / 0.37
	**5 blood traits**	
**red blood cells (RBC)**	0.94 / 0.93	2.68 / 2.76
**white blood cells (WBC)**	0.62 / 0.66	1.07 / 1.10
**platelets**	0.53 / 0.54	1.27 / 1.19
**hemoglobin (HB)**	-0.90 / -0.79	2.15 / 1.93
**hematocrit (HCT)**	-0.48 / -0.21	1.54 / 1.11
	**3 kidney traits**	
**Creatinine**	1.19 / 1.45	6.45 / 8.72
**uric acid (UA)**	0.34 / 0.34	1.04 / 1.17

**blood urea nitrogen**	0.73 / 0.74	1.30 / 1.41
**2 liver traits**		
**total bilirubin (TB)**	1.24 / 1.27	2.69 / 2.88
**albumin**	-0.18 / -0.12	0.57 / 0.64
**2 hypertension traits**		
**diastolic blood pressure**	0.53 / 0.43	0.75 / 0.60
**systolic blood pressure**	0.72 / 0.64	1.20 / 1.02
**2 diabetes traits**		
**fasting glucose (FG)**	2.80 / 2.87	13.73 / 14.07
**glycated hemoglobin (HbA1c)**	2.28 / 2.29	10.38 / 10.24

Table S4. **Skewness and excess kurtosis of the 29 Taiwan Biobank continuous traits (traits have been adjusted for genotypes and covariates)**

[1] Byrne, B. M. (2010). Structural equation modeling with AMOS: Basic concepts, applications, and programming. New York: Routledge.

[2] Hair, J., Black, W. C., Babin, B. J. & Anderson, R. E. (2010) Multivariate data analysis (7th ed.). Upper Saddle River, New Jersey: Pearson Educational International.

Four lipids traits										
**Chr.**	**BP**	**SNP**	**MAF (TWB2 / TWB1)**	**Gene**	**MST *p* (TWB2 / TWB1)** [1]	**HDL UST *p*** [2]	**LDL UST *p*** [2]	**TCHO UST *p*** [2]	**TG UST *p*** [2]	
2	21024193	rs57825321	0.148 / 0.146	*APOB*	**3.3E-22 / 1.0E-5**	6.8E-3 / 0.89	**6.7E-25 / 1.8E-7**	**9.3E-16 / 3.8E-6**	0.03 / 0.03	
11	116792991	rs662799	0.274 / 0.275	*APOA5*	**7.8E-215 / 1.9E-70**	0.01 / 0.09	5.5E-12 / 1.9E-3	1.7E-12 / 2.3E-3	**9.9E-213 / 4.0E-73**	
15	58431476	rs1800588	0.359 / 0.358	*LIPC*	1.3E-14 / 2.3E-4	**9.1E-12 / 1.2E-5**	0.96 / 0.55	1.6E-3 / 0.06	3.6E-3 / 0.71	
16	56956804	rs247617	0.161 / 0.160	*CETP*	**6.3E-44 / 1.6E-7**	**2.7E-48 / 1.6E-8**	0.32 / 0.20	0.08 / 0.18	0.15 / 0.05	
19	44912921	rs483082	0.175 / 0.173	*APOC1*	**1.1E-114 / 3.2E-33**	6.1E-5 / 0.09	**7.1E-60 / 7.3E-15**	5.0E-19 / 6.7E-3	**3.2E-27 / 4.2E-8** [3]	
19	44913484	rs438811	0.175 / 0.173	*APOC1*	**9.4E-115 / 2.2E-33**	5.5E-5 / 0.09	**6.8E-60 / 6.6E-15** [4]	5.2E-19 / 6.7E-3	**3.4E-27 / 4.2E-8**	
**Five blood traits**										
**Chr.**	**BP**	**SNP**	**MAF (TWB2 / TWB1)**	**Gene**	**MST *p* (TWB2 / TWB1)** [1]	**RBC UST *p*** [2]	**WBC UST *p*** [2]	**Platelet UST *p*** [2]	**HB UST *p*** [2]	**HCT UST *p*** [2]
16	250642	rs9940149	0.446 / 0.449	*FAM234A*	**3.4E-137 / 3.7E-39**	**3.3E-136 / 3.8E-44**	0.99 / 0.24	0.21 / 0.28	3.8E-3 / 6.4E-3	0.35 / 0.02
16	344906	rs12925148	0.115 / 0.115	*AXIN1*	2.3E-14 / 2.4E-4	**2.5E-18 / 6.4E-7**	0.78 / 0.38	0.81 / 0.61	0.08 / 0.83	0.23 / 0.91
16	418055	rs62030830	0.488 / 0.488	*DECR2*	**1.2E-57 / 9.9E-16**	**9.7E-62 / 1.6E-17**	0.57 / 0.39	0.80 / 0.47	0.10 / 0.31	0.67 / 0.21
16	489429	rs2038228	0.437 / 0.441	*RAB11FIP3*	**4.5E-38 / 1.1E-7**	**1.1E-39 / 6.2E-11**	0.09 / 0.58	0.85 / 0.68	0.02 / 0.65	0.67 / 0.58
16	595968	rs4144003	0.395 / 0.397	*RAB40C*	**3.5E-41 / 1.5E-10**	**6.0E-46 / 3.8E-12**	0.82 / 0.51	0.19 / 0.40	0.36 / 0.54	0.80 / 0.10
**Three kidney traits**										
**Chr.**	**BP**	**SNP**	**MAF**	**Gene**	**MST *p* (TWB2 /**	**Creatinine UST**	**UA UST *p*** [2]	**BUN UST *p*** [2]		

			(TWB2 / TWB1)		TWB1) [1]	*p* [2]				
4	9995679	rs3733588	0.415 / 0.418	*SLC2A9*	3.7E-24 / 5.8E-5	0.82 / 0.38	**1.6E-25 / 1.2E-5**	0.54 / 0.76		
4	88122482	rs45499402	0.314 / 0.319	*ABCG2*	**3.9E-62 / 3.4E-24**	0.05 / 0.12	**2.3E-62 / 3.9E-27**	0.08 / 0.68		
**Two liver traits**										
**Chr.**	**BP**	**SNP**	**MAF (TWB2 / TWB1)**	**Gene**	**MST *p* (TWB2 / TWB1)** [1]	**TB UST *p*** [2]	**Albumin UST *p*** [2]			
2	233347393	rs13012213	0.177 / 0.179	*SAG*	**7.9E-23 / 1.7E-5**	**8.7E-25 / 1.0E-6**	0.63 / 0.94			
2	233679061	rs10202865	0.189 / 0.188	*UGT1A10*	**4.6E-156 / 2.8E-52**	**1.3E-158 / 5.5E-54**	0.48 / 0.48			
2	233715640	rs6749496	0.114 / 0.112	*UGT1A10*	**0 / 7.9E-128**	**0 / 1.1E-130**	0.05 / 0.80			
2	233841134	rs3821238	0.275 / 0.280	*HJURP*	**4.9E-16 / 6.0E-6**	**3.3E-17 / 8.3E-7**	0.27 / 0.53			
12	20844459	rs4341591	0.157 / 0.157	*SLCO1B3*	**3.9E-21 / 1.6E-6**	**1.0E-22 / 2.0E-7**	0.95 / 0.48			

Table S5. **The 18 variance quantitative trait loci detected by UST (only 17 independent loci; *APOC1* rs483082 [vQTL of TG] and *APOC1* rs438811 [vQTL of LDL] were highly correlated with r^2 = 0.99).**

[1] MST *p*-values (TWB2 / TWB1) were highlighted in bold type if TWB2 MST $p < 2.4E-9$ and TWB1 MST $p < 0.05/1,767 = 2.8E-5$.

[2] UST *p*-values (TWB2 / TWB1) were highlighted in bold type if TWB2 UST $p < 6.7E-10$ and TWB1 UST $p < 0.05/1,904 = 2.6E-5$. UST *p*-values (TWB2 / TWB1) were highlighted in yellow if the vQTL was detected because of the trait. Further analysis for these 19 trait-vQTL combinations (corresponding to the 19 yellow cells in this table) is shown in the right column of Figure 5.

[3] rs483082 (in *APOC1*) was selected as the vQTL of TG. Although UST was also significant at rs438811 (in *APOC1*), it was not selected as the vQTL of TG because of the high LD with rs483082 (in *APOC1*) (r^2 = 0.99).

[4] rs438811 (in *APOC1*) was selected as the vQTL of LDL. Although UST was also significant at rs483082 (in *APOC1*), it was not selected as the vQTL of LDL because of the high LD with rs438811 (in *APOC1*) (r^2 = 0.99).

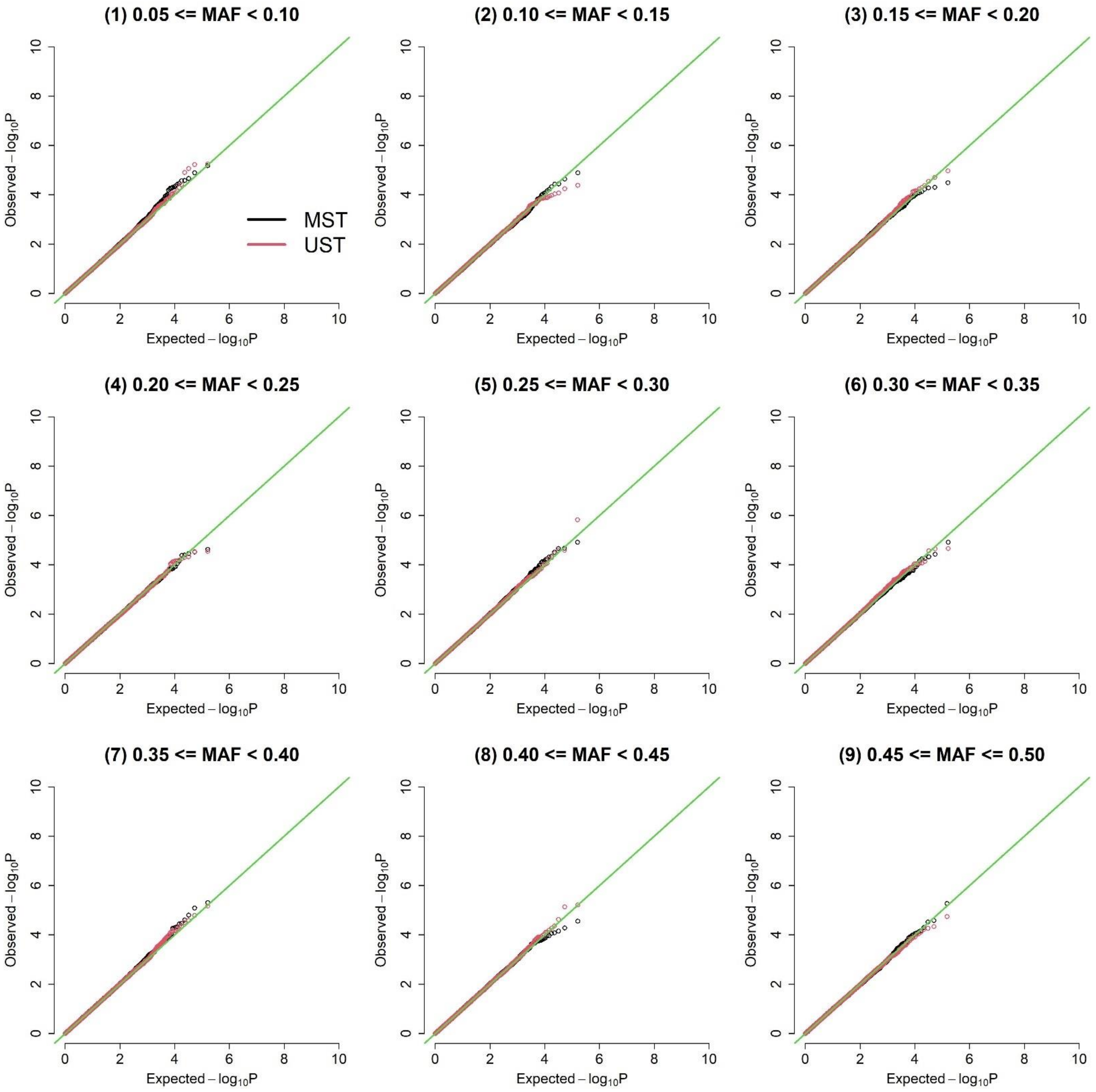

Figure S1. **The quantile-quantile (QQ) plots stratified by the nine ranges of MAFs (three traits from a multivariate normal distribution, exposure prevalence = 0.2, and *n* = 93,708).**

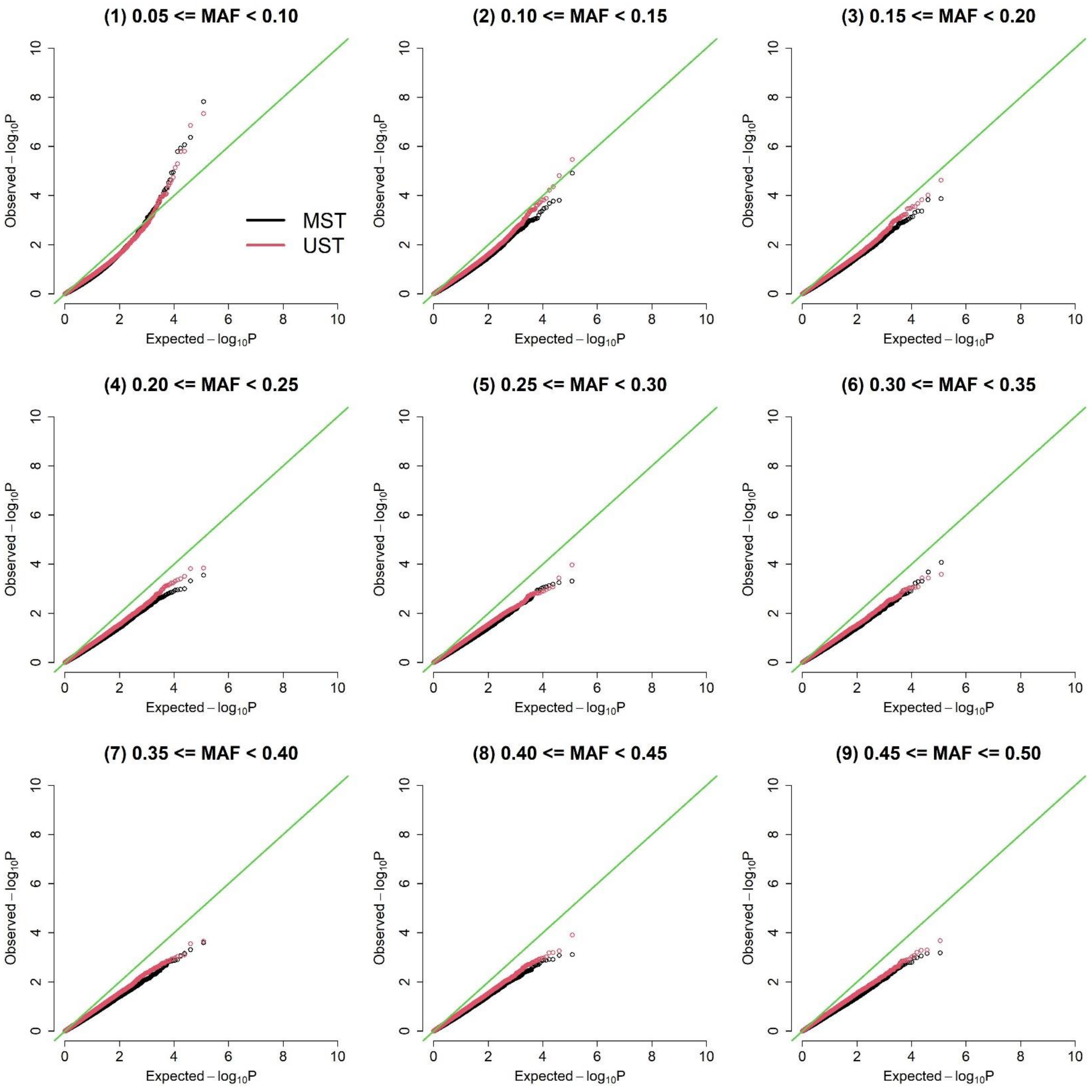

Figure S2. **The quantile-quantile (QQ) plots stratified by the nine ranges of MAFs (three traits, each from a Chi-squared distribution with the degree of freedom 1, exposure prevalence = 0.2, and *n* = 93,708).**

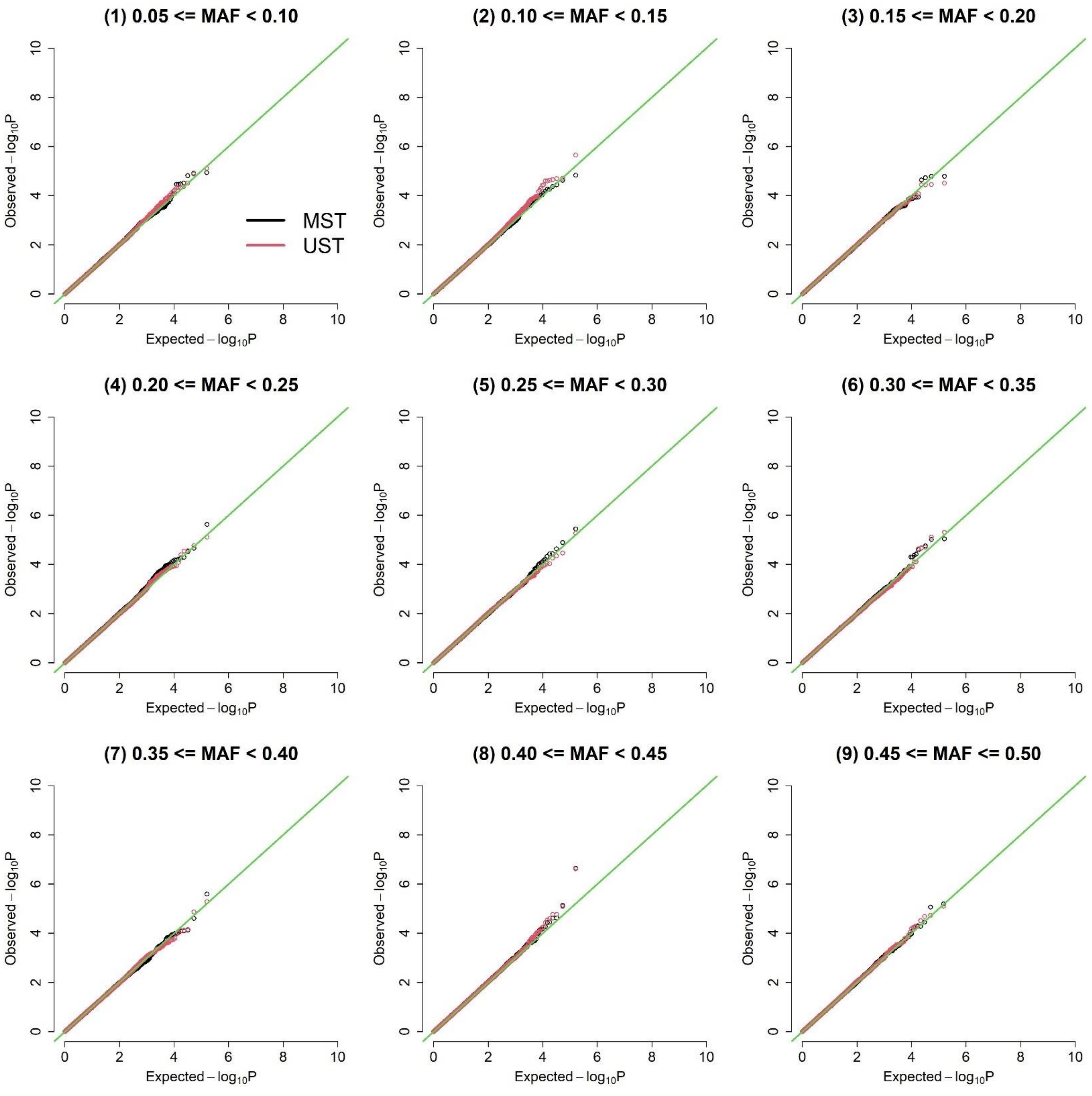

Figure S3. **The quantile-quantile (QQ) plots stratified by the nine ranges of MAFs (three traits from a multivariate normal distribution, exposure prevalence = 0.2, and *n* = 25,200).**

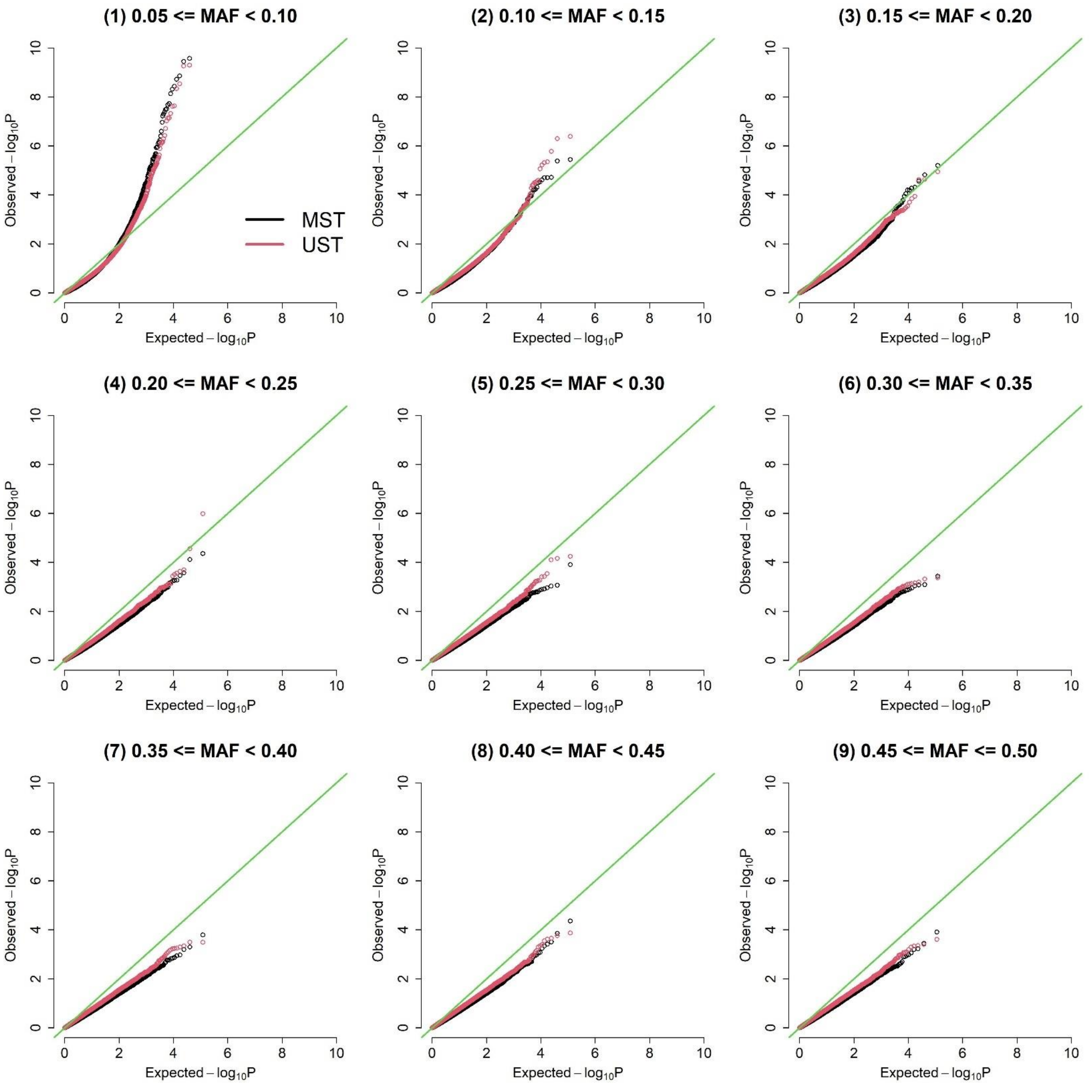

Figure S4. **The quantile-quantile (QQ) plots stratified by the nine ranges of MAFs (three traits, each from a Chi-squared distribution with the degree of freedom 1, exposure prevalence = 0.2, and *n* = 25,200).**

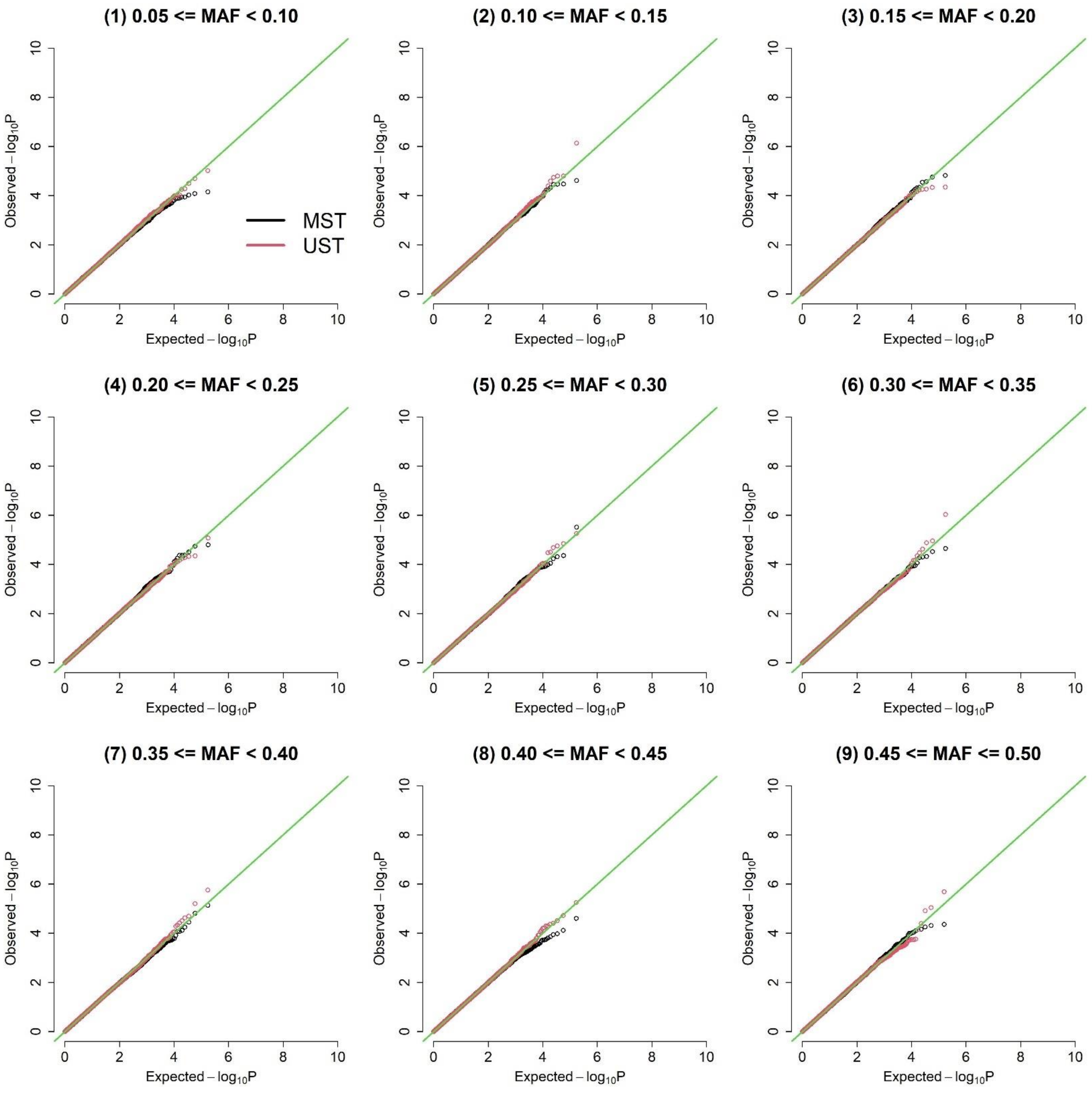

Figure S5. **The quantile-quantile (QQ) plots stratified by the nine ranges of MAFs (four traits from a multivariate normal distribution, exposure prevalence = 0.2, and *n* = 93,708).**

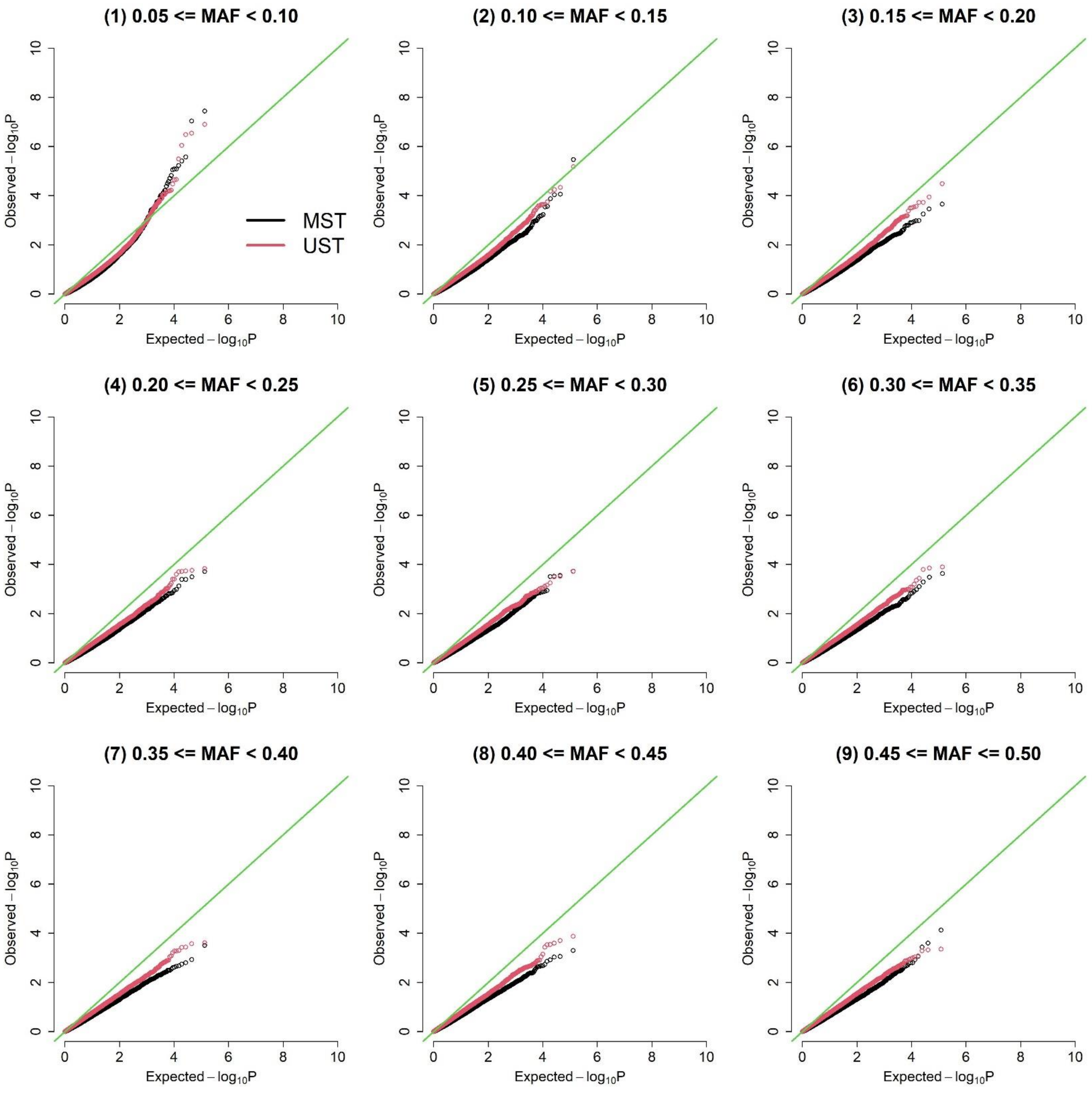

Figure S6. **The quantile-quantile (QQ) plots stratified by the nine ranges of MAFs (four traits, each from a Chi-squared distribution with the degree of freedom 1, exposure prevalence = 0.2, and *n* = 93,708).**

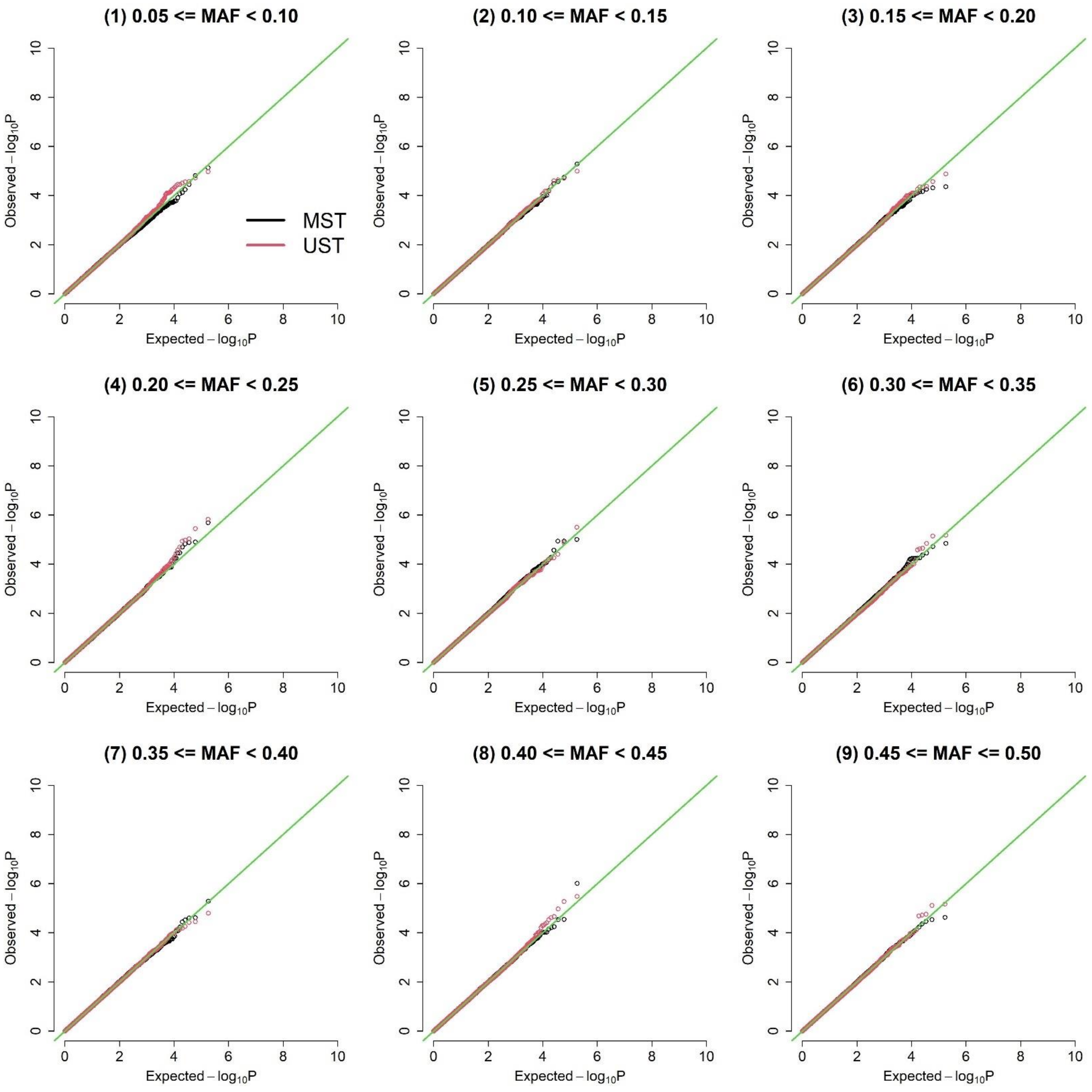

Figure S7. **The quantile-quantile (QQ) plots stratified by the nine ranges of MAFs (four traits from a multivariate normal distribution, exposure prevalence = 0.2, and *n* = 25,200).**

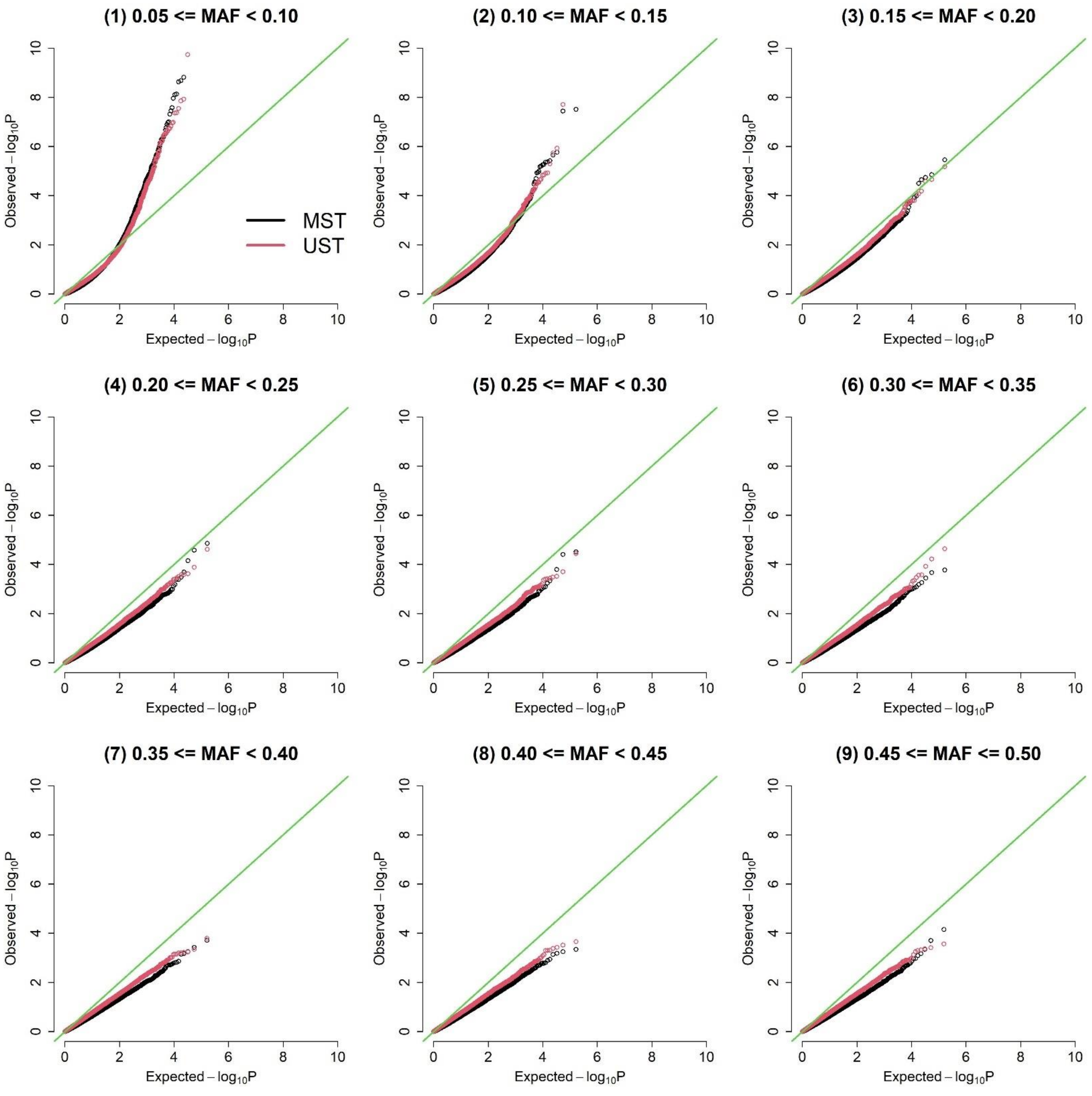

Figure S8. **The quantile-quantile (QQ) plots stratified by the nine ranges of MAFs (four traits, each from a Chi-squared distribution with the degree of freedom 1, exposure prevalence = 0.2, and *n* = 25,200).**

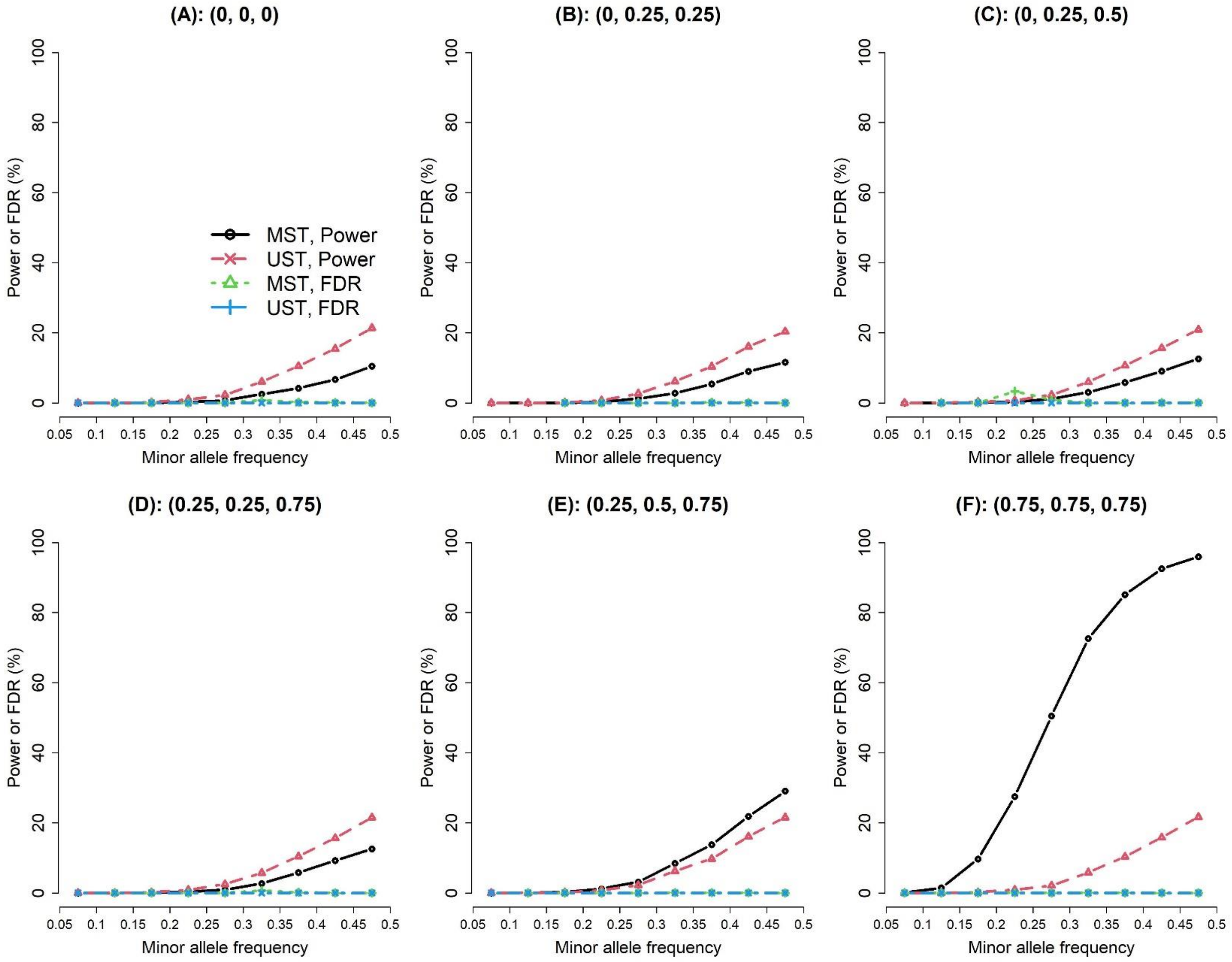

Figure S9. **Power and false discovery rate (FDR) of testing GxE for individual traits, when GxE influenced the first trait (three traits, each from a Chi-squared distribution with the degree of freedom 1, exposure prevalence = 0.2, and *n* = 93,708).**

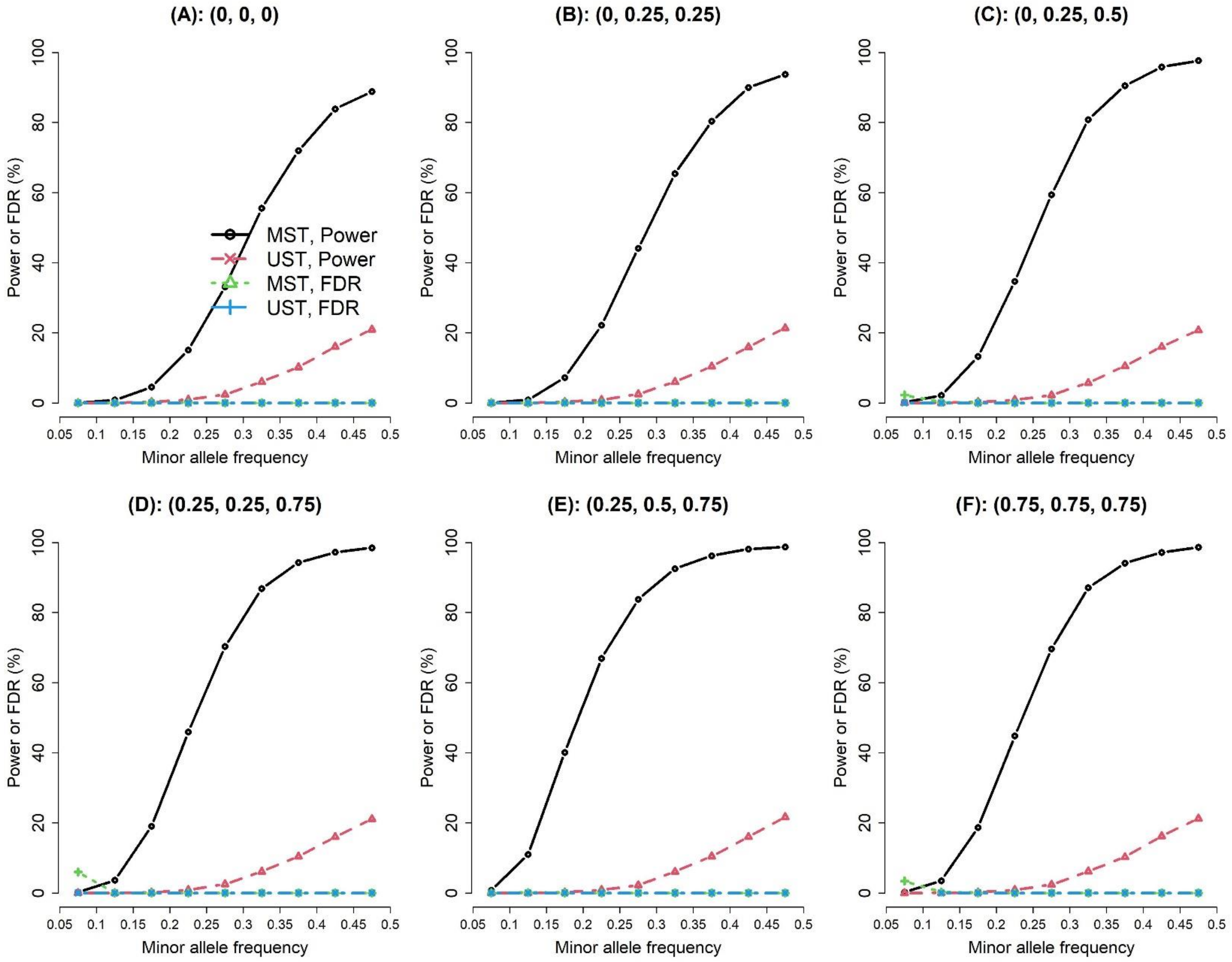

Figure S10. **Power and false discovery rate (FDR) of testing GxE for individual traits, when GxE influenced the first and the second traits (three traits, each from a Chi-squared distribution with the degree of freedom 1, exposure prevalence = 0.2, and *n* = 93,708).**

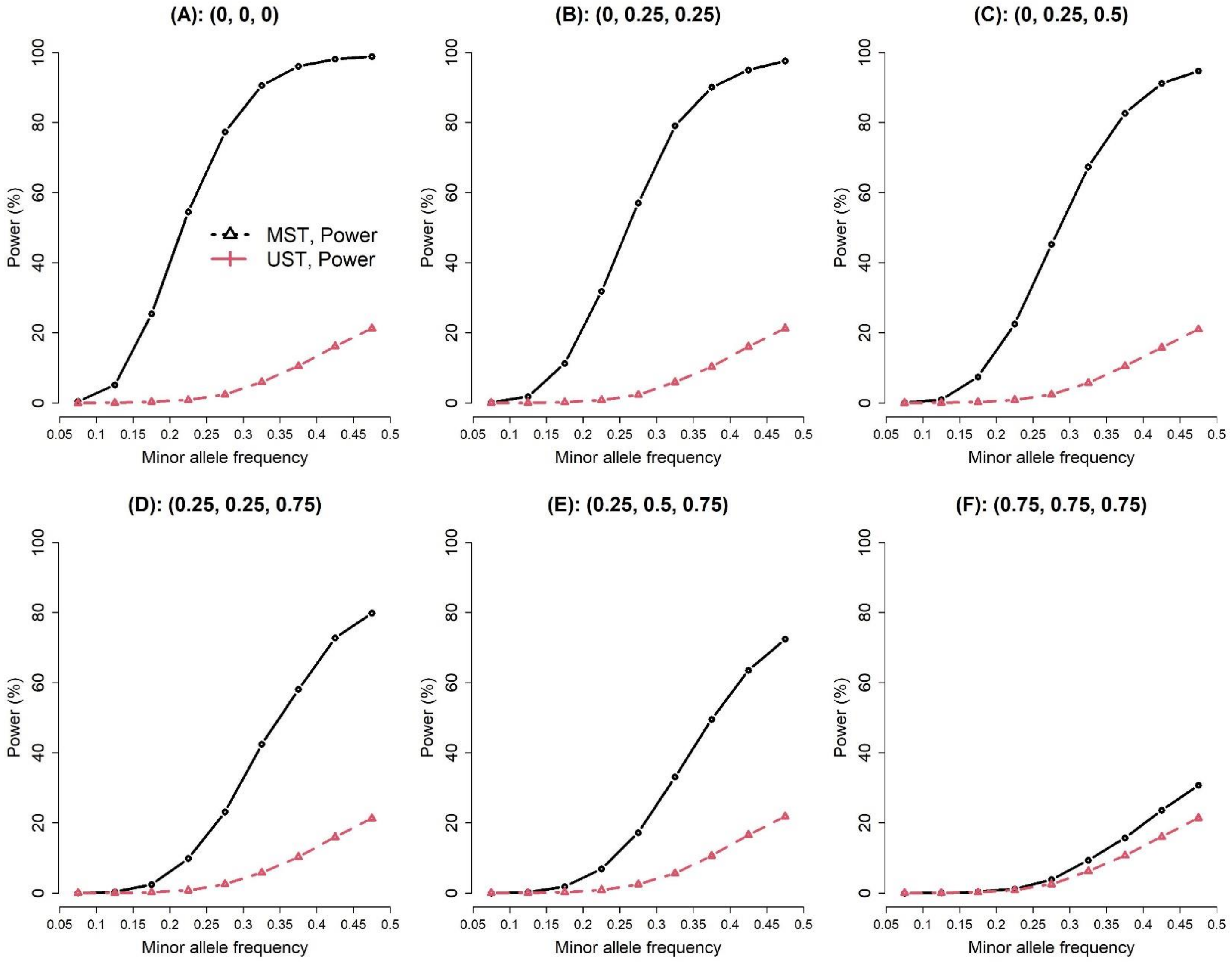

Figure S11. **Power of testing GxE for individual traits, when GxE influenced all three traits (three traits, each from a Chi-squared distribution with the degree of freedom 1, exposure prevalence = 0.2, and *n* = 93,708).**

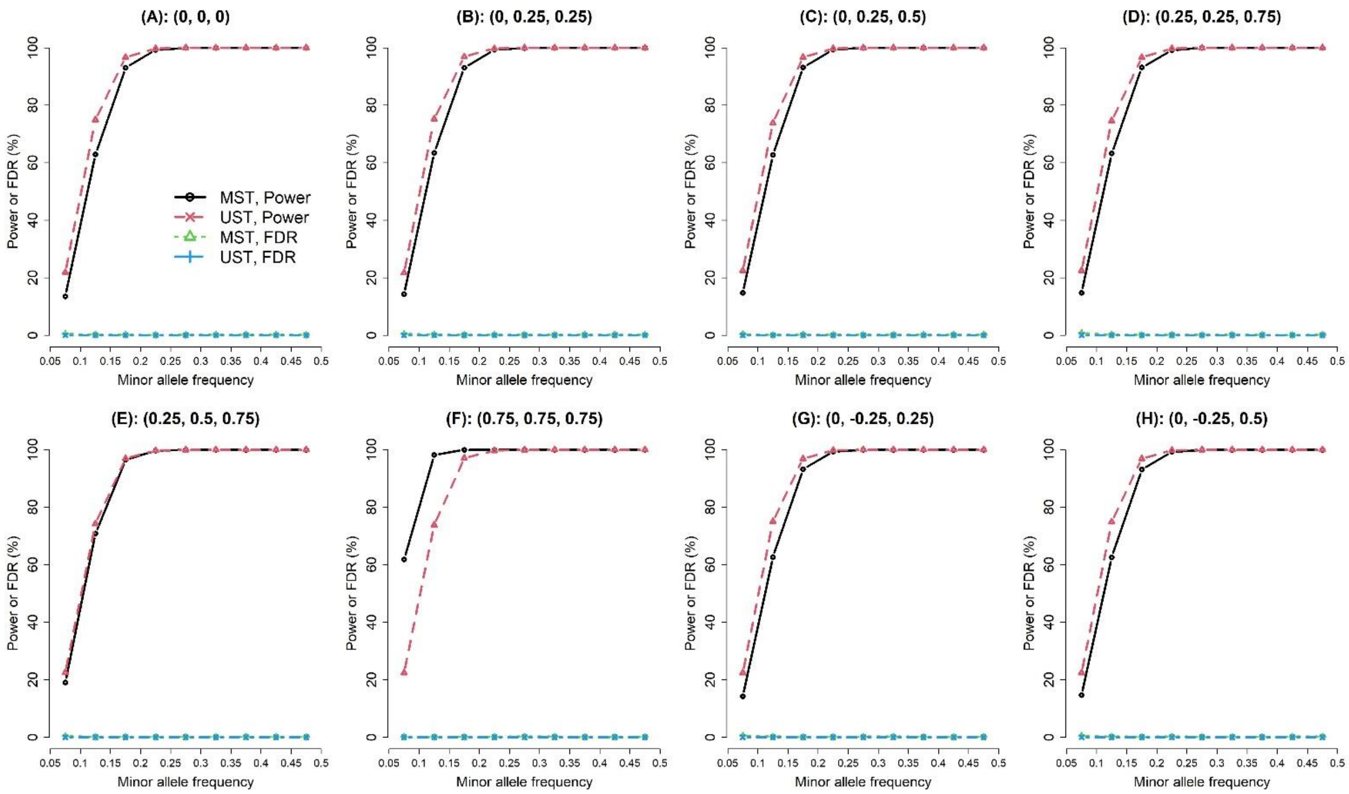

Figure S12. **Power and false discovery rate (FDR) of testing GxE for individual traits, when GxE influenced the first trait (three traits from a multivariate normal distribution, exposure prevalence = 0.5, and *n* = 93,708).**

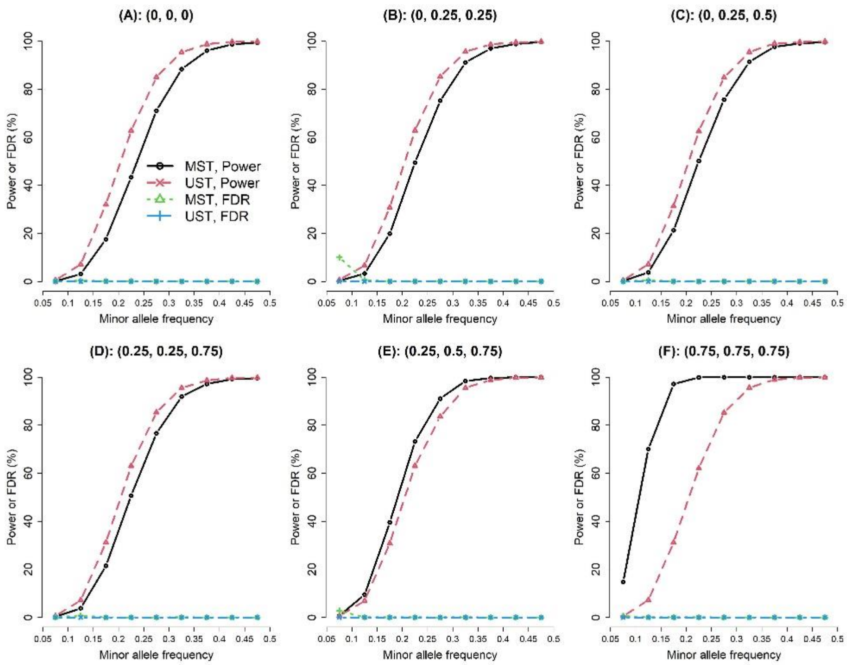

Figure S13. **Power and false discovery rate (FDR) of testing GxE for individual traits, when GxE influenced the first trait (three traits, each from a Chi-squared distribution with the degree of freedom 1, exposure prevalence = 0.5, and *n* = 93,708).**

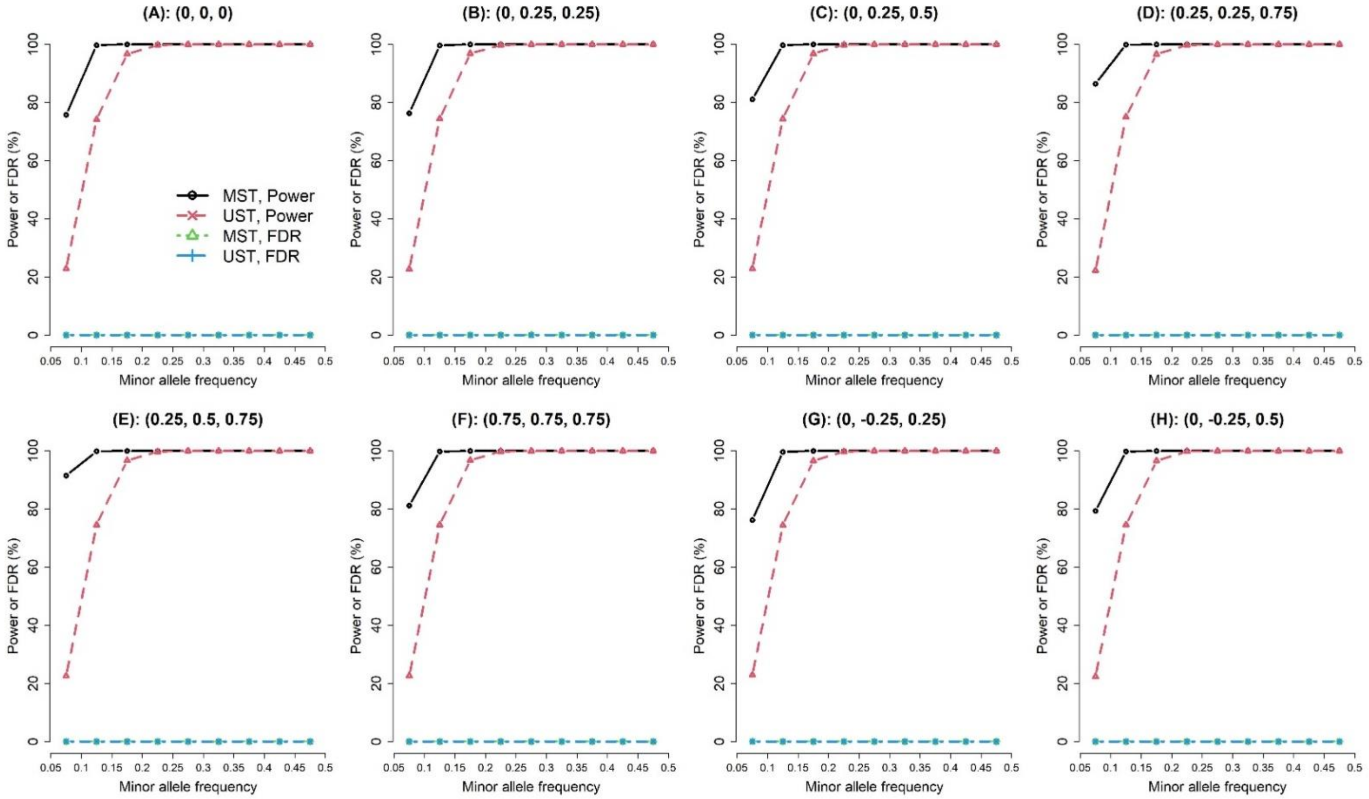

Figure S14. **Power and false discovery rate (FDR) of testing GxE for individual traits, when GxE influenced the first and the second traits (three traits from a multivariate normal distribution, exposure prevalence = 0.5, and *n* = 93,708).**

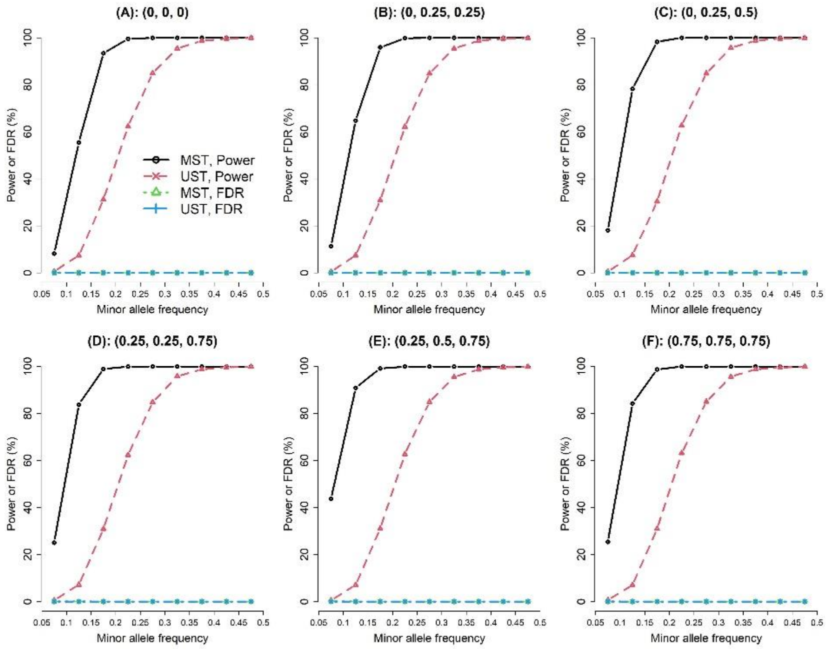

Figure S15. **Power and false discovery rate (FDR) of testing GxE for individual traits, when GxE influenced the first and the second traits (three traits, each from a Chi-squared distribution with the degree of freedom 1, exposure prevalence = 0.5, and *n* = 93,708).**

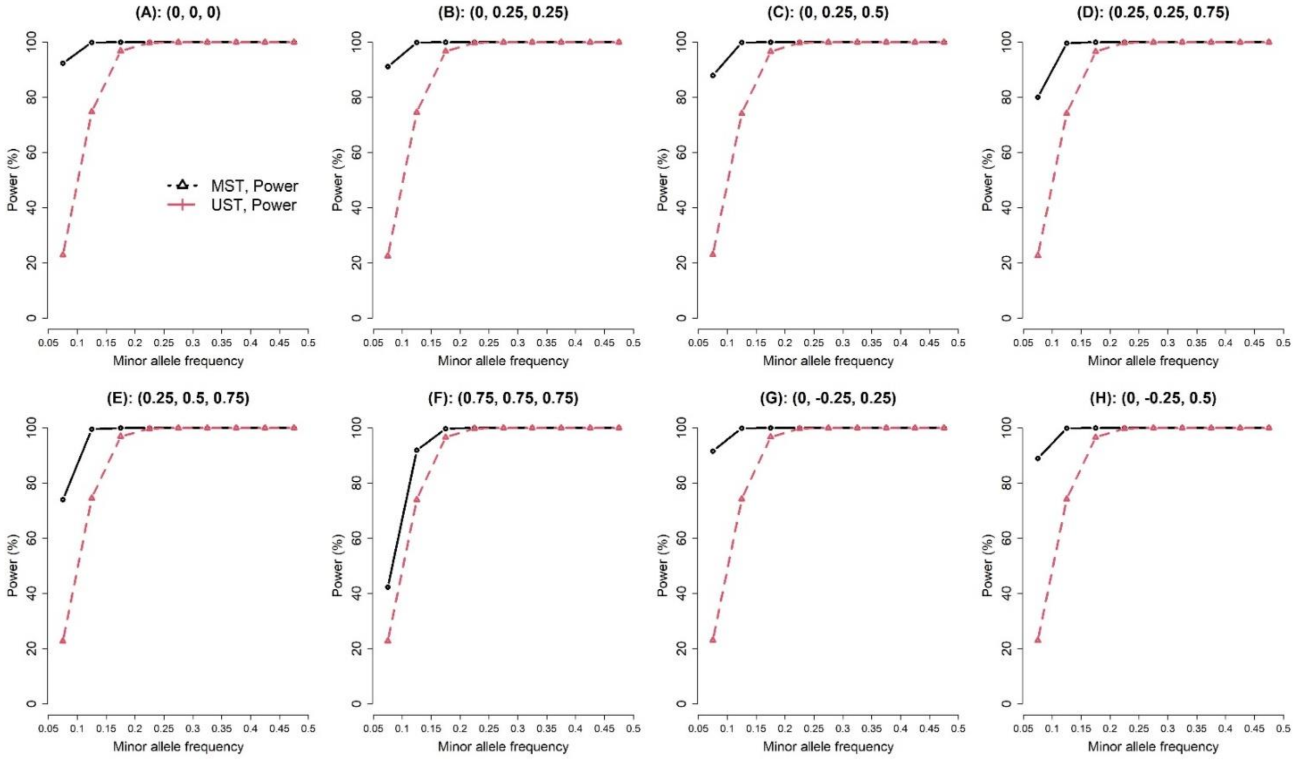

Figure S16. **Power of testing GxE for individual traits, when GxE influenced all three traits (three traits from a multivariate normal distribution, exposure prevalence = 0.5, and *n* = 93,708).**

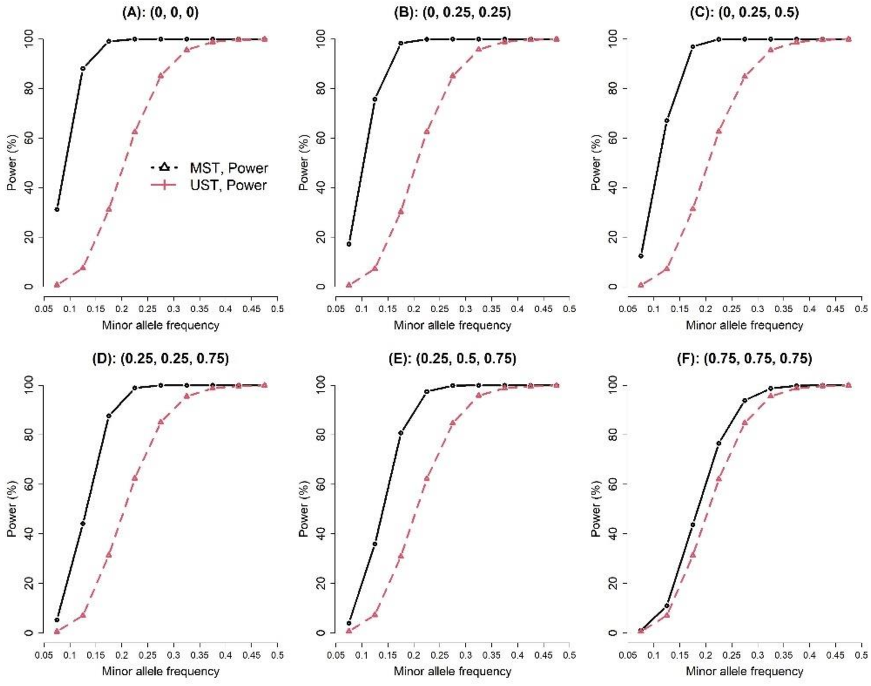

Figure S17. **Power of testing GxE for individual traits, when GxE influenced all three traits (three traits, each from a Chi-squared distribution with the degree of freedom 1, exposure prevalence = 0.5, and *n* = 93,708).**

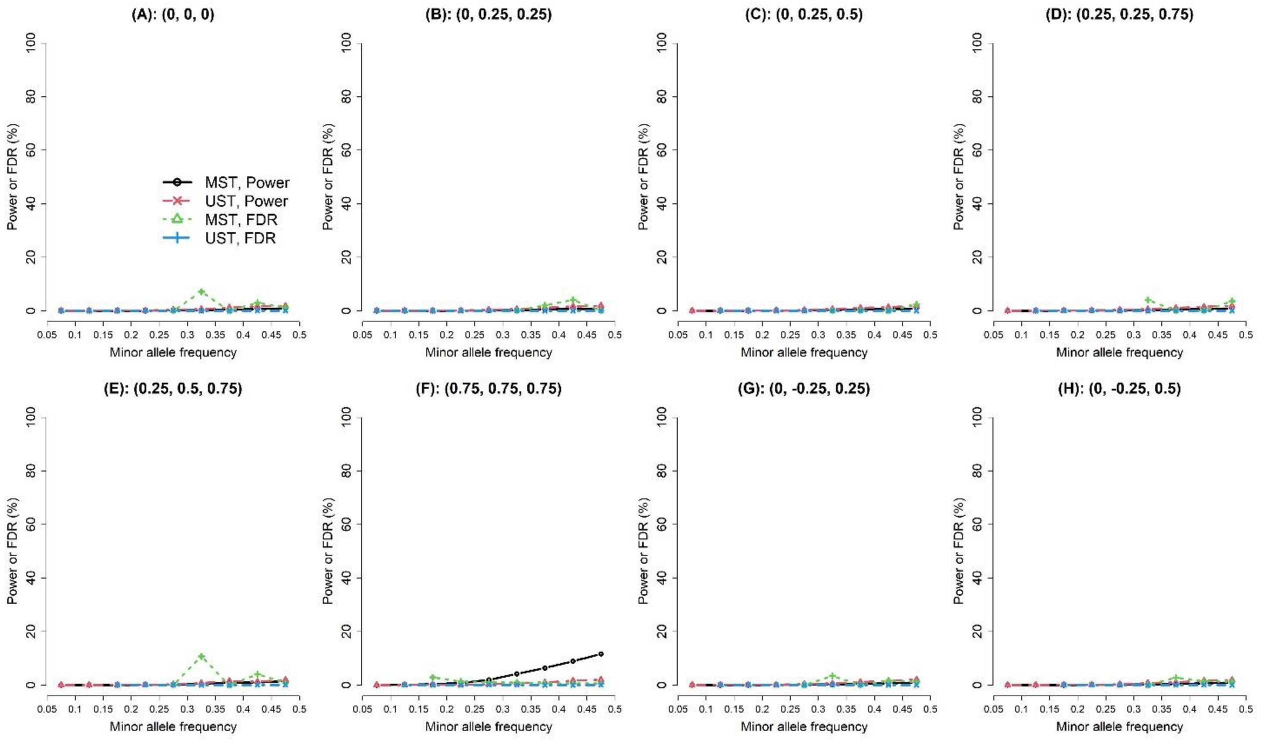

Figure S18. **Power and false discovery rate (FDR) of testing GxE for individual traits, when GxE influenced the first trait (three traits from a multivariate normal distribution, exposure prevalence = 0.2, and *n* = 25,200).** Some FDR levels were missing values due to no discoveries.

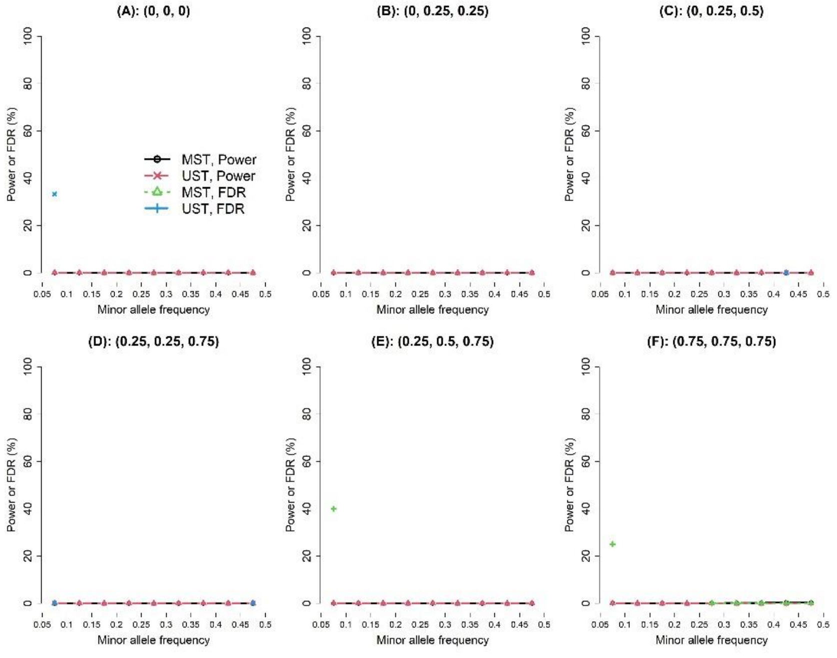

Figure S19. **Power and false discovery rate (FDR) of testing GxE for individual traits, when GxE influenced the first trait (three traits, each from a Chi-squared distribution with the degree of freedom 1, exposure prevalence = 0.2, and *n* = 25,200).** Some FDR levels were missing values due to no discoveries.

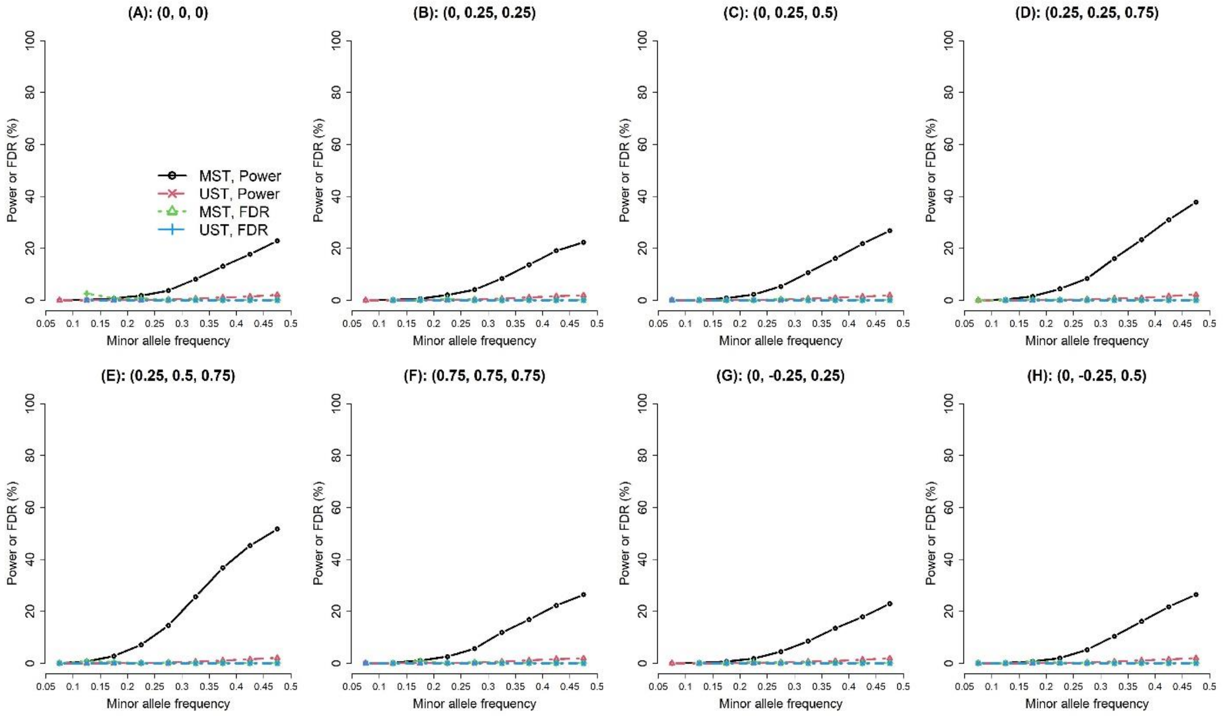

Figure S20. **Power and false discovery rate (FDR) of testing GxE for individual traits, when GxE influenced the first and the second traits (three traits from a multivariate normal distribution, exposure prevalence = 0.2, and *n* = 25,200).** Some FDR levels were missing values due to no discoveries.

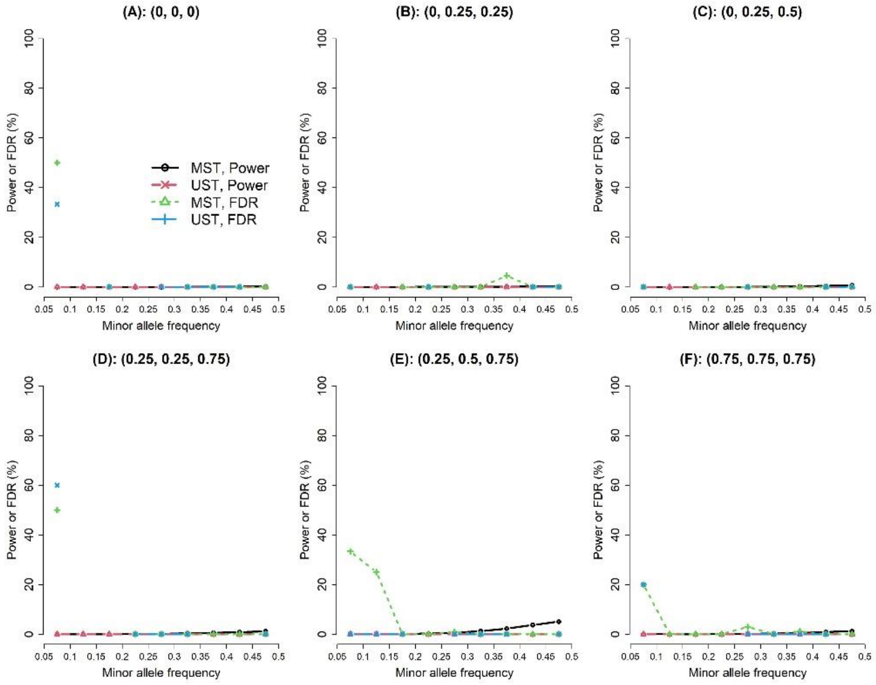

Figure S21. **Power and false discovery rate (FDR) of testing GxE for individual traits, when GxE influenced the first and the second traits (three traits, each from a Chi-squared distribution with the degree of freedom 1, exposure prevalence = 0.2, and *n* = 25,200).** Some FDR levels were missing values due to no discoveries.

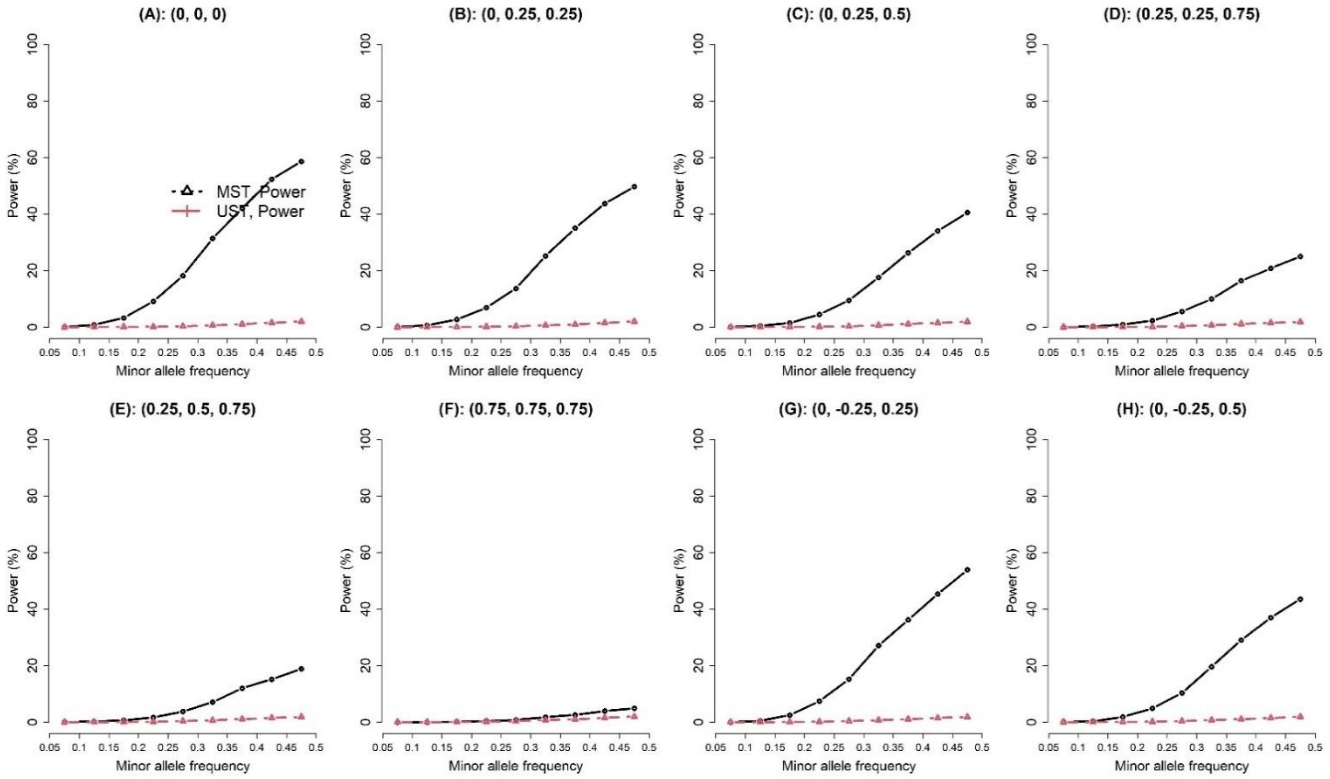

Figure S22. **Power of testing GxE for individual traits, when GxE influenced all three traits (three traits from a multivariate normal distribution, exposure prevalence = 0.2, and *n* = 25,200).**

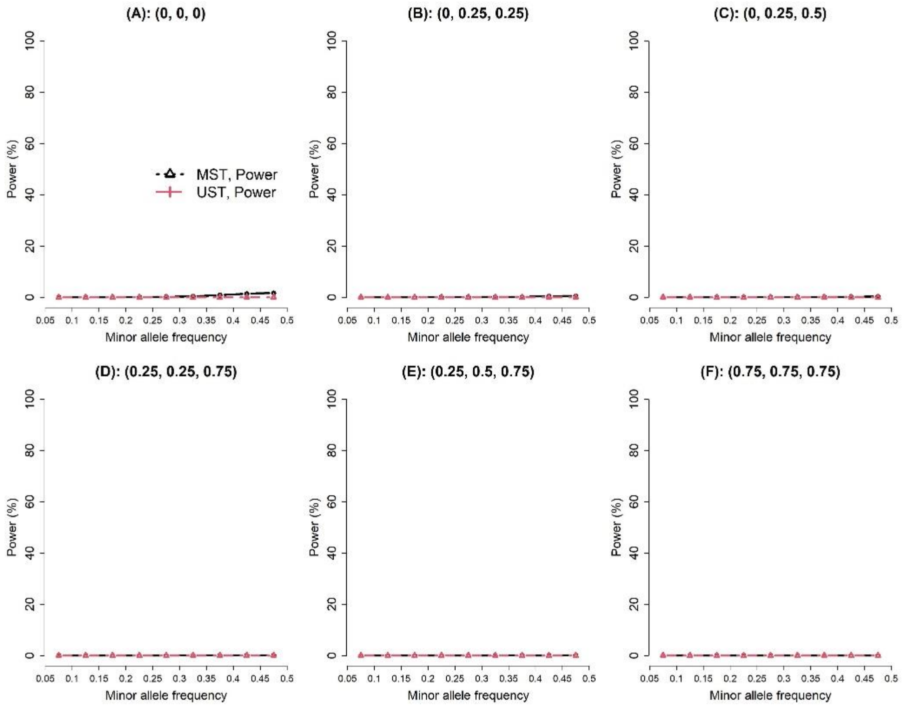

Figure S23. **Power of testing GxE for individual traits, when GxE influenced all three traits (three traits, each from a Chi-squared distribution with the degree of freedom 1, exposure prevalence = 0.2, and *n* = 25,200).**

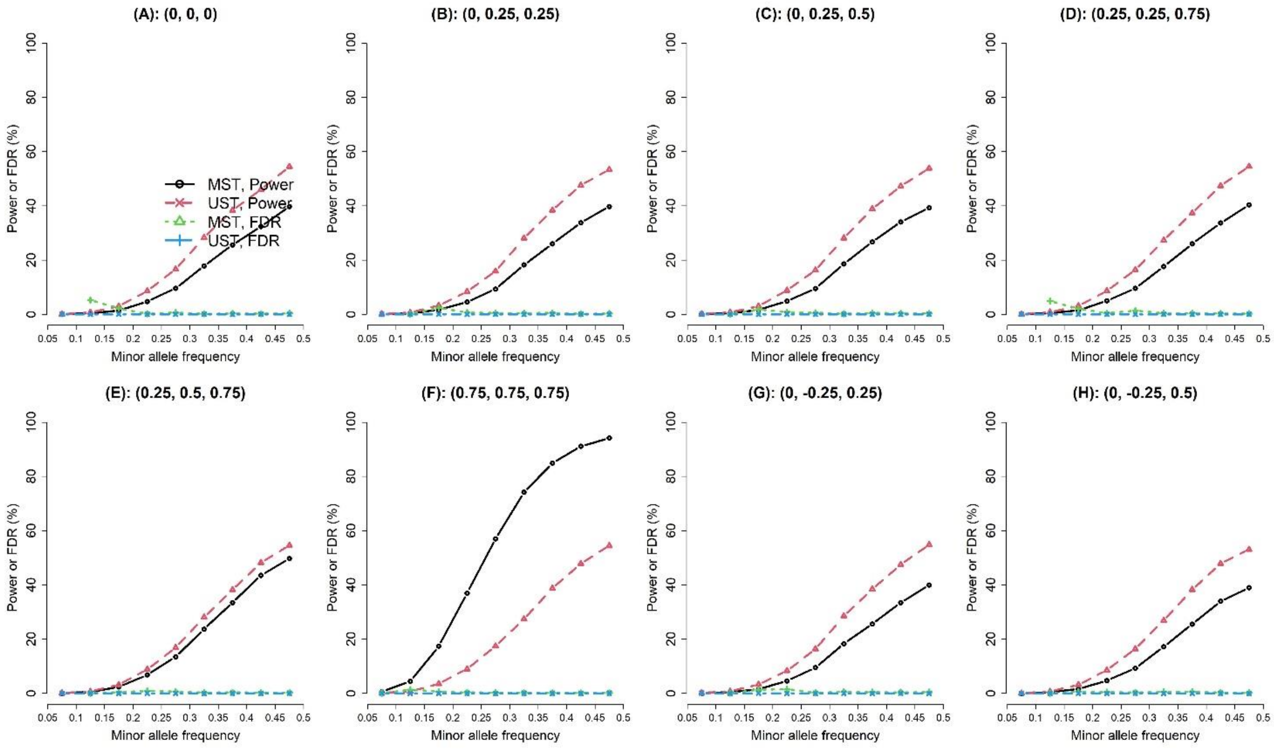

Figure S24. **Power and false discovery rate (FDR) of testing GxE for individual traits, when GxE influenced the first trait (three traits from a multivariate normal distribution, exposure prevalence = 0.5, and *n* = 25,200).** Some FDR levels were missing values due to no discoveries.

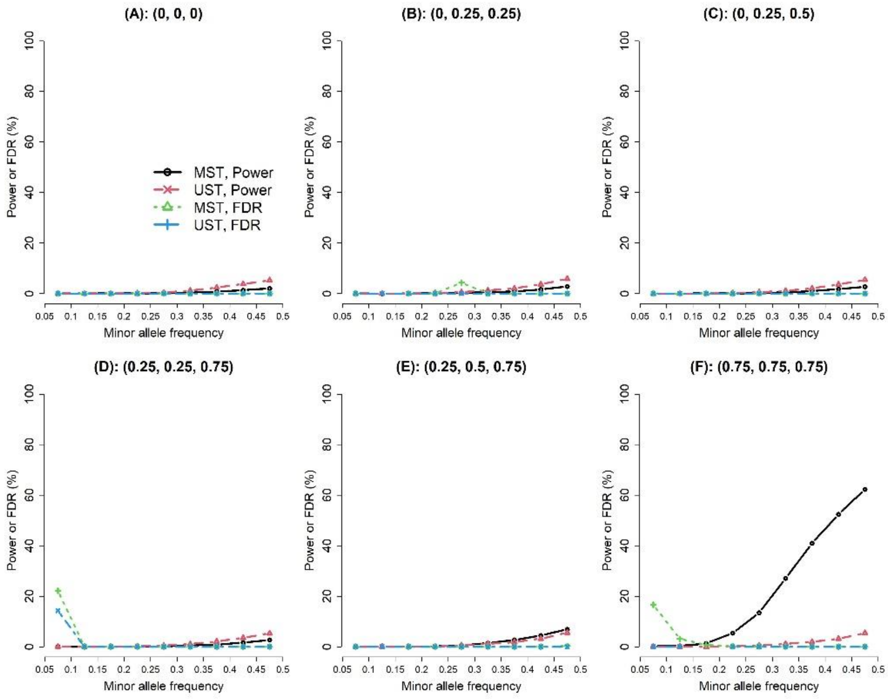

Figure S25. **Power and false discovery rate (FDR) of testing GxE for individual traits, when GxE influenced the first trait (three traits, each from a Chi-squared distribution with the degree of freedom 1, exposure prevalence = 0.5, and *n* = 25,200).** Some FDR levels were missing values due to no discoveries.

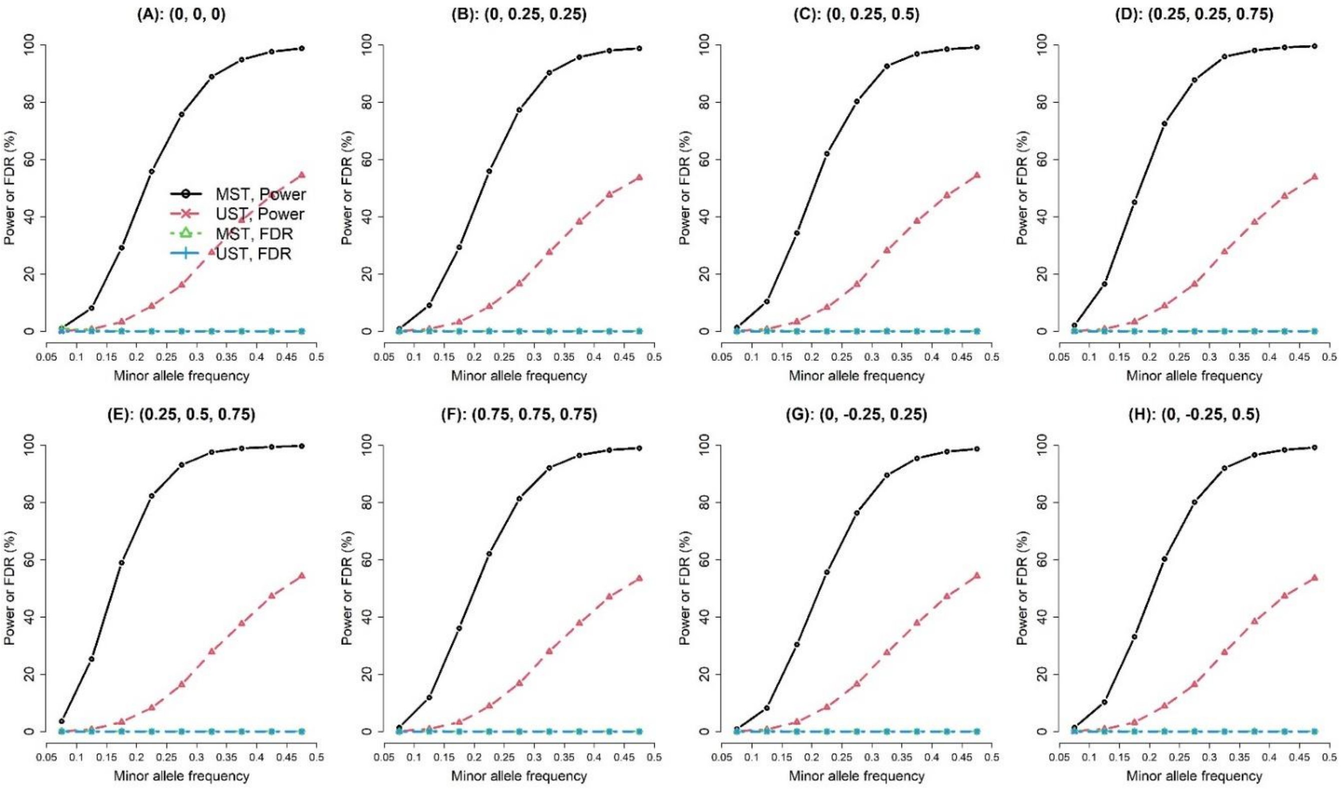

Figure S26. **Power and false discovery rate (FDR) of testing GxE for individual traits, when GxE influenced the first and the second traits (three traits from a multivariate normal distribution, exposure prevalence = 0.5, and *n* = 25,200).**

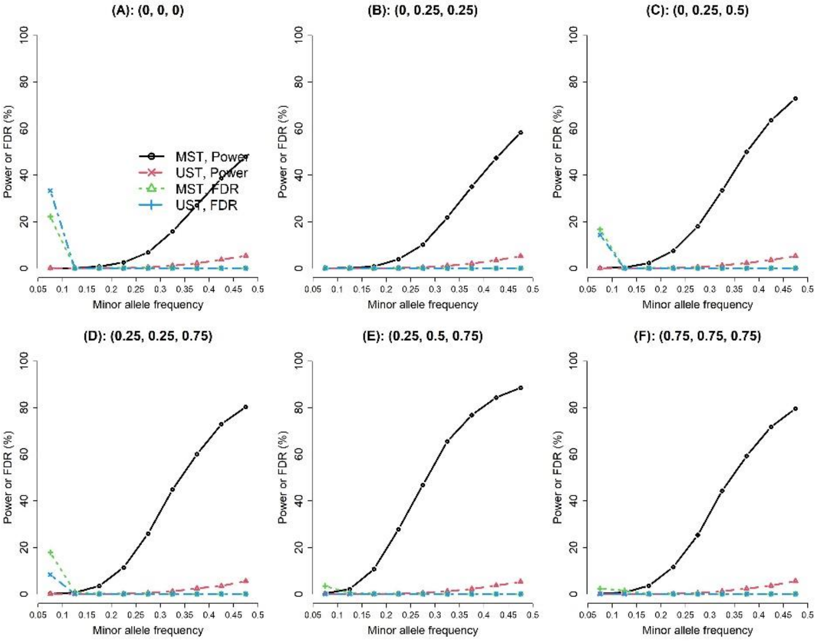

Figure S27. **Power and false discovery rate (FDR) of testing GxE for individual traits, when GxE influenced the first and the second traits (three traits, each from a Chi-squared distribution with the degree of freedom 1, exposure prevalence = 0.5, and *n* = 25,200).**

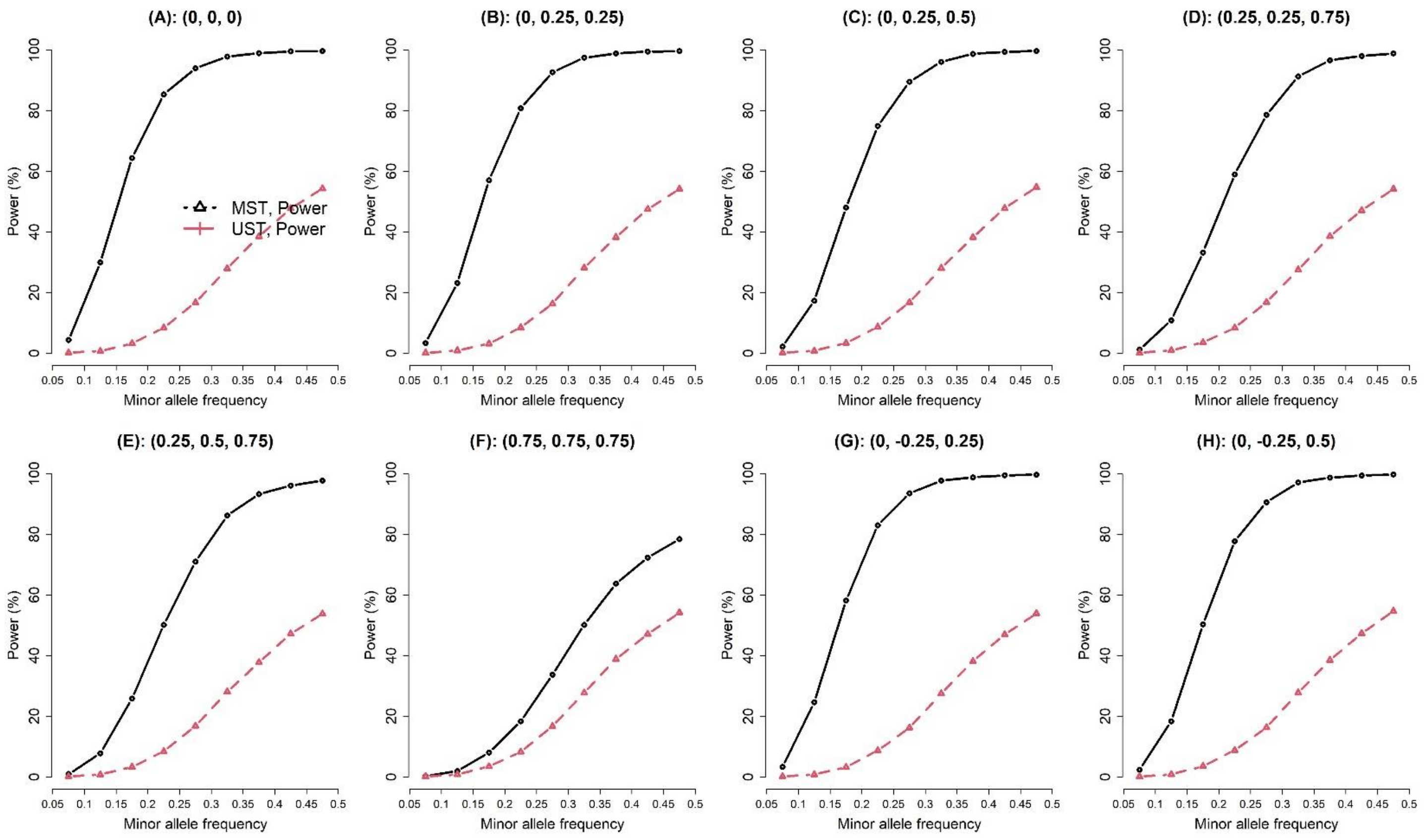

Figure S28. **Power of testing GxE for individual traits, when GxE influenced all three traits (three traits from a multivariate normal distribution, exposure prevalence = 0.5, and *n* = 25,200).**

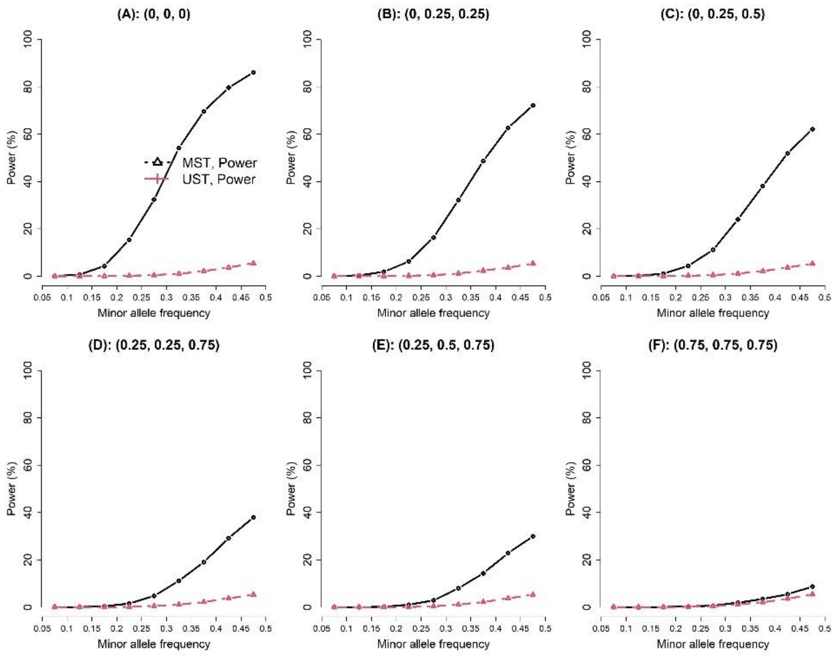

Figure S29. **Power of testing GxE for individual traits, when GxE influenced all three traits (three traits, each from a Chi-squared distribution with the degree of freedom 1, exposure prevalence = 0.5, and *n* = 25,200).**

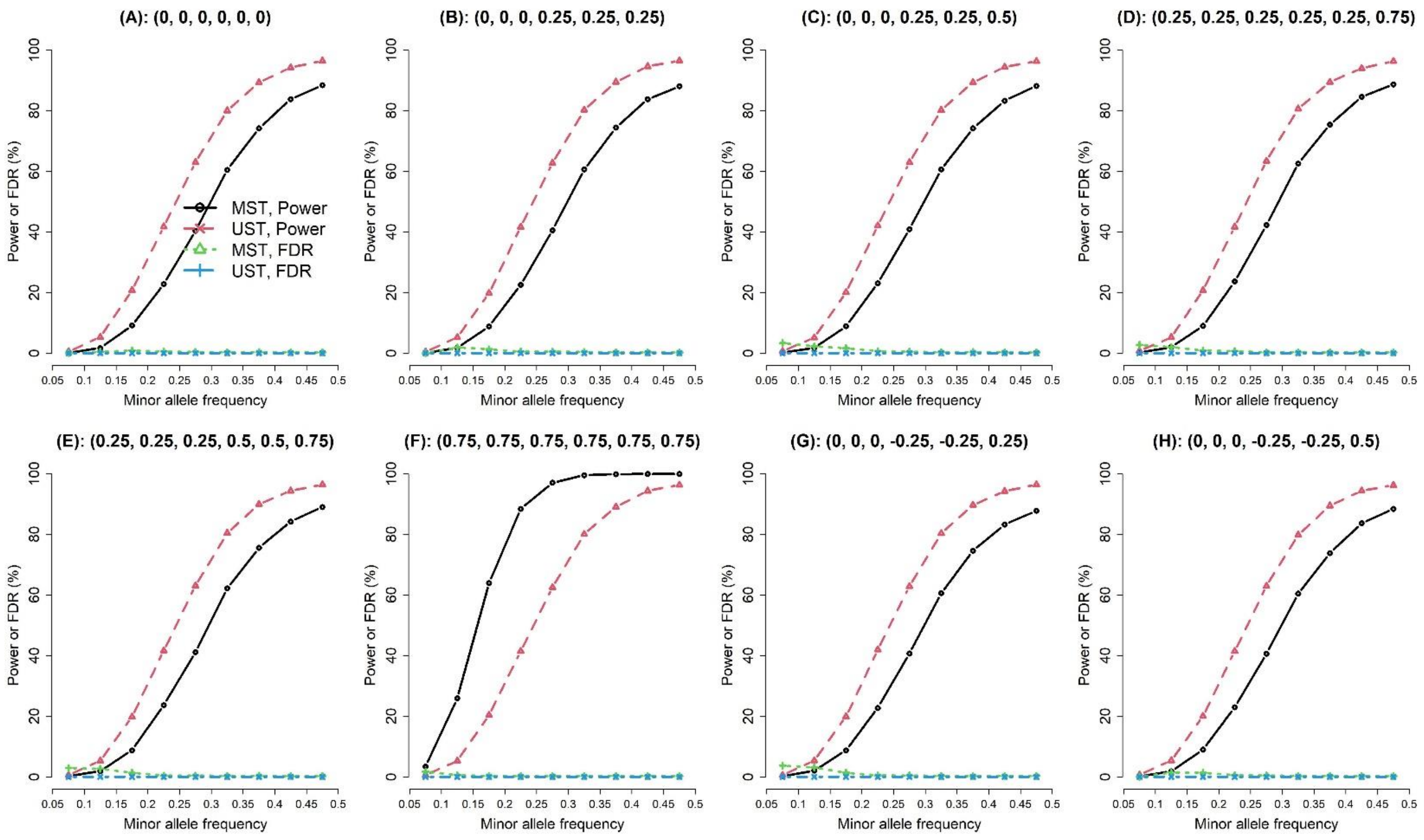

Figure S30. **Power and false discovery rate (FDR) of testing GxE for individual traits, when GxE influenced the first trait (four traits from a multivariate normal distribution, exposure prevalence = 0.2, and *n* = 93,708).** Each point was calculated based on 10,000 replications. The title of each plot represents $(\boldsymbol{\rho_{12}}, \boldsymbol{\rho_{13}}, \boldsymbol{\rho_{14}}, \boldsymbol{\rho_{23}}, \boldsymbol{\rho_{24}}, \boldsymbol{\rho_{34}})$, where $\boldsymbol{\rho_{uv}}$ denotes the correlation between the uth and the vth traits, and $u, v \in \{\mathbf{1}, \mathbf{2}, \mathbf{3}, \mathbf{4}\}$.

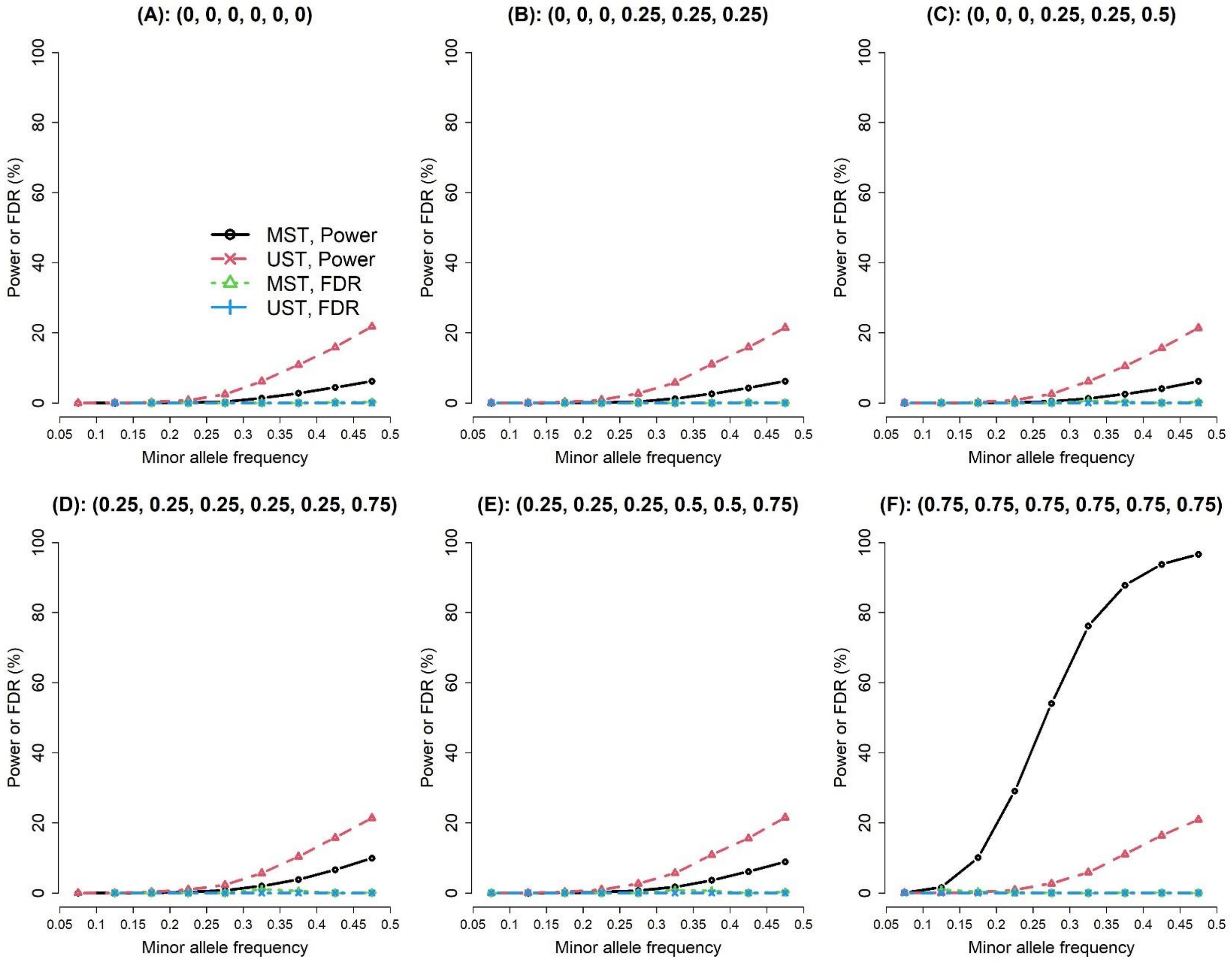

Figure S31. **Power and false discovery rate (FDR) of testing GxE for individual traits, when GxE influenced the first trait (four traits, each from a Chi-squared distribution with the degree of freedom 1, exposure prevalence = 0.2, and *n* = 93,708).** Each point was calculated based on 10,000 replications. The title of each plot represents $(\boldsymbol{\rho_{12}}, \boldsymbol{\rho_{13}}, \boldsymbol{\rho_{14}}, \boldsymbol{\rho_{23}}, \boldsymbol{\rho_{24}}, \boldsymbol{\rho_{34}})$, where $\boldsymbol{\rho_{uv}}$ denotes the correlation between the uth and the vth traits, and u, v $\in \{\mathbf{1}, \mathbf{2}, \mathbf{3}, \mathbf{4}\}$.

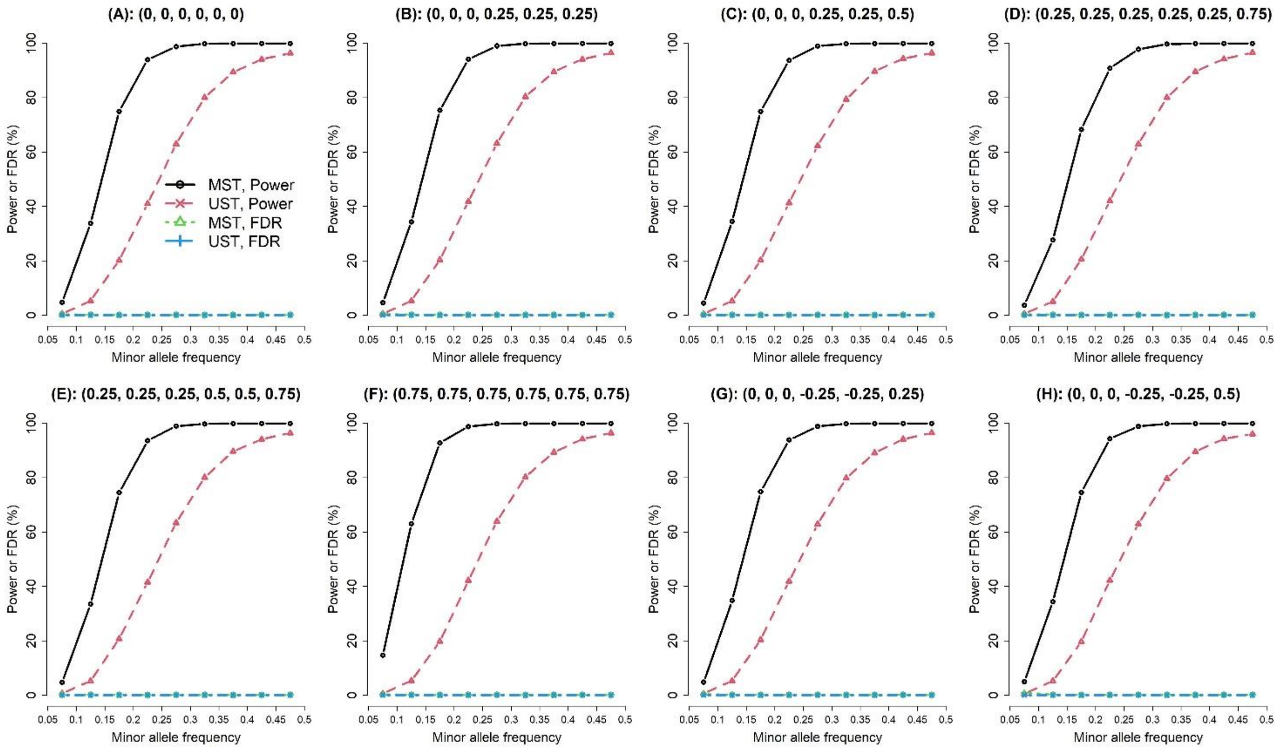

Figure S32. **Power and false discovery rate (FDR) of testing GxE for individual traits, when GxE influenced the first and the second traits (four traits from a multivariate normal distribution, exposure prevalence = 0.2, and *n* = 93,708).**

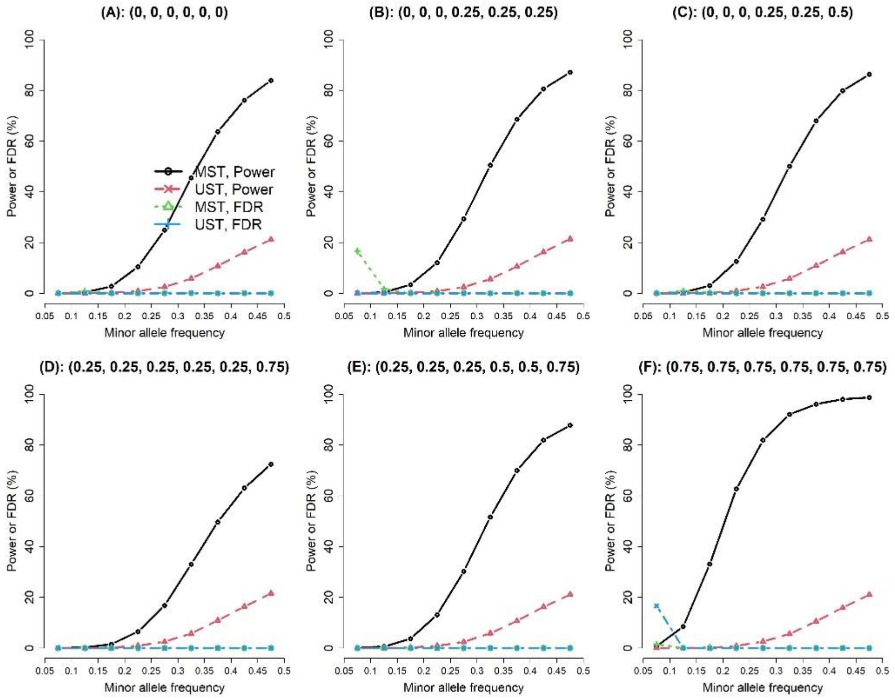

Figure S33. **Power and false discovery rate (FDR) of testing GxE for individual traits, when GxE influenced the first and the second traits (four traits, each from a Chi-squared distribution with the degree of freedom 1, exposure prevalence = 0.2, and *n* = 93,708).**

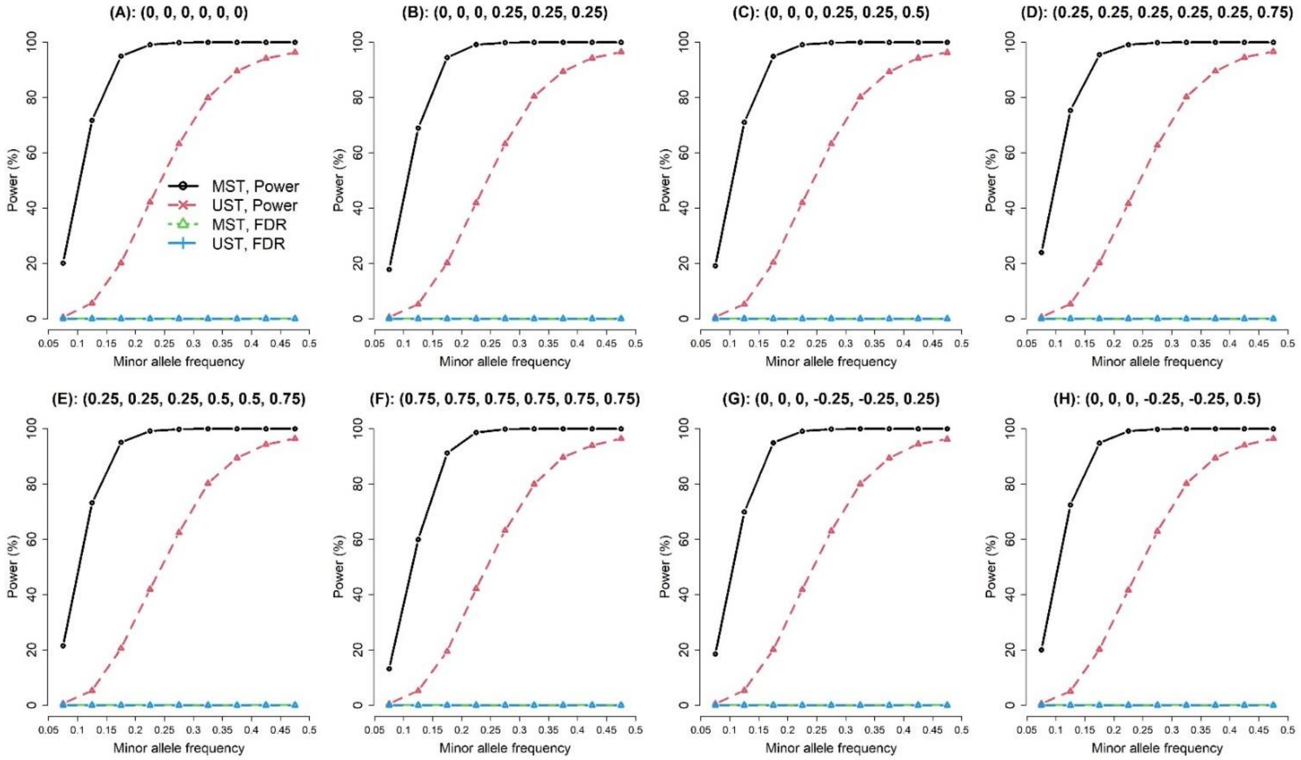

Figure S34. **Power and false discovery rate (FDR) of testing GxE for individual traits, when GxE influenced the first three traits (four traits from a multivariate normal distribution, exposure prevalence = 0.2, and *n* = 93,708).**

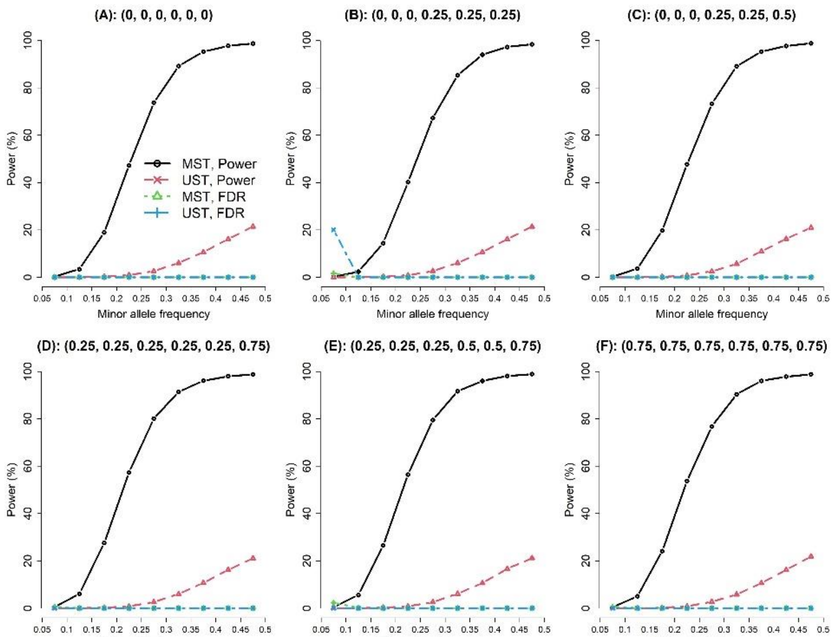

Figure S35. **Power and false discovery rate (FDR) of testing GxE for individual traits, when GxE influenced the first three traits (four traits, each from a Chi-squared distribution with the degree of freedom 1, exposure prevalence = 0.2, and *n* = 93,708).**

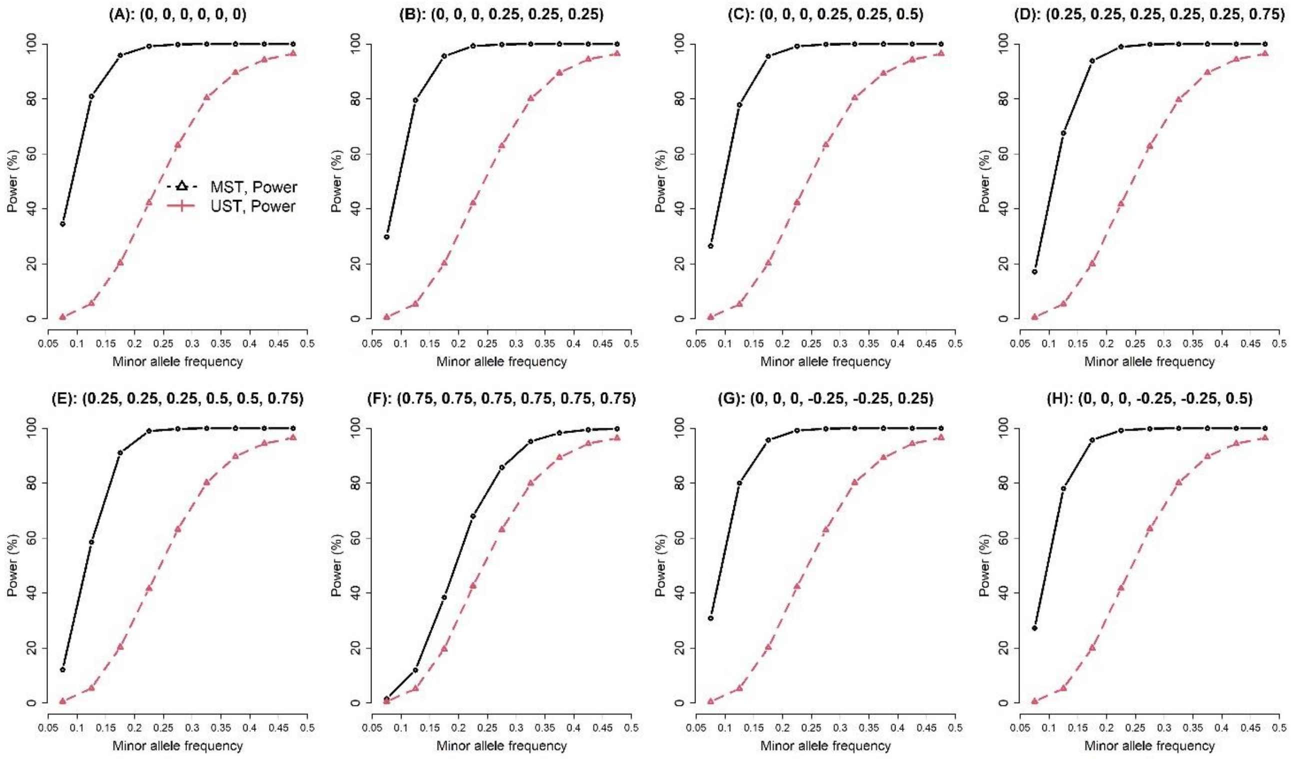

Figure S36. **Power of testing GxE for individual traits, when GxE influenced all four traits (four traits from a multivariate normal distribution, exposure prevalence = 0.2, and *n* = 93,708).**

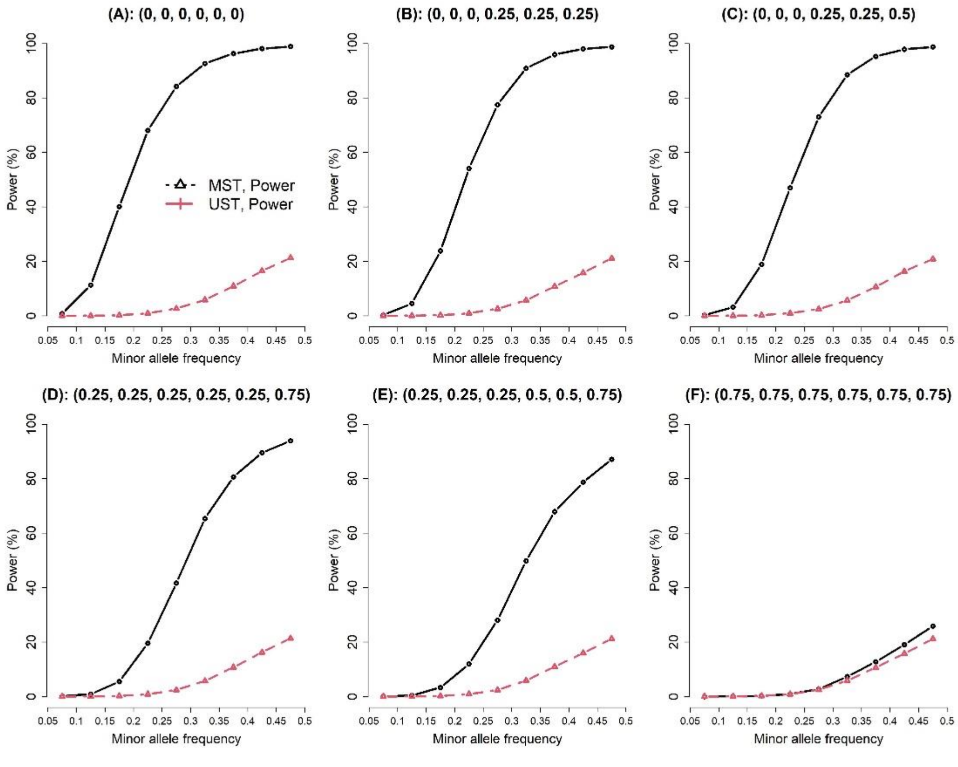

Figure S37. **Power of testing GxE for individual traits, when GxE influenced all four traits (four traits, each from a Chi-squared distribution with the degree of freedom 1, exposure prevalence = 0.2, and *n* = 93,708).**

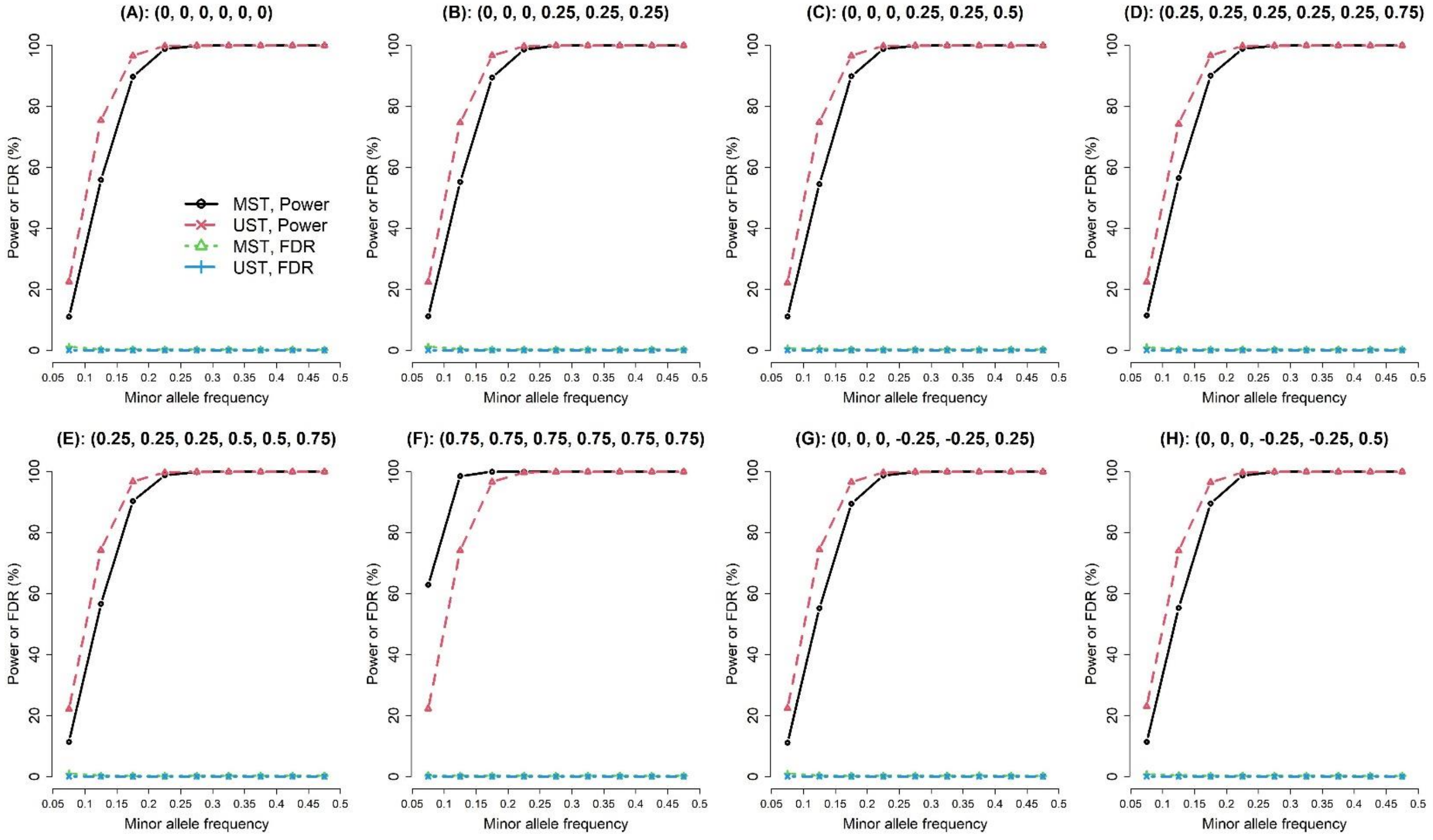

Figure S38. **Power and false discovery rate (FDR) of testing GxE for individual traits, when GxE influenced the first trait (four traits from a multivariate normal distribution, exposure prevalence = 0.5, and *n* = 93,708).**

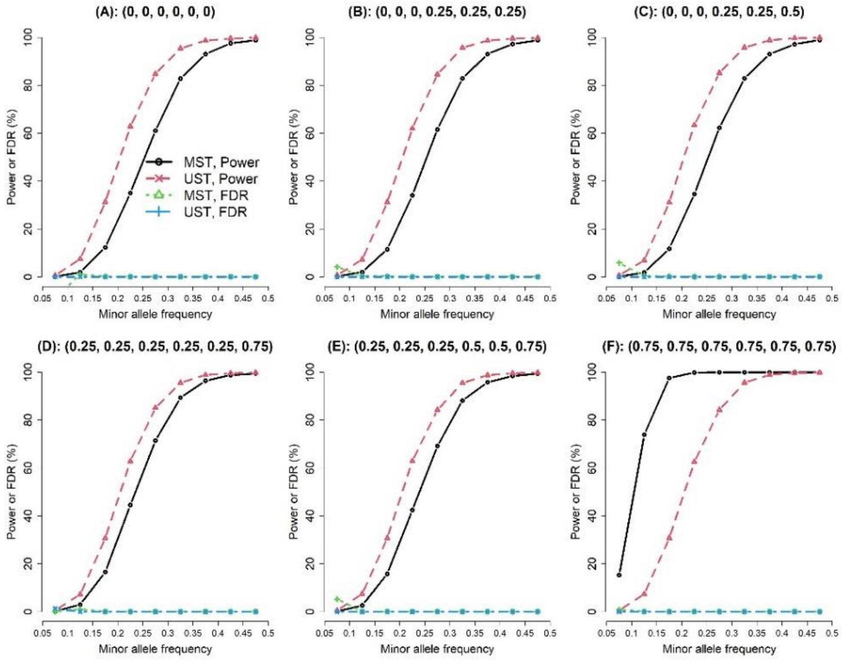

Figure S39. **Power and false discovery rate (FDR) of testing GxE for individual traits, when GxE influenced the first trait (four traits, each from a Chi-squared distribution with the degree of freedom 1, exposure prevalence = 0.5, and *n* = 93,708).**

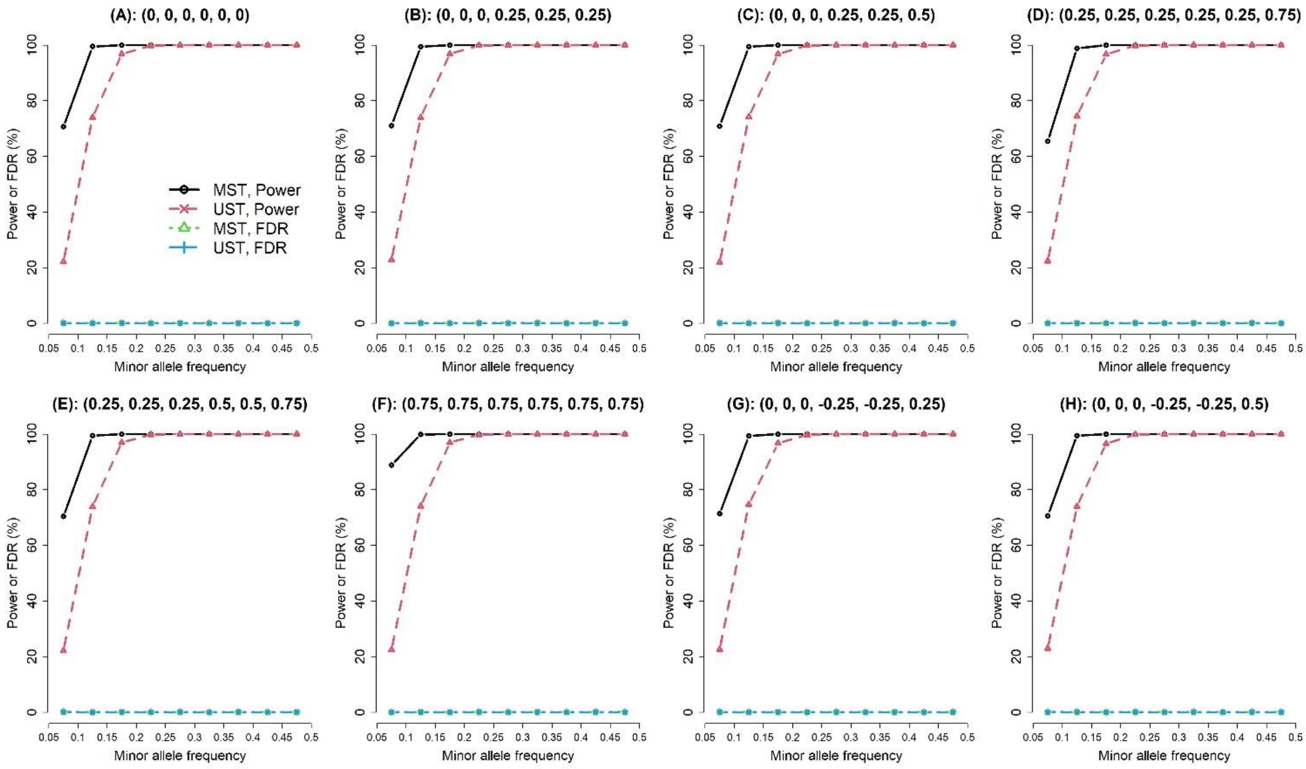

Figure S40. **Power and false discovery rate (FDR) of testing GxE for individual traits, when GxE influenced the first and the second traits (four traits from a multivariate normal distribution, exposure prevalence = 0.5, and *n* = 93,708).**

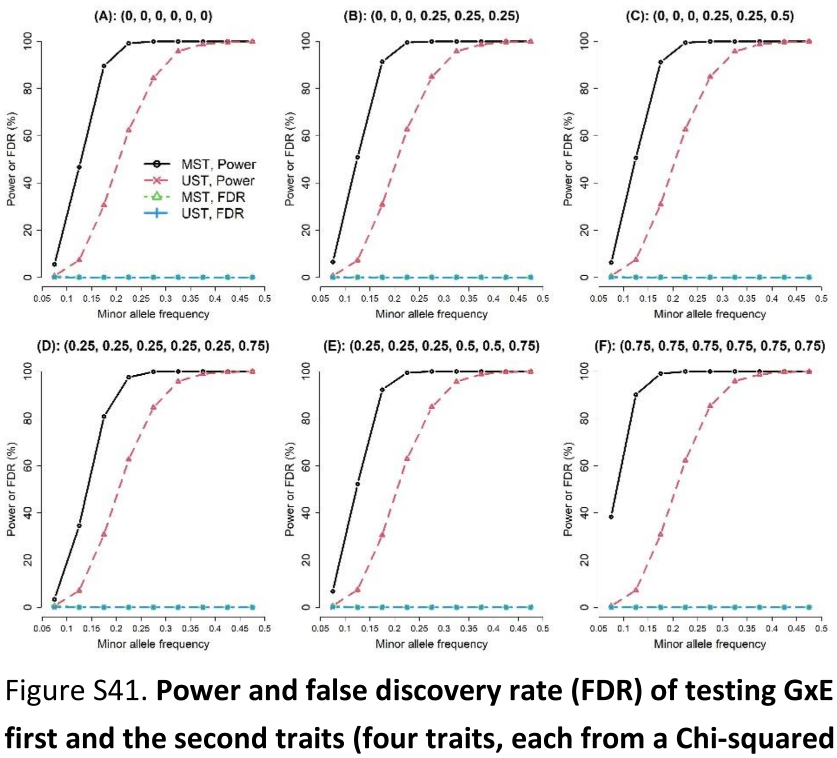

Figure S41. **Power and false discovery rate (FDR) of testing GxE for individual traits, when GxE influenced the first and the second traits (four traits, each from a Chi-squared distribution with the degree of freedom 1, exposure prevalence = 0.5, and *n* = 93,708).**

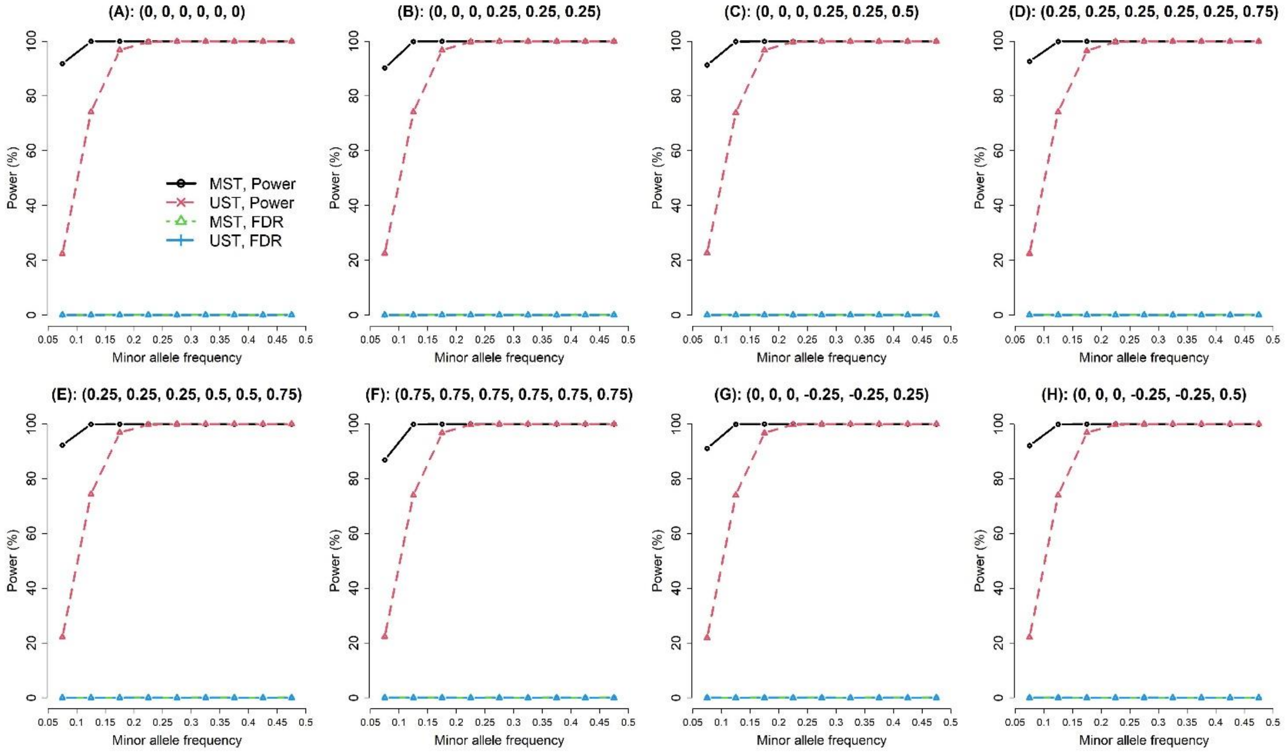

Figure S42. **Power and false discovery rate (FDR) of testing GxE for individual traits, when GxE influenced the first three traits (four traits from a multivariate normal distribution, exposure prevalence = 0.5, and *n* = 93,708).**

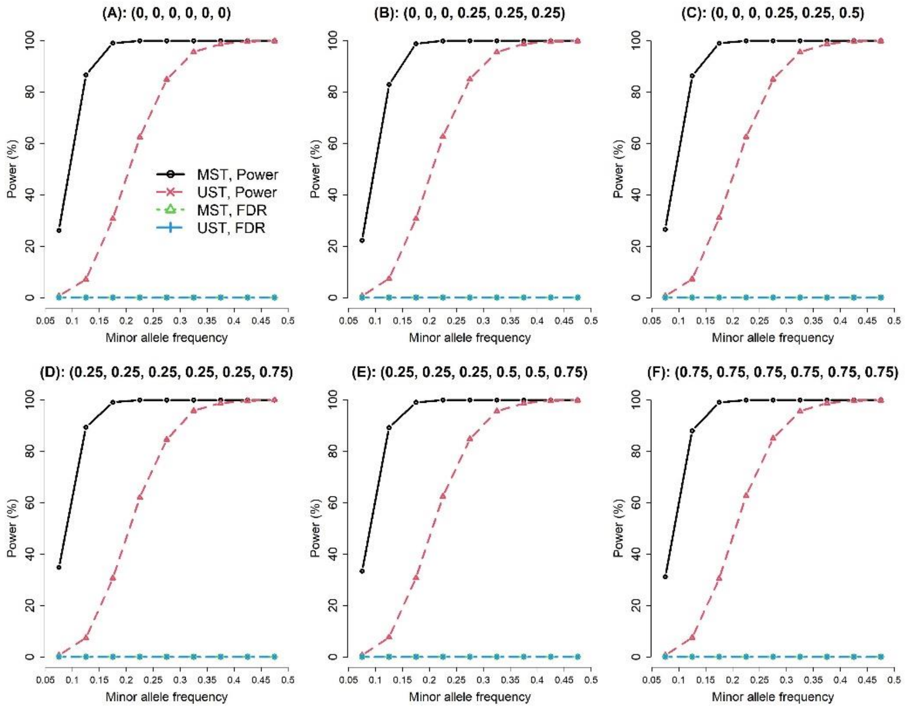

Figure S43. **Power and false discovery rate (FDR) of testing GxE for individual traits, when GxE influenced the first three traits (four traits, each from a Chi-squared distribution with the degree of freedom 1, exposure prevalence = 0.5, and *n* = 93,708).**

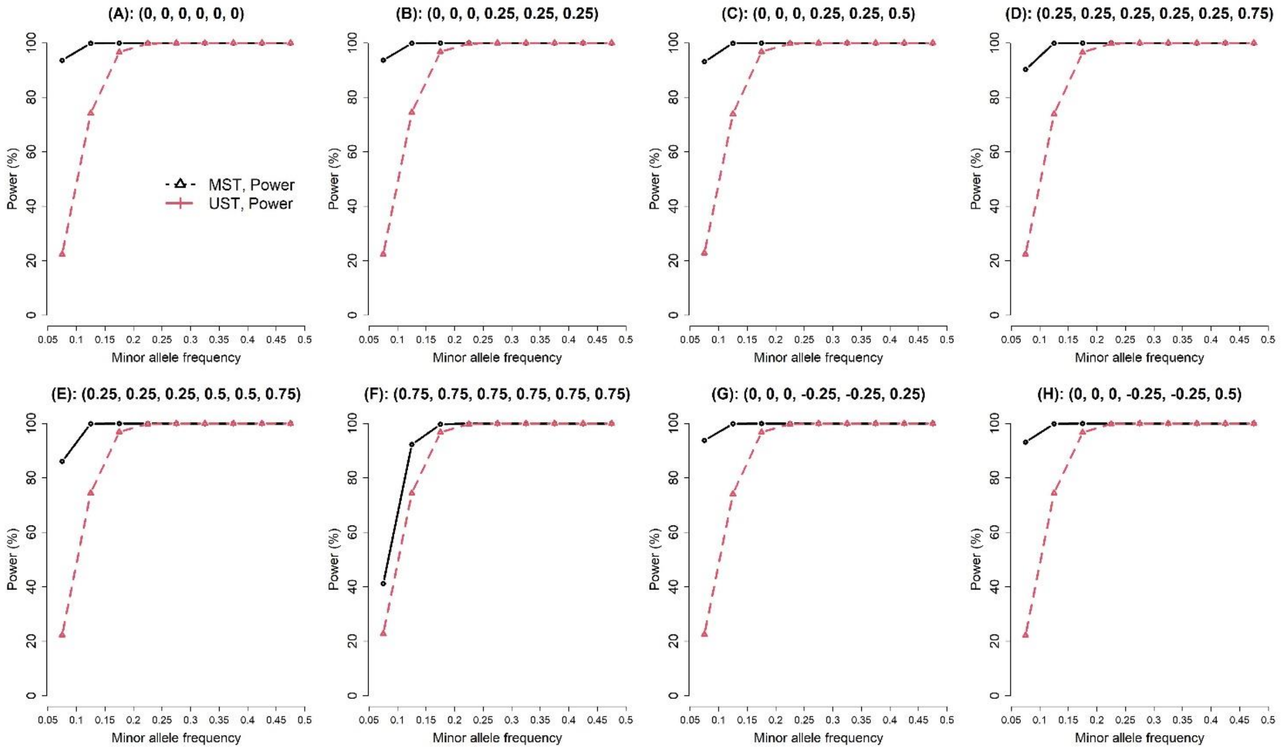

Figure S44. **Power of testing GxE for individual traits, when GxE influenced all four traits (four traits from a multivariate normal distribution, exposure prevalence = 0.5, and *n* = 93,708).**

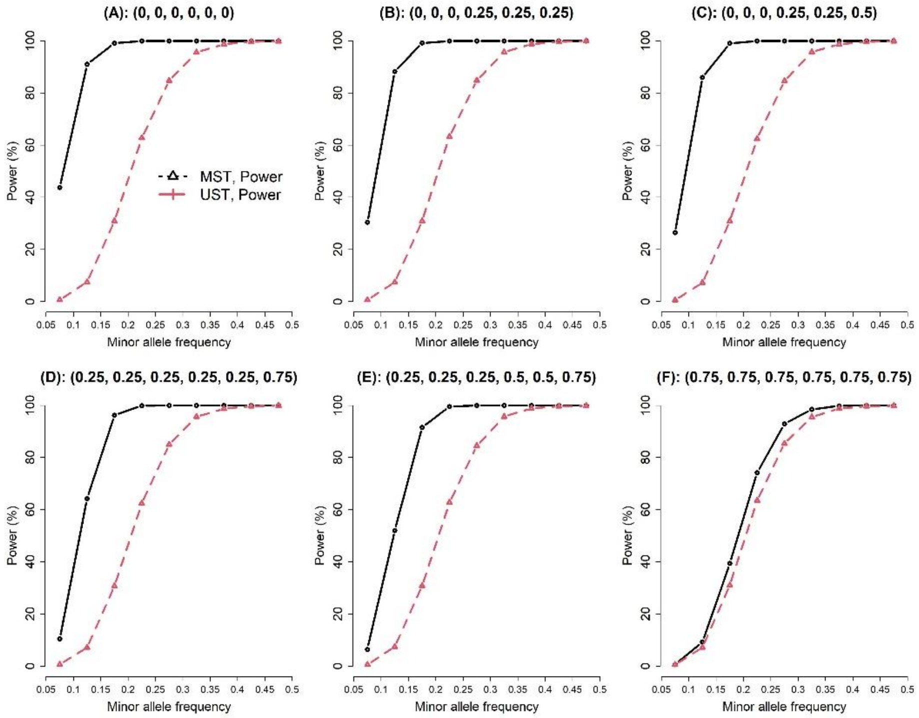

Figure S45. **Power of testing GxE for individual traits, when GxE influenced all four traits (four traits, each from a Chi-squared distribution with the degree of freedom 1, exposure prevalence = 0.5, and *n* = 93,708).**

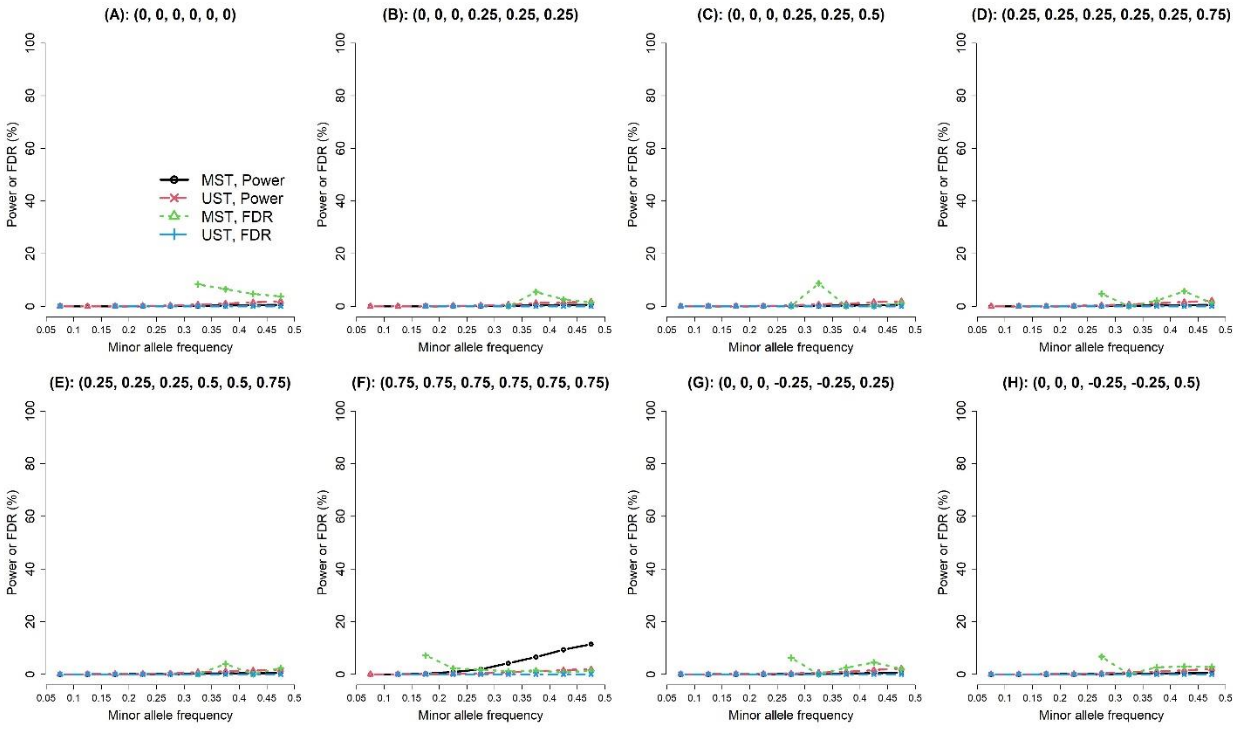

Figure S46. **Power and false discovery rate (FDR) of testing GxE for individual traits, when GxE influenced the first trait (four traits from a multivariate normal distribution, exposure prevalence = 0.2, and *n* = 25,200).** Some FDR levels were missing values due to no discoveries.

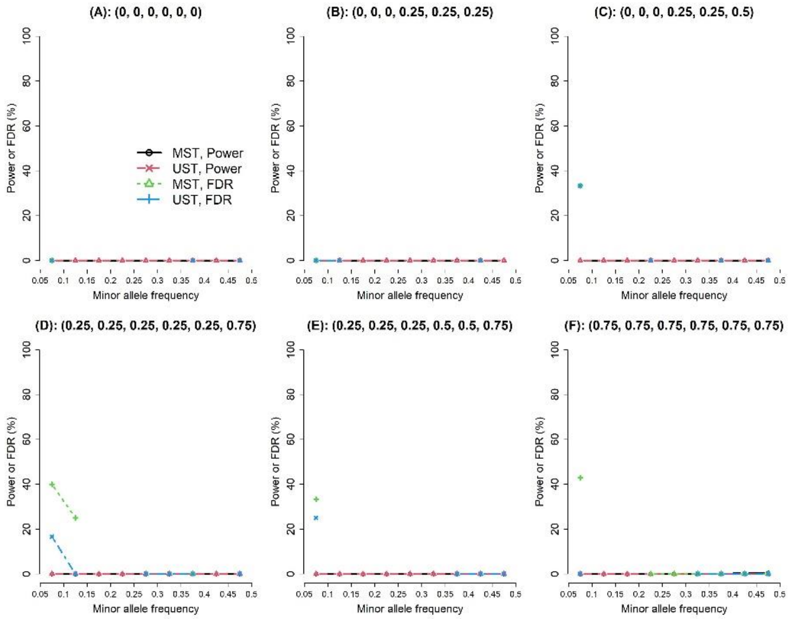

Figure S47. **Power and false discovery rate (FDR) of testing GxE for individual traits, when GxE influenced the first trait (four traits, each from a Chi-squared distribution with the degree of freedom 1, exposure prevalence = 0.2, and *n* = 25,200).** Some FDR levels were missing values due to no discoveries. Low power = > very few discoveries => FDR changed dramatically.

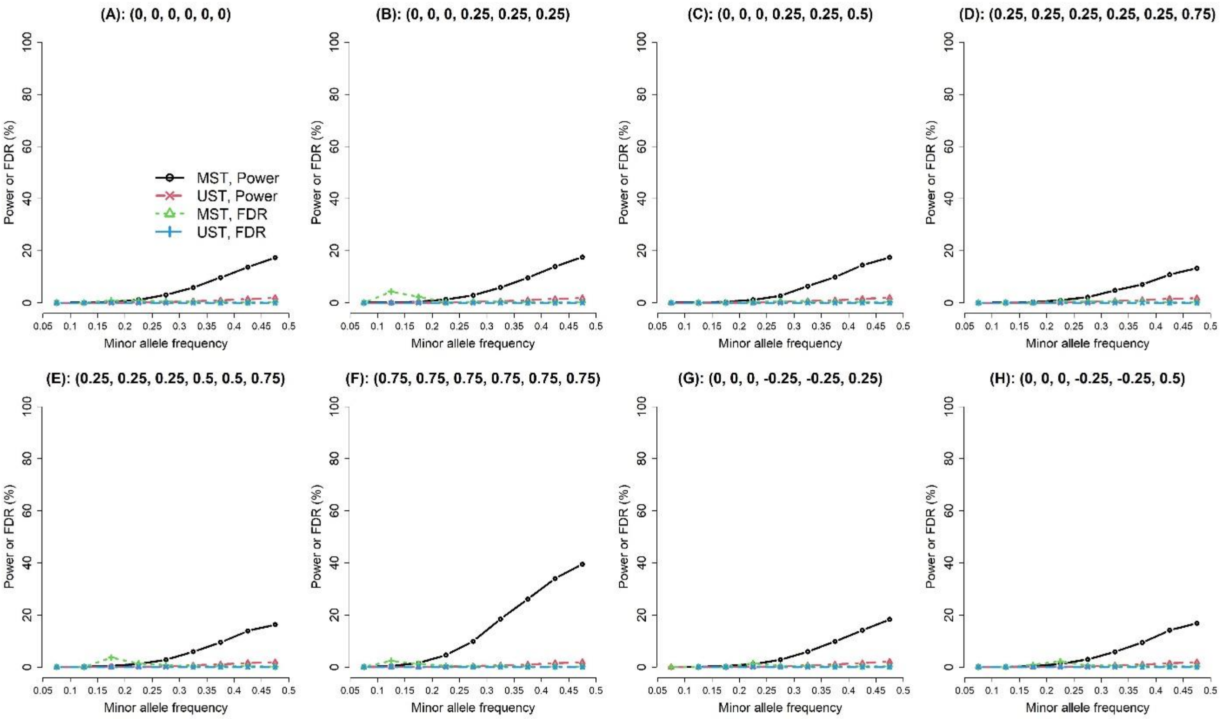

Figure S48. **Power and false discovery rate (FDR) of testing GxE for individual traits, when GxE influenced the first and the second traits (four traits from a multivariate normal distribution, exposure prevalence = 0.2, and *n* = 25,200).**

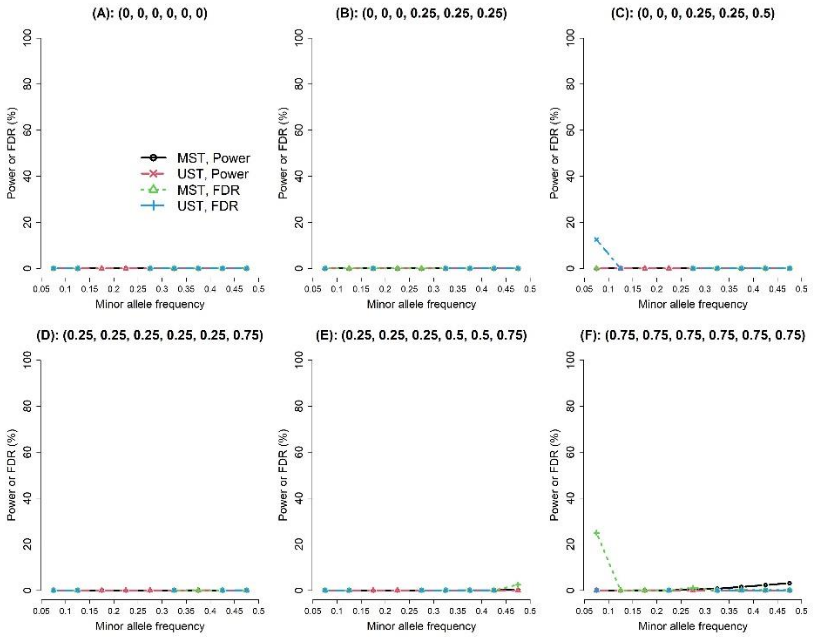

Figure S49. **Power and false discovery rate (FDR) of testing GxE for individual traits, when GxE influenced the first and the second traits (four traits, each from a Chi-squared distribution with the degree of freedom 1, exposure prevalence = 0.2, and *n* = 25,200).** Some FDR levels were missing values due to no discoveries.

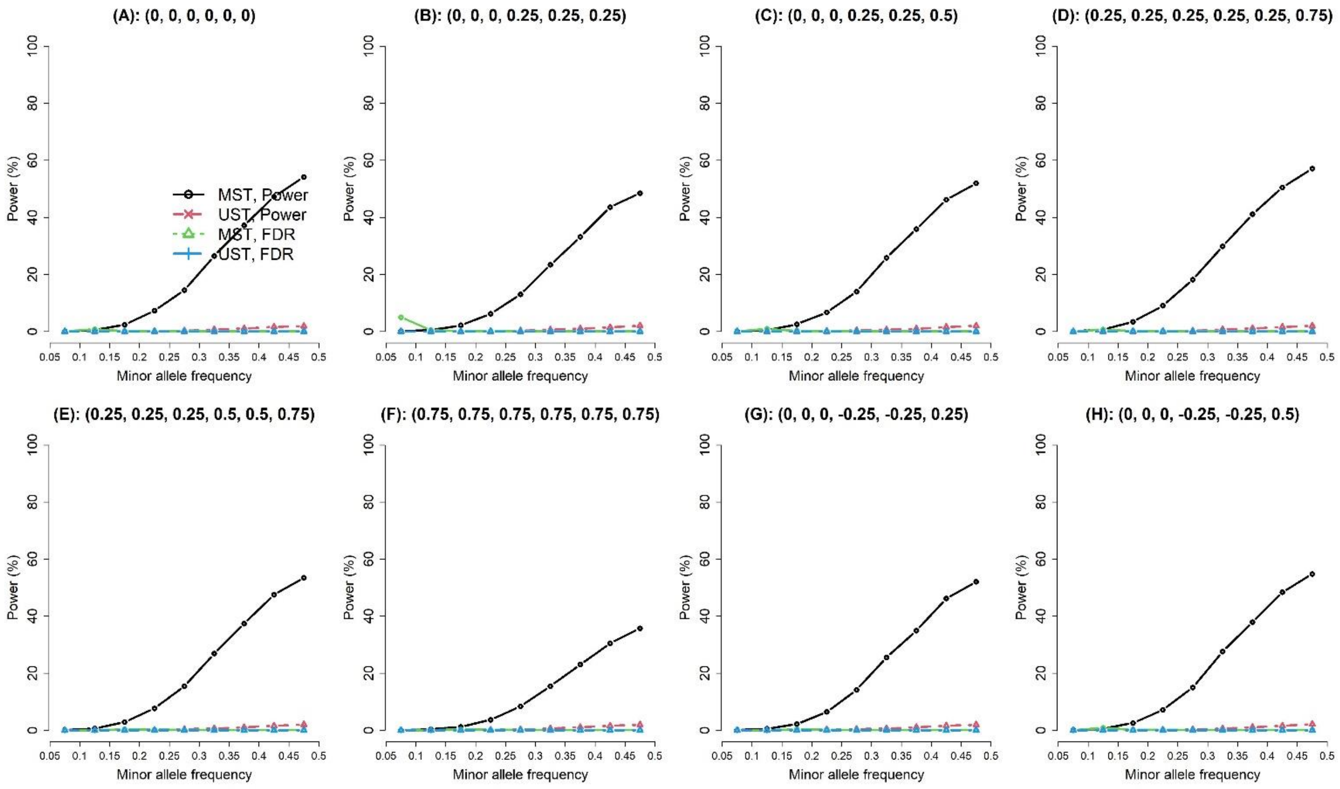

Figure S50. **Power and false discovery rate (FDR) of testing GxE for individual traits, when GxE influenced the first three traits (four traits from a multivariate normal distribution, exposure prevalence = 0.2, and *n* = 25,200).**

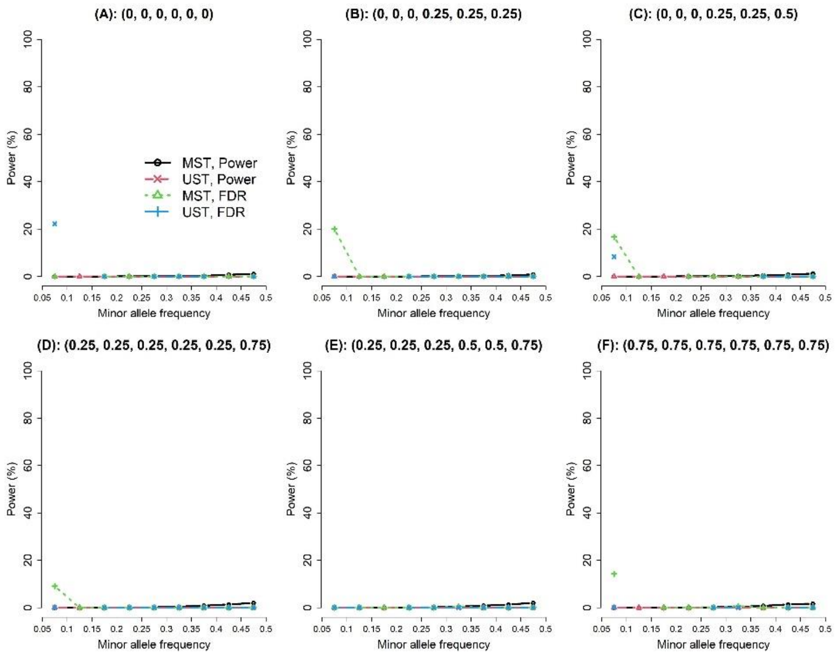

Figure S51. **Power and false discovery rate (FDR) of testing GxE for individual traits, when GxE influenced the first three traits (four traits, each from a Chi-squared distribution with the degree of freedom 1, exposure prevalence = 0.2, and *n* = 25,200).** Some FDR levels were missing values due to no discoveries.

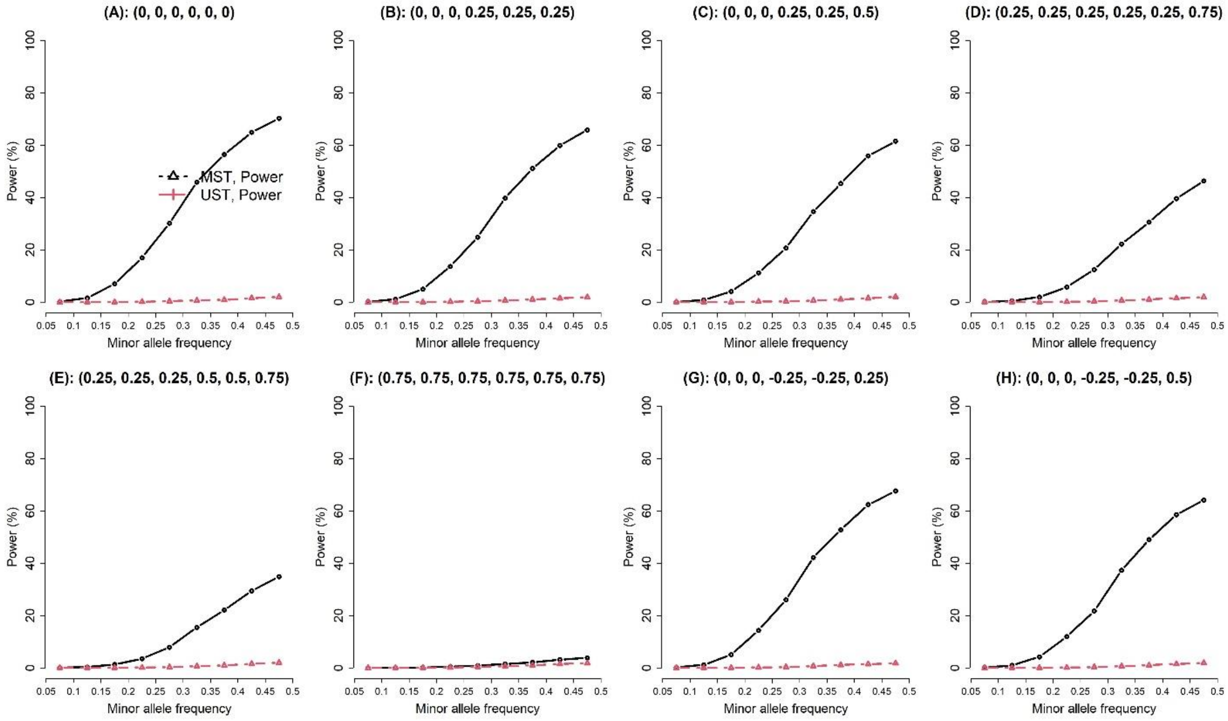

Figure S52. **Power of testing GxE for individual traits, when GxE influenced all four traits (four traits from a multivariate normal distribution, exposure prevalence = 0.2, and *n* = 25,200).**

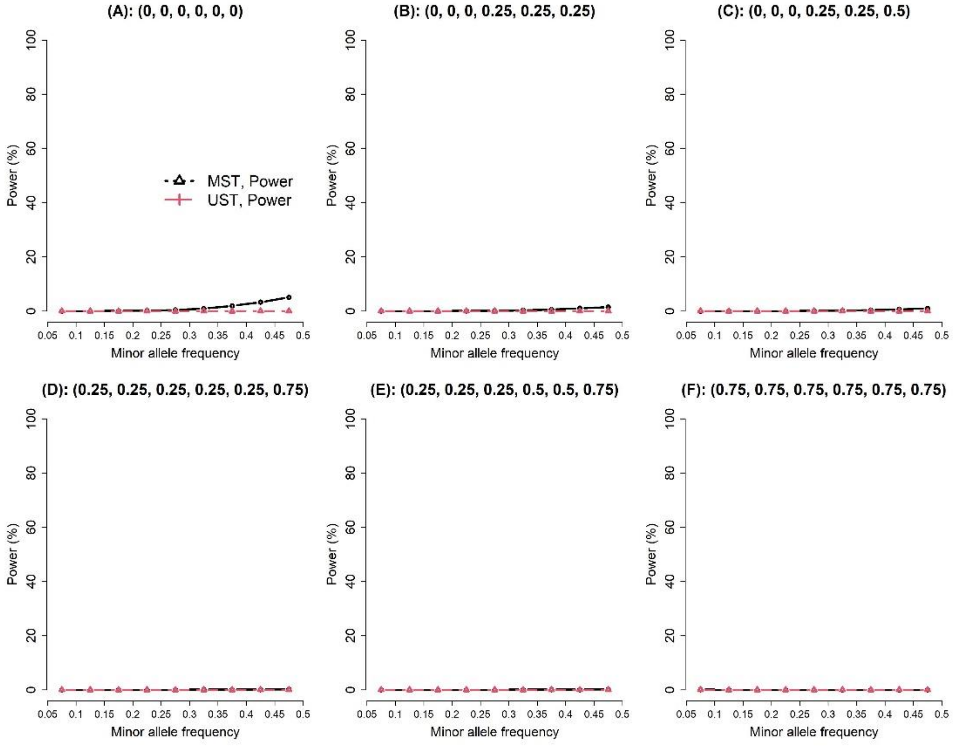

Figure S53. **Power of testing GxE for individual traits, when GxE influenced all four traits (four traits, each from a Chi-squared distribution with the degree of freedom 1, exposure prevalence = 0.2, and *n* = 25,200).**

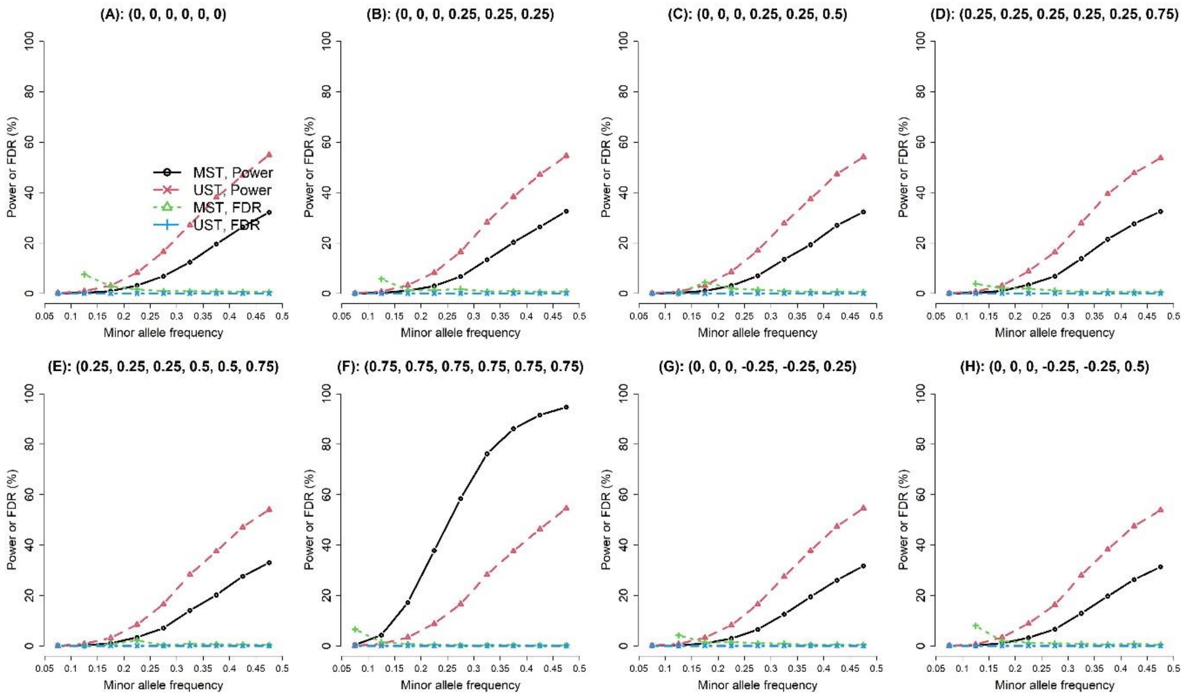

Figure S54. **Power and false discovery rate (FDR) of testing GxE for individual traits, when GxE influenced the first trait (four traits from a multivariate normal distribution, exposure prevalence = 0.5, and *n* = 25,200).** Some FDR levels were missing values due to no discoveries.

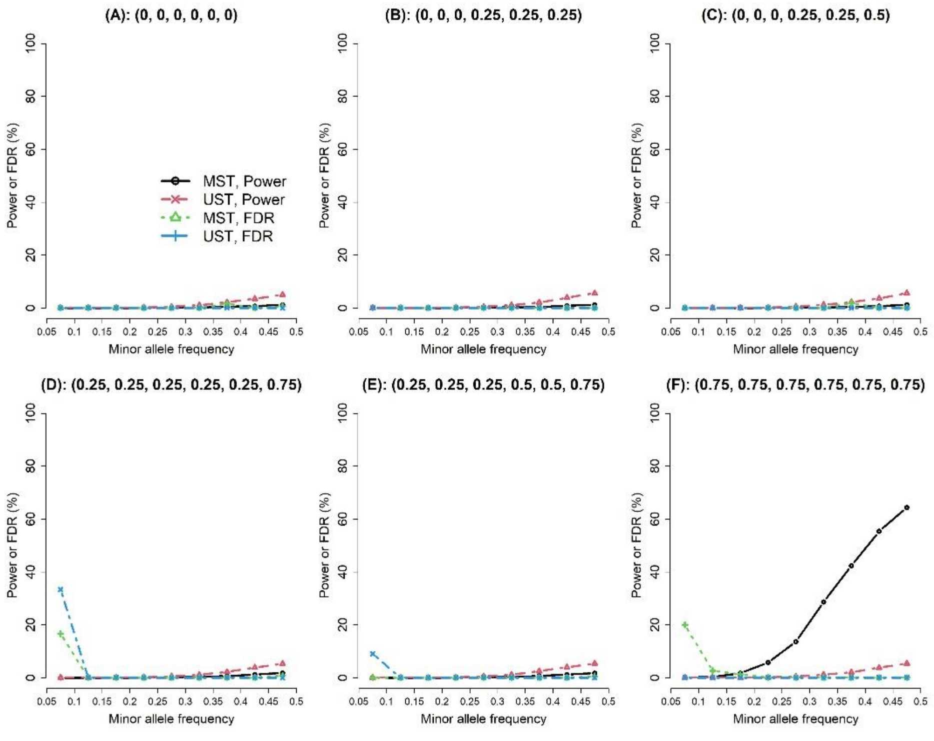

Figure S55. **Power and false discovery rate (FDR) of testing GxE for individual traits, when GxE influenced the first trait (four traits, each from a Chi-squared distribution with the degree of freedom 1, exposure prevalence = 0.5, and *n* = 25,200).** Some FDR levels were missing values due to no discoveries.

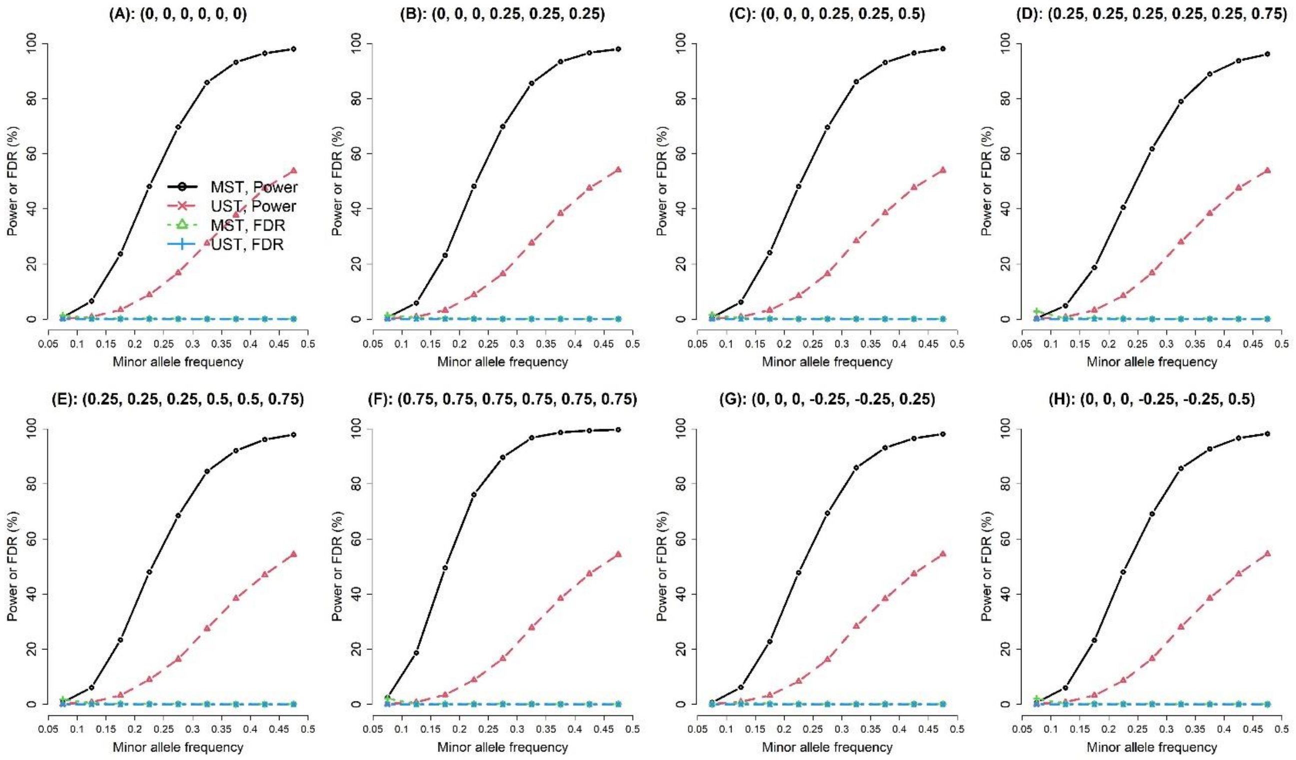

Figure S56. **Power and false discovery rate (FDR) of testing GxE for individual traits, when GxE influenced the first and the second traits (four traits from a multivariate normal distribution, exposure prevalence = 0.5, and *n* = 25,200).**

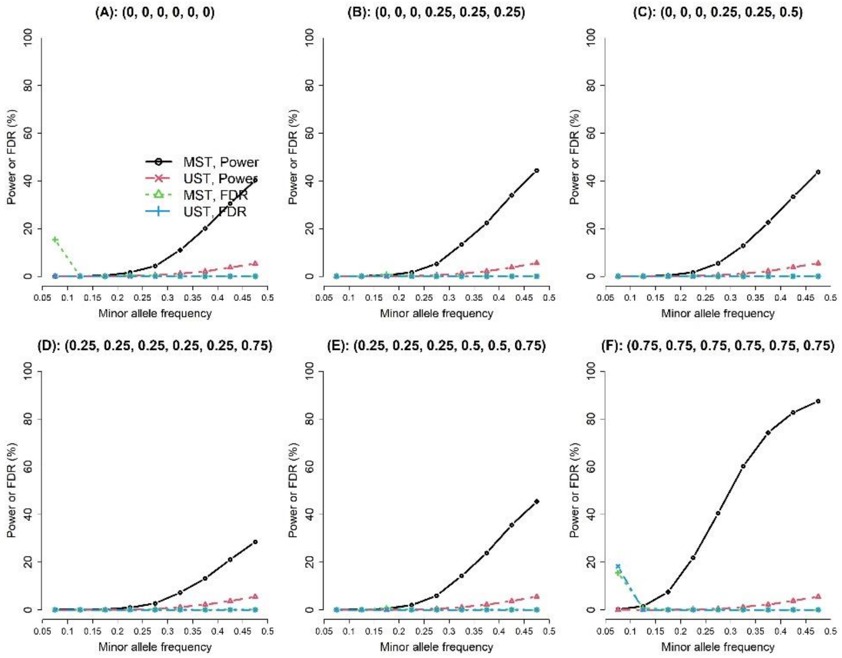

Figure S57. **Power and false discovery rate (FDR) of testing GxE for individual traits, when GxE influenced the first and the second traits (four traits, each from a Chi-squared distribution with the degree of freedom 1, exposure prevalence = 0.5, and *n* = 25,200).**

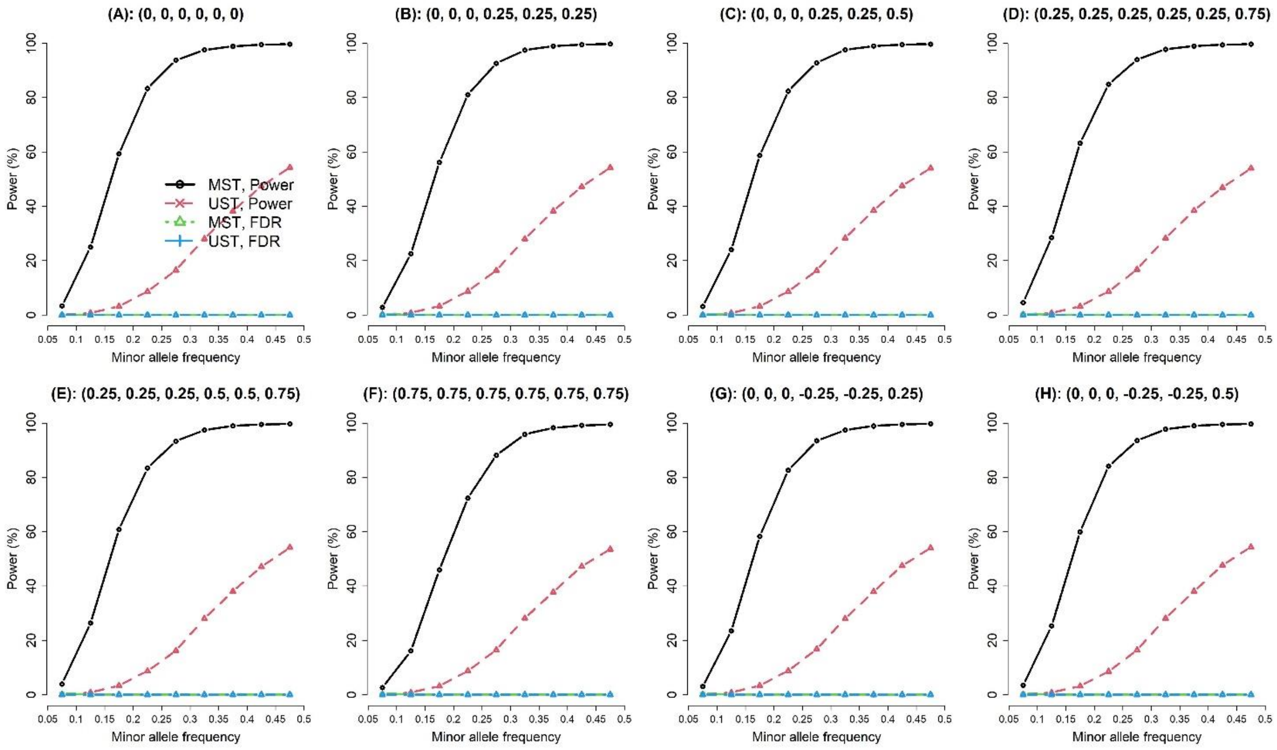

Figure S58. **Power and false discovery rate (FDR) of testing GxE for individual traits, when GxE influenced the first three traits (four traits from a multivariate normal distribution, exposure prevalence = 0.5, and *n* = 25,200).**

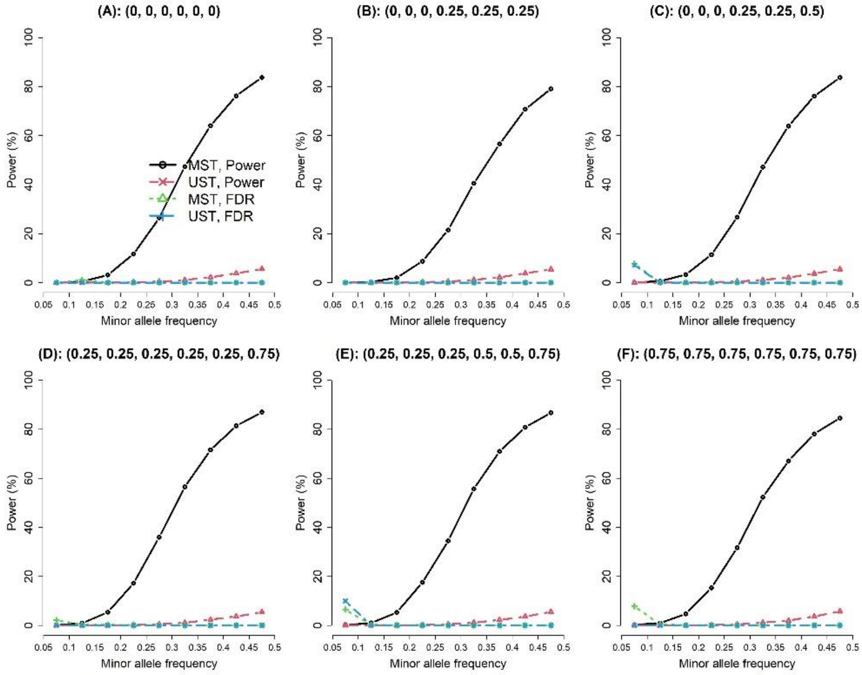

Figure S59. **Power and false discovery rate (FDR) of testing GxE for individual traits, when GxE influenced the first three traits (four traits, each from a Chi-squared distribution with the degree of freedom 1, exposure prevalence = 0.5, and *n* = 25,200).**

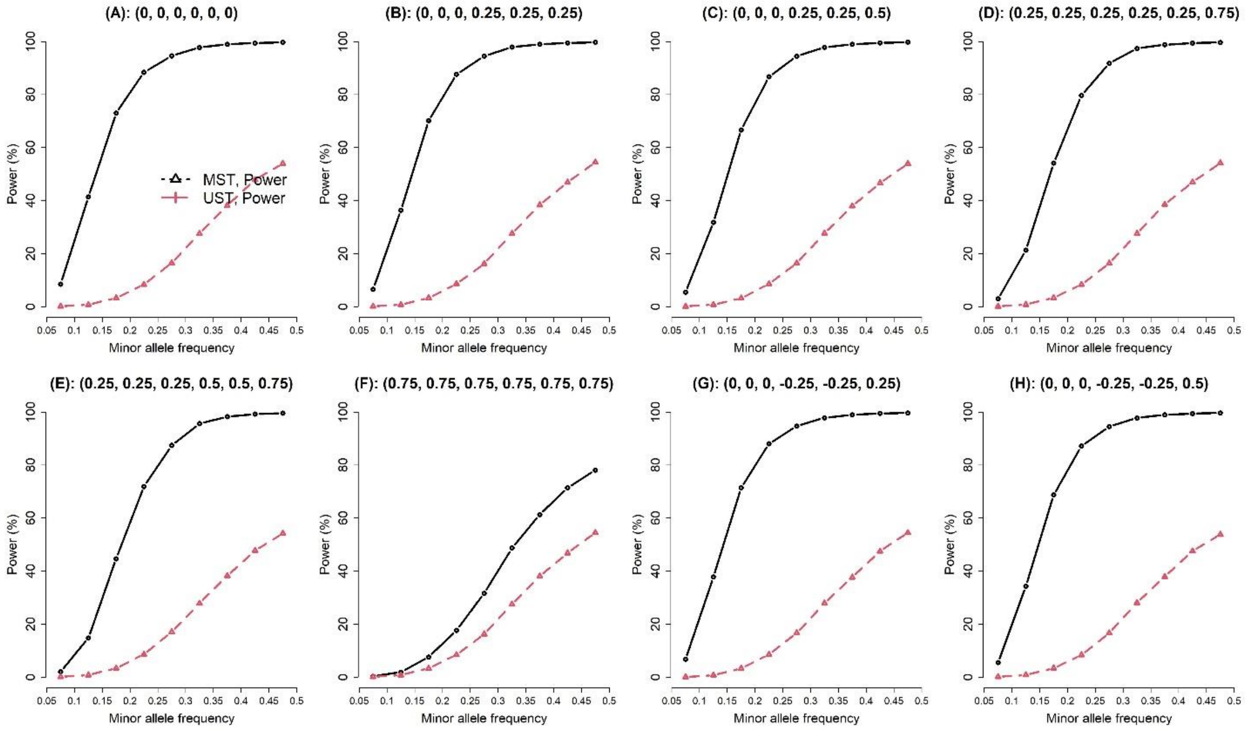

Figure S60. **Power of testing GxE for individual traits, when GxE influenced all four traits (four traits from a multivariate normal distribution, exposure prevalence = 0.5, and *n* = 25,200).**

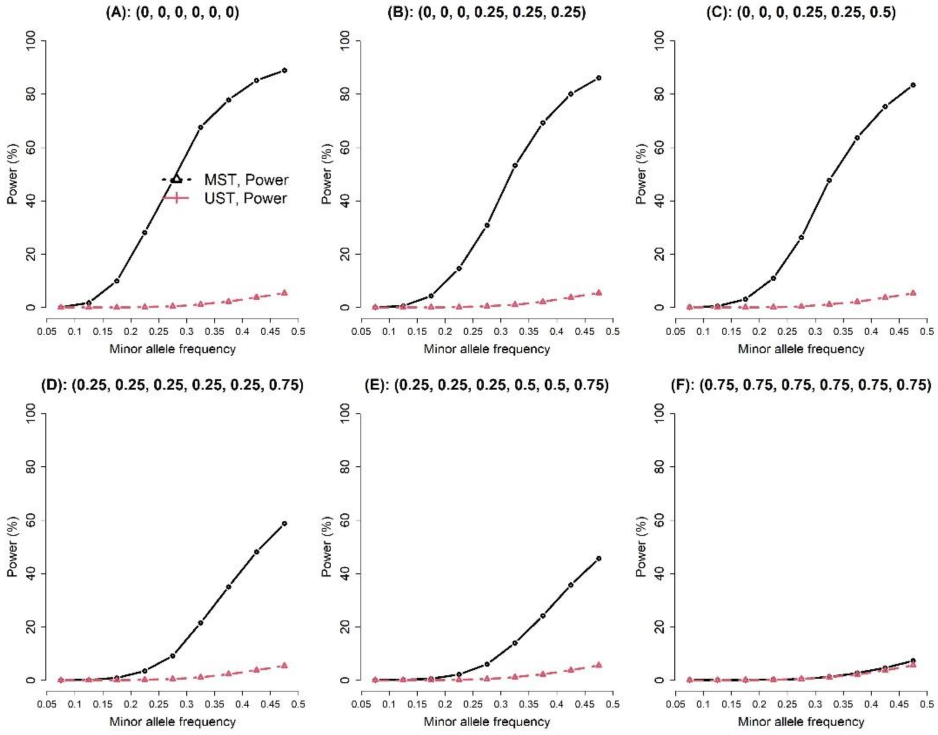

Figure S61. **Power of testing GxE for individual traits, when GxE influenced all four traits (four traits, each from a Chi-squared distribution with the degree of freedom 1, exposure prevalence = 0.5, and *n* = 25,200).**

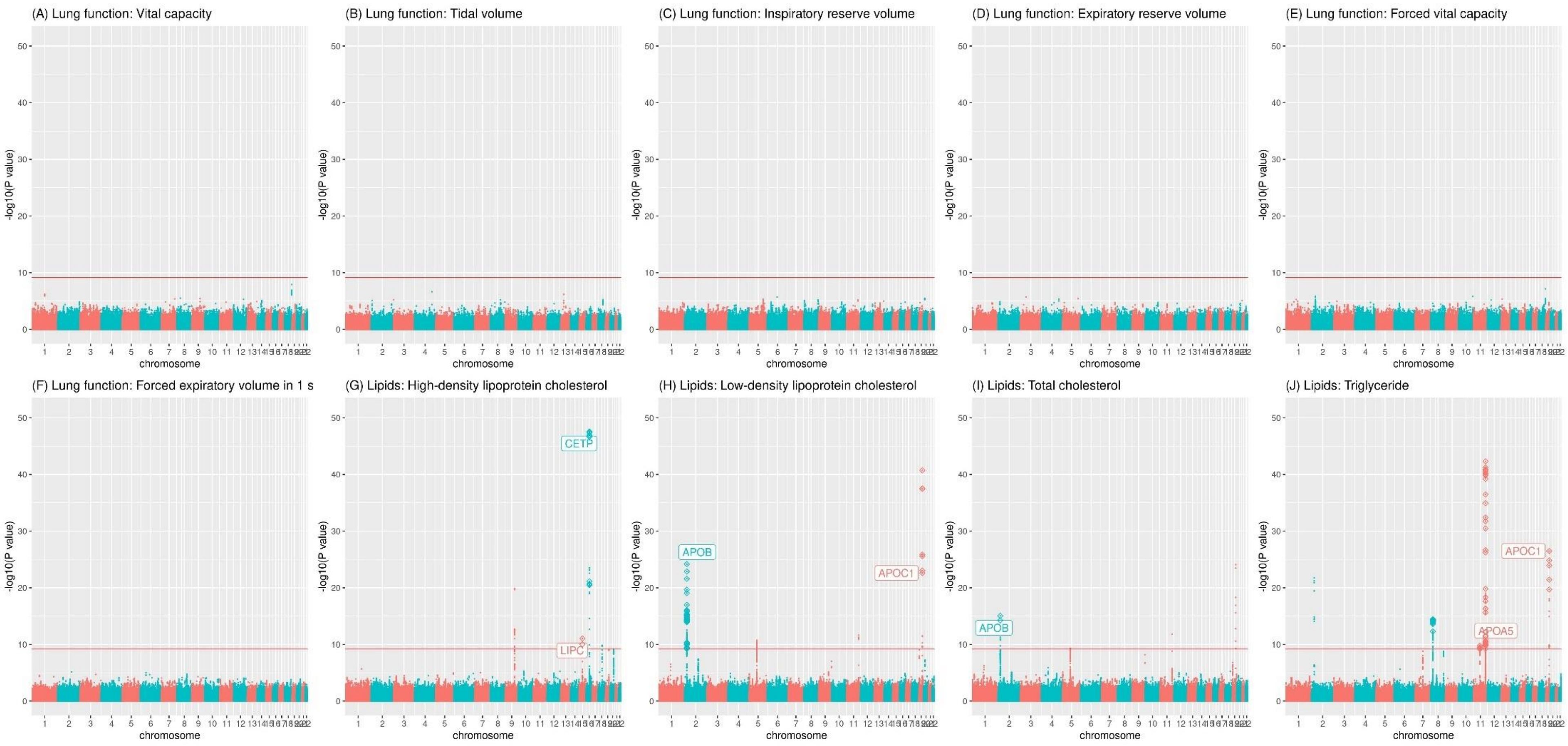

Figure S62. The Manhattan plots of the UST analysis on 6 TWB2 lung function traits and 4 TWB2 lipid traits

The red horizontal line denotes the experiment-wise significance level of 0.05/(2,570,487 × 29) = 6.7E-10, where 2,570,487 is the number of autosomal SNPs with MAF ≥ 0.05. I identified 1,904 vQTLs from all 29 TWB2 continuous traits (UST $p < 6.7E-10$) and sought replication from TWB1 if UST $p < 0.05/1,904 = 2.6E-5$. Gene names on this figure mark the vQTLs that were identified from TWB2 (UST $p < 6.7E-10$) and further replicated in TWB1 (UST $p < 0.05/1,904 = 2.6E-5$).

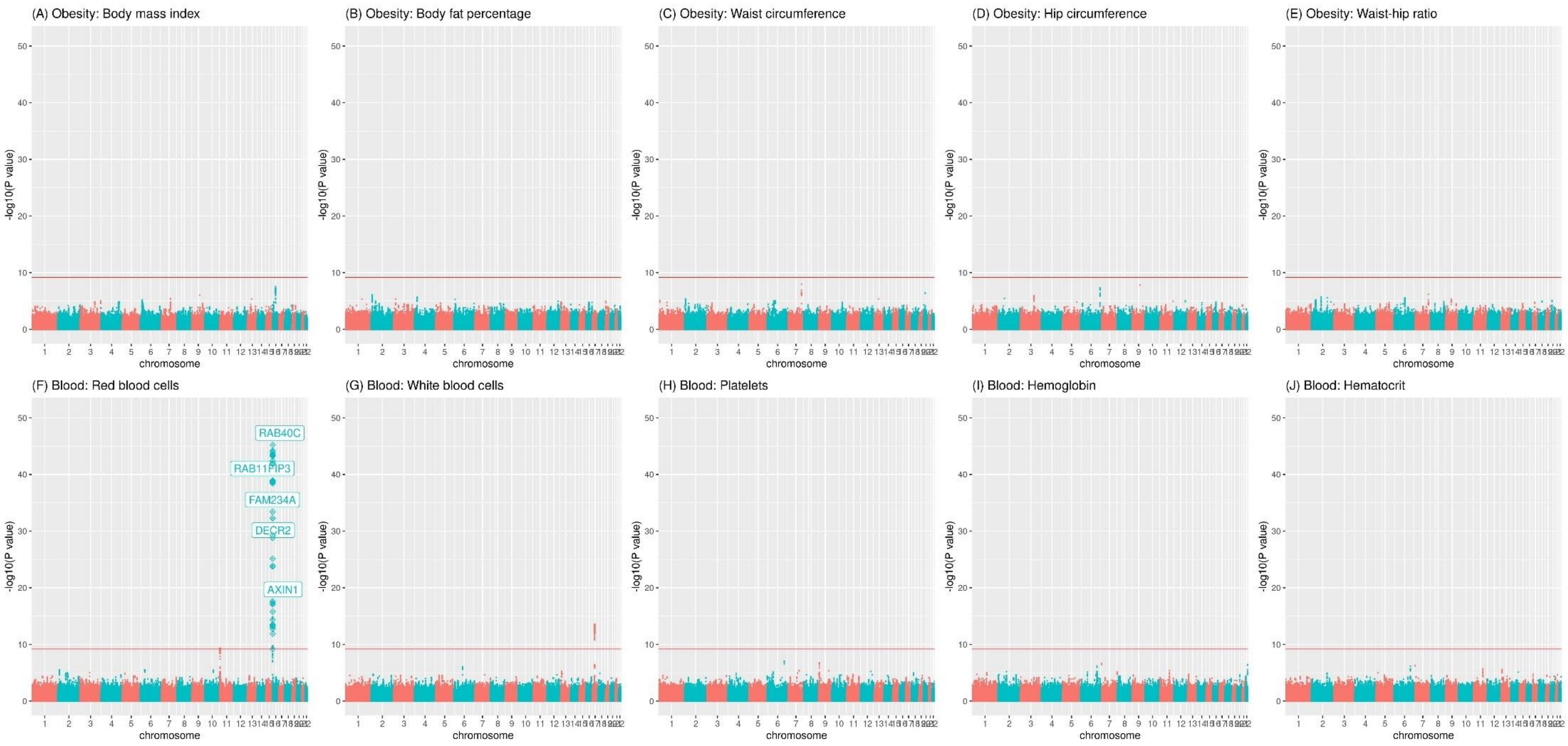

Figure S63. The Manhattan plots of the UST analysis on 5 TWB2 obesity traits and 5 TWB2 blood traits

The red horizontal line denotes the experiment-wise significance level of 0.05/(2,570,487 $\times$ 29) = 6.7E-10, where 2,570,487 is the number of autosomal SNPs with MAF $\geq$ 0.05. I identified 1,904 vQTLs from all 29 TWB2 continuous traits (UST p < 6.7E-10) and sought replication from TWB1 if UST p < 0.05/1,904 = 2.6E-5. Gene names on this figure mark the vQTLs that were identified from TWB2 (UST p < 6.7E-10) and further replicated in TWB1 (UST p < 0.05/1,904 = 2.6E-5).

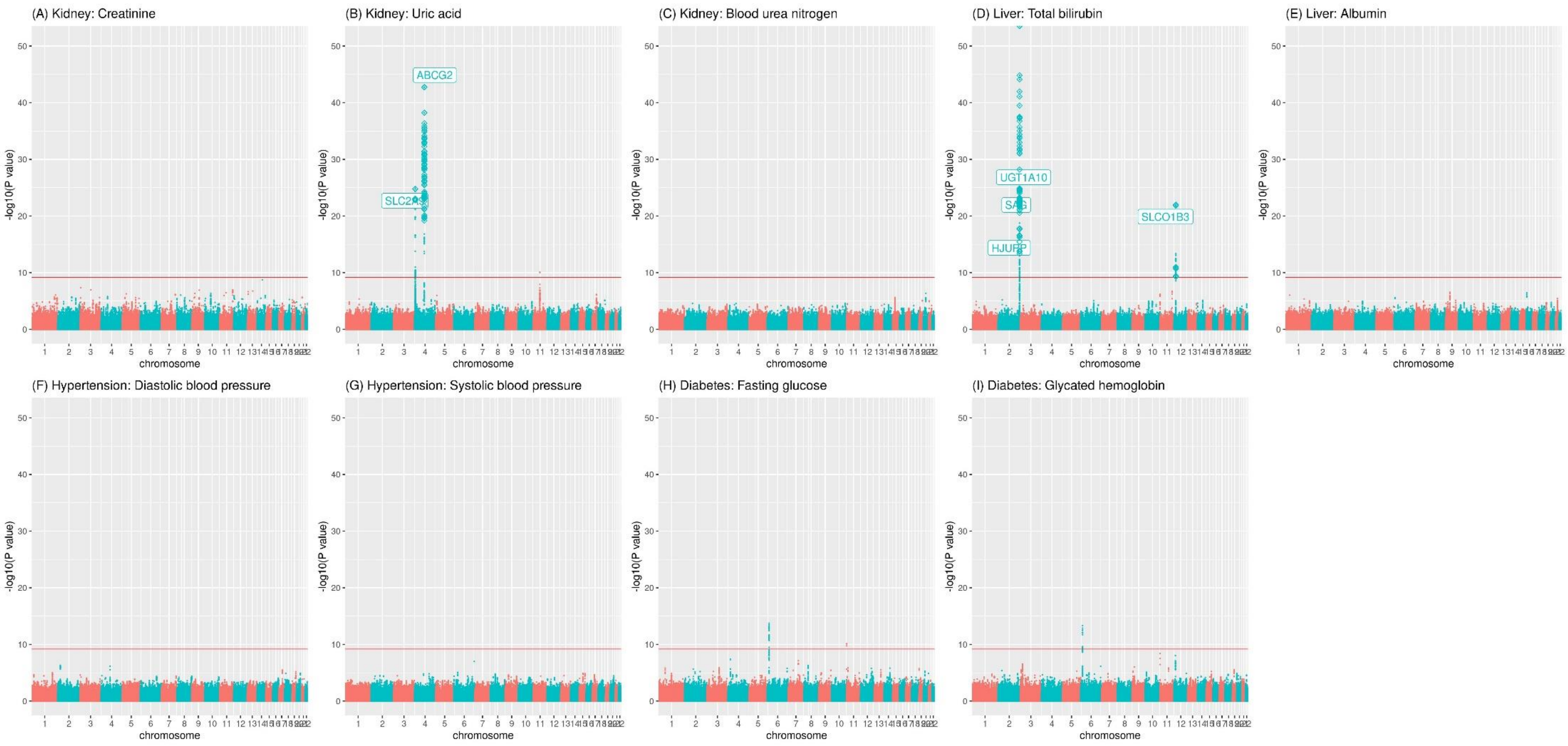

Figure S64. The Manhattan plots of the UST analysis on 3 TWB2 kidney traits, 2 liver traits, 2 hypertension traits, and 2 diabetes traits

The red horizontal line denotes the experiment-wise significance level of 0.05/(2,570,487 × 29) = 6.7E-10, where 2,570,487 is the number of autosomal SNPs with MAF ≥ 0.05. I identified 1,904 vQTLs from all 29 TWB2 continuous traits (UST p < 6.7E-10) and sought replication from TWB1 if UST p < 0.05/1,904 = 2.6E-5. Gene names on this figure mark the vQTLs that were identified from TWB2 (UST p < 6.7E-10) and further replicated in TWB1 (UST p < 0.05/1,904 = 2.6E-5).

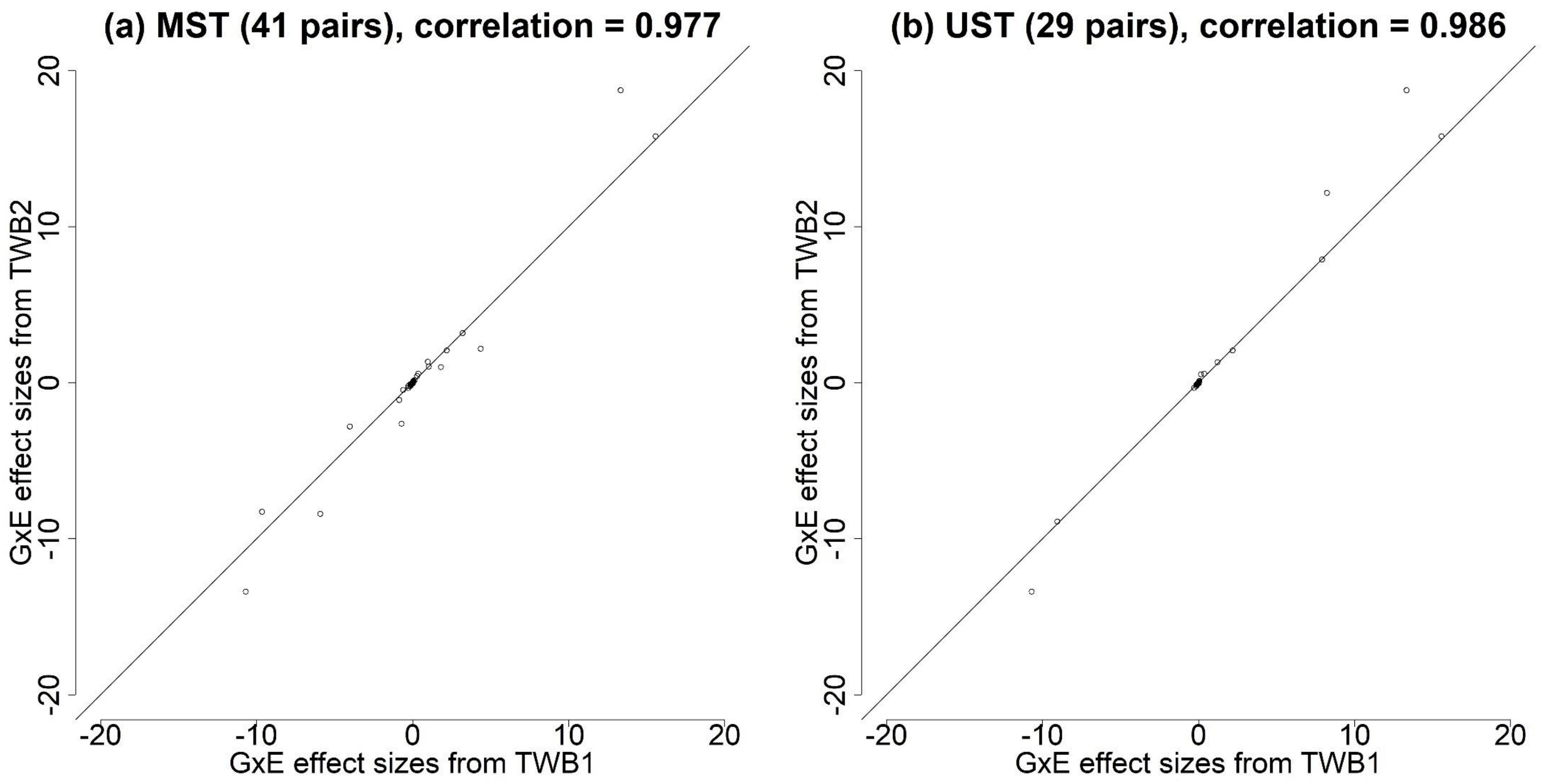

Figure S65. The scatter plots of the GxE effect sizes from the discovery (TWB2) and replication (TWB1) cohorts

(a) The MST 41 pairs of the GxE effect sizes were from the 41 "*" of Figure 5 left column (MST). The Pearson's correlation coefficient of the 41 pairs of GxE effect sizes was 0.977. The straight line indicates $x = y$.

(b) The UST 29 pairs of the GxE effect sizes were from the 29 "*" of Figure 5 right column (UST). The Pearson's correlation coefficient of the 29 pairs of GxE effect sizes was 0.986. The straight line indicates $x = y$.
